# Supplementary material for: Reductional Meiosis I Chromosome Segregation Is Established by Coordination of Key Meiotic Kinases
Source: Dev Cell. 2019 May 20;49(4):526–541.e5. doi: 10.1016/j.devcel.2019.04.003 (PMC6547162; doi:10.1016/j.devcel.2019.04.003)
Supplement: Document S1. Figures S1–S7 and Tables S2 and S3 [file mmc1.pdf]

Developmental Cell, Volume *49*

Supplemental Information

Reductional Meiosis I Chromosome Segregation Is Established by Coordination of Key Meiotic Kinases

Stefan Galander, Rachael E. Barton, Weronika E. Borek, Christos Spanos, David A. Kelly, Daniel Robertson, Juri Rappsilber, and Adèle L. Marston

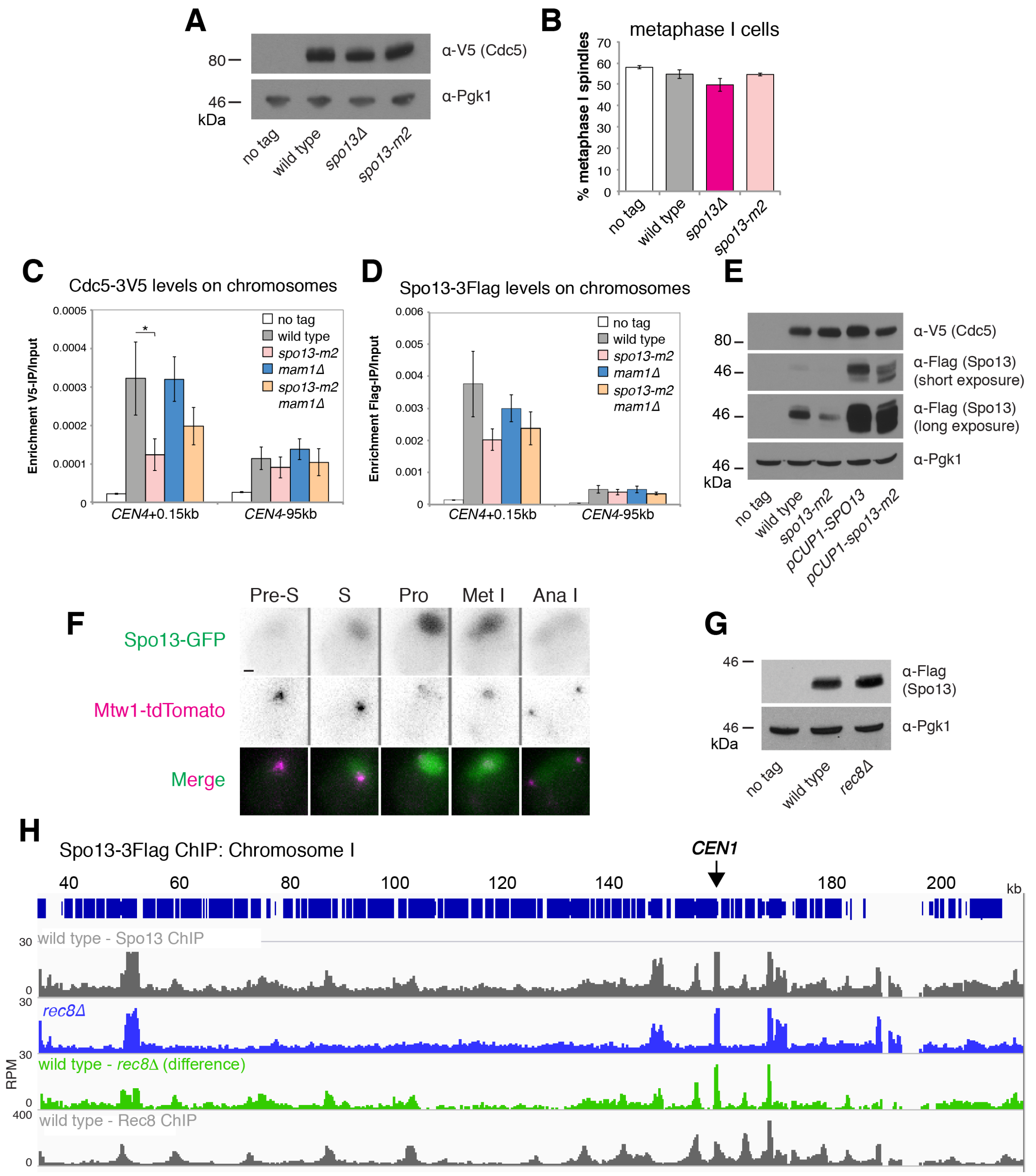

Figure S1. Spo13 and Cdc5 chromosome enrichment and cellular levels in monoorientation mutants, related to Figure 1. (A-B) Cdc5 levels and metaphase I arrest efficiency are comparable for wild type, *spo13Δ* and *spo13-m2* in the experiment in Figure 1A. (A) Immunoblots from a representative experiment show Cdc5-3V5 levels in whole cell extracts. (B) Percentage of cells with metaphase I spindles after tubulin immunofluorescence (n = 200). (C-D) Loss of centromeric Cdc5 in *spo13-m2* mutants is not caused by defective monoorientation. Mean enrichment values of Cdc5-3V5 (C) and Spo13-3Flag (D) in metaphase I with standard error bars (n = 4) (*p<0.05). (E) Immunoblot confirms Spo13 overproduction and the effect on Cdc5 levels in whole cell extracts for the experiments shown in Figure 1B and C. (F) Spo13 is present throughout the nucleus from S phase until anaphase I. Representative images of wild-type cells carrying Spo13-GFP and Mtw1-tdTomato undergoing meiosis. Scale bar, 1 µm. (G-H) Spo13 levels are comparable in wild type and *rec8Δ*, despite Rec8 being required for the association of Spo13 with some chromosomal sites. Data is from the experiment in Figures 1D and 1E. Cells were arrested in prophase. (G) Immunoblot shows Spo13 levels are comparable in wild type and *rec8Δ*. (H) ChIP-seq enrichment of Spo13-3Flag on chromosome I is shown with a different scale on the y axis to show Spo13 enrichment sites on chromosome arms. Pgk1 serves as loading control in A, E and G.

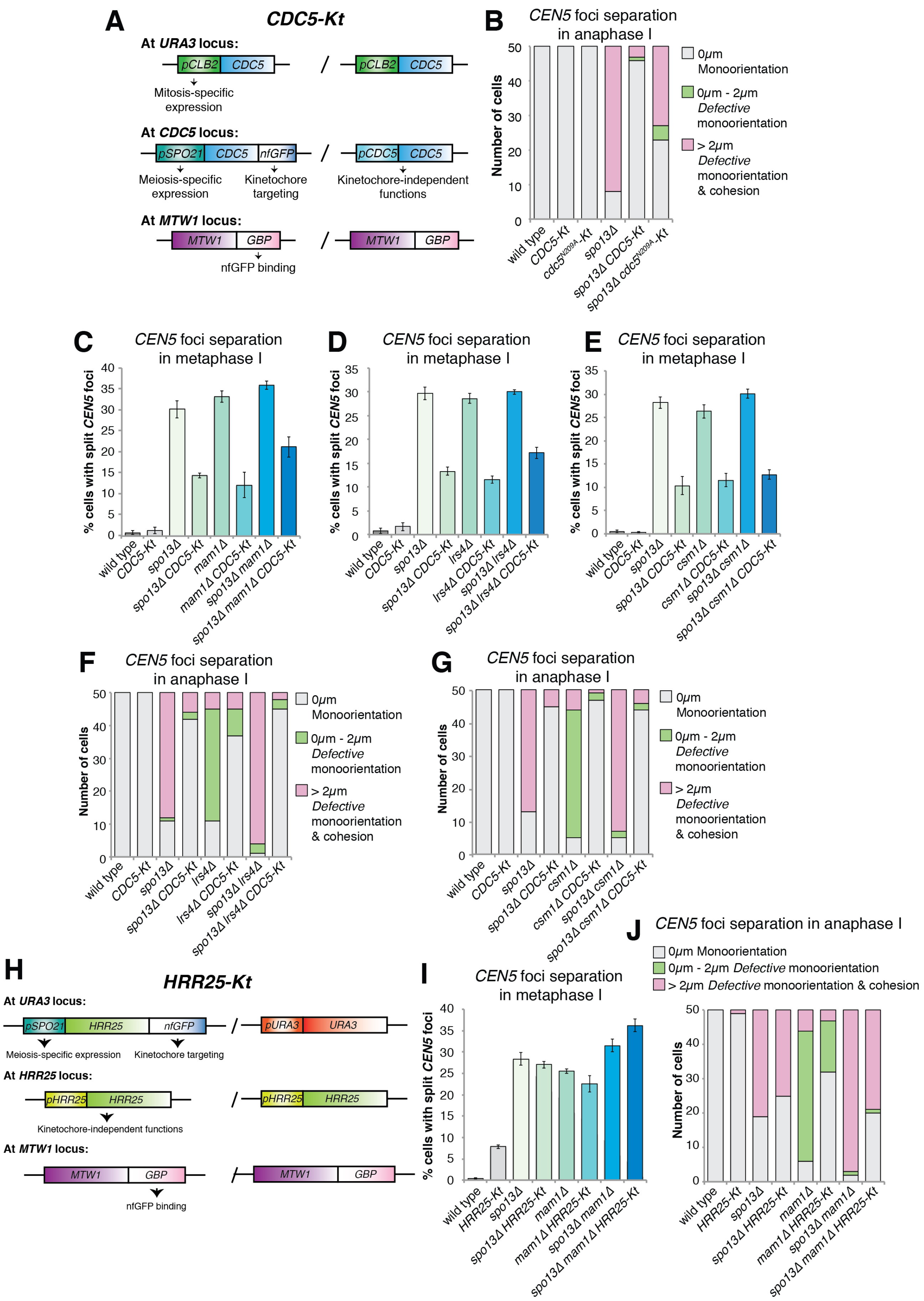

Figure S2. Cdc5-Kt, but not Hrr25-Kt, rescues monoorientation in *spo13Δ* cells, related to Figure 2. (A) Schematic representation of Cdc5 tethering setup within the genome. Cdc5 tagged with a non-

fluorescent version of GFP (Cdc5-nfGFP) (Kutrowska et al., 2007) was produced from the meiosis-specific promoter *pSPO21* and the kinetochore protein Mtw1 was tagged with GFP-binding protein (GBP), a nanobody that specifically recognizes the GFP protein (Rothbauer et al., 2006). These Cdc5-kinetochore bound cells (Cdc5-Kt) also produced untagged Cdc5 from the endogenous promoter to allow its access to other meiotic targets. (B) Kinase-defective Cdc5-Kt does not rescue the monoorientation phenotype of *spo13Δ* mutants. Frequency of *CEN5* distance categories as shown in Figure 2D is shown for the indicated genotypes after live-cell imaging with the maximum distance between two TetR-tdTomato foci measured as in Figure 2E. (C-E) Cdc5-Kt partially rescues the monoorientation defects caused by absence of monopolin complex components Mam1 (C), Lrs4 (D) and Csm1 (E). The number of cells with two distinct tdTomato foci in metaphase-I-arrested cells was scored (n = 200). Graphs show mean values of three experimental replicates with standard error bars. For panel (C), data from Figure 2B was duplicated. (F-G) Co-segregation of sister chromatids is rescued by Cdc5-Kt in *lrs4Δ* (F) and *csm1Δ* (G) mutants. Frequency of *CEN5* distance categories as shown in Figure 2D is shown for the indicated genotypes after live-cell imaging with the maximum distance between two TetR-tdTomato foci measured as in Figure 2E. (H-J) Hrr25-Kt partially rescues the monoorientation defect caused by absence of Mam1, but not Spo13. (H) Schematic representation of Hrr25 tethering setup within the genome. (I) The number of cells with two distinct tdTomato foci in metaphase-I-arrested cells was scored. Graphs show mean values of three experimental replicates with standard error bars. (J) The maximum distance between two TetR-tdTomato foci was measured within two time points after the first round of Cdc14 release for 50 cells.

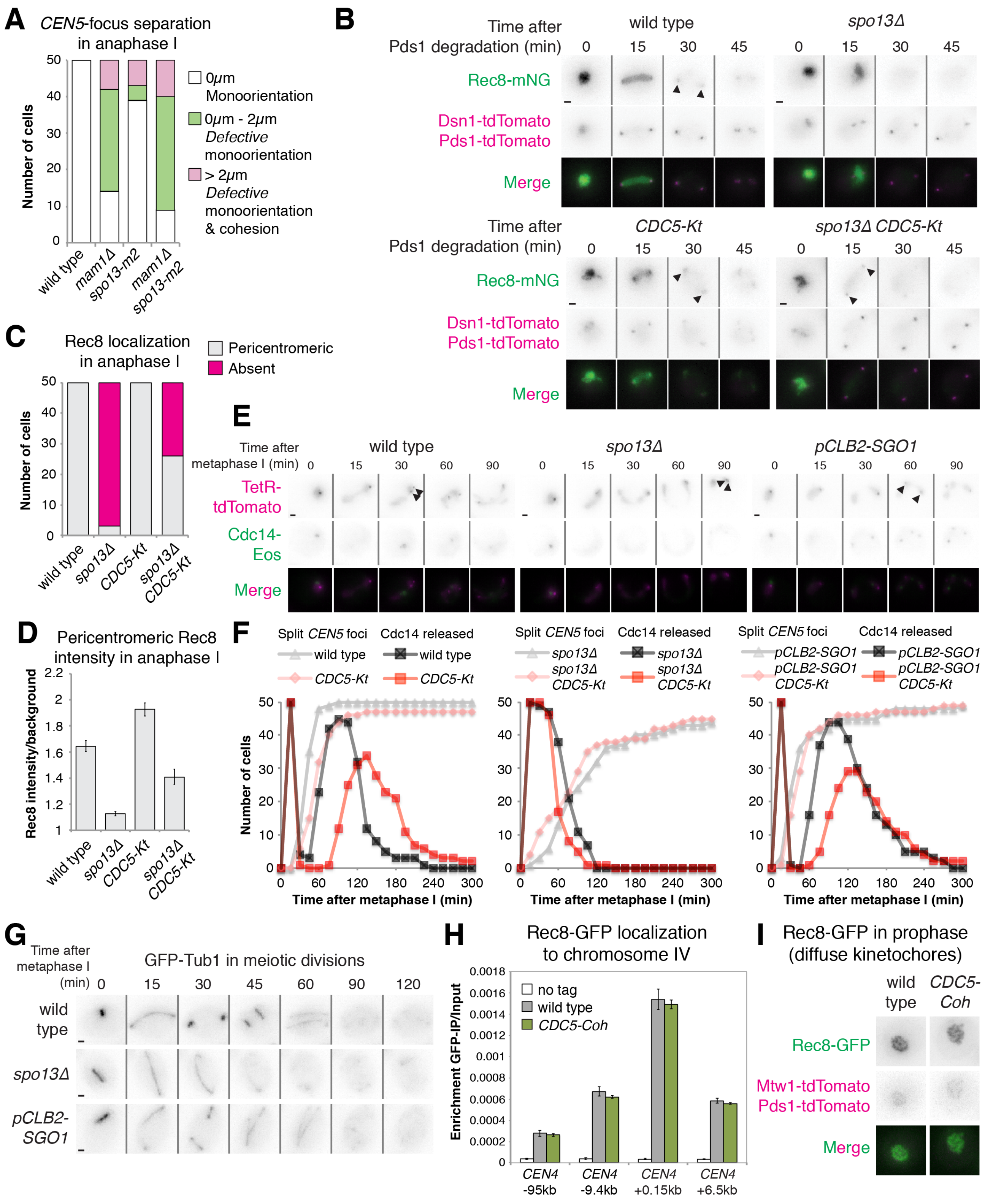

Figure S3. Cdc5-Kt does not restore cohesion in the absence of *SPO13*, related to Figure 3. (A) Effect of *spo13-m2* on monoorientation and cohesin protection. Frequency of *CEN5* distance categories after measurement of the maximum distance between two TetR-GFP foci in the time interval between Pds1-tdTomato degradation and spindle pole body duplication after live-cell imaging of 50 cells. (B-D) Cdc5-Kt increases pericentromeric cohesin in anaphase I. (B) Representative images from movies of cells carrying Rec8-mNG, Dsn1-tdTomato and Pds1-tdTomato with the indicated genotypes are shown. Scale bars represent 1μm. Arrows indicate pericentromeric Rec8-mNG. (C) Frequency of cells with the indicated pattern of Rec8-mNG localization is shown for the indicated genotypes. (D) The average intensity of pericentromeric Rec8-mNG is shown for the indicated genotypes with standard error bars. (E-F) Sister chromatid cohesion is lost in *spo13Δ CDC5-Kt* cells after anaphase I onset. Sgo1-depleted cells are shown as

a control. (E) Representative movies from live cells carrying heterozygous *CEN5*-tdTomato foci and Cdc14-mEos. Arrowheads indicate split *CEN5*-tdTomato foci. (F) Movies were scored for the presence of two *CEN5*-tdTomato foci and Cdc14 release for 300 minutes after metaphase I (time point before first Cdc14 release) at 15 min intervals. For *CEN5* foci separation, the accumulation of cells splitting foci at least once is shown. Only cells with co-segregating sister chromatids in anaphase I were scored. Note that while wild-type and *pCLB2-SGO1* cells undergo two rounds of Cdc14 release, *spo13Δ* cells exhibit only a single release of Cdc14. (G) *CEN5* foci separation in *spo13Δ* cells after anaphase I is not due to spindle forces since no metaphase II spindle forms. Representative images of meiotic spindles (GFP-Tub1) from the indicated genotypes. In contrast, meiosis II spindles form in wild type and *pCLB2-SGO1* and can account for *CEN5* foci splitting. (H-I) Rec8 loading is comparable in wild-type and *CDC5-Coh* strains. (H) ChIP-qPCR of Rec8-GFP prior to prophase. Cells were all *ndt80Δ* background and harvested for ChIP 6h after inducing sporulation. Mean values from three independent biological repeats are shown with standard error bars. (I) Representative images of Rec8-GFP from wild-type and CDC5-Coh cells in prophase, as judged by dispersed kinetochore protein Mtw1-tdTomato.

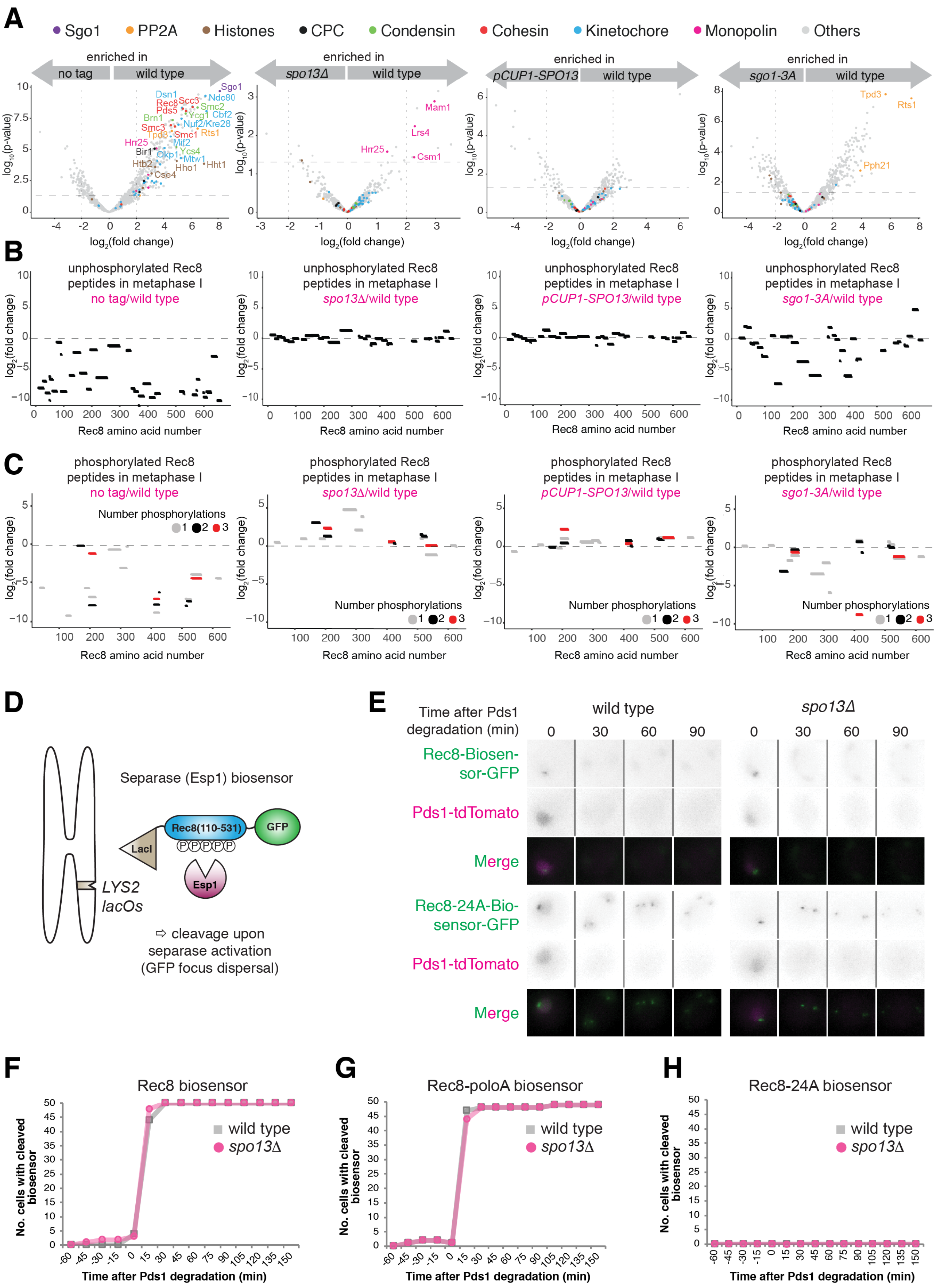

Figure S4. Spo13 and cohesin phosphorylation, related to Figure 5. (A) The pericentromeric proteome is largely unchanged upon deletion or overexpression of *SPO13*. Immunoprecipitates from Figures 5A and 5B were analysed for interacting proteins by mass spectrometry. Enrichment of proteins in wild-type cells compared with no tag, *spo13Δ*, *sgo1-3A* and *SPO13* overexpression is shown. Proteins belonging to distinct

pericentromeric protein complexes are coloured as indicated. (B-C) Cohesin phosphorylation in metaphase I is largely unaffected in the absence of *SPO13*. Analysis of unphosphorylated (B) and phosphorylated (C) Rec8 peptides co-precipitated with Sgo1-3V5, presented as relative intensities. Each bar indicates a single Rec8 peptide with amino acids of Rec8 indicated on the x-axis. For (C) mono-, di- and tri-phosphorylated forms of the same peptide are indicated by differently coloured bars. (D-H) *rec8-24A*, but not *rec8-poloA*, is a poor substrate for cleavage by separase in wild-type and *spo13Δ* cells. (D) Schematic illustration of a separase biosensor. (E) Representative images of separase biosensor cleavage in wild-type and *spo13Δ* cells. Cells with either a wild-type Rec8 or Rec8-24A biosensor fragment are shown to illustrate cleavage or non-cleavage upon Pds1 degradation. (F-H) Cells carrying a Rec8 (F), Rec8-poloA (G) or Rec8-24A (H) separase biosensor were followed by live-cell imaging and scored for diffusion of the biosensor focus at 15 min intervals.

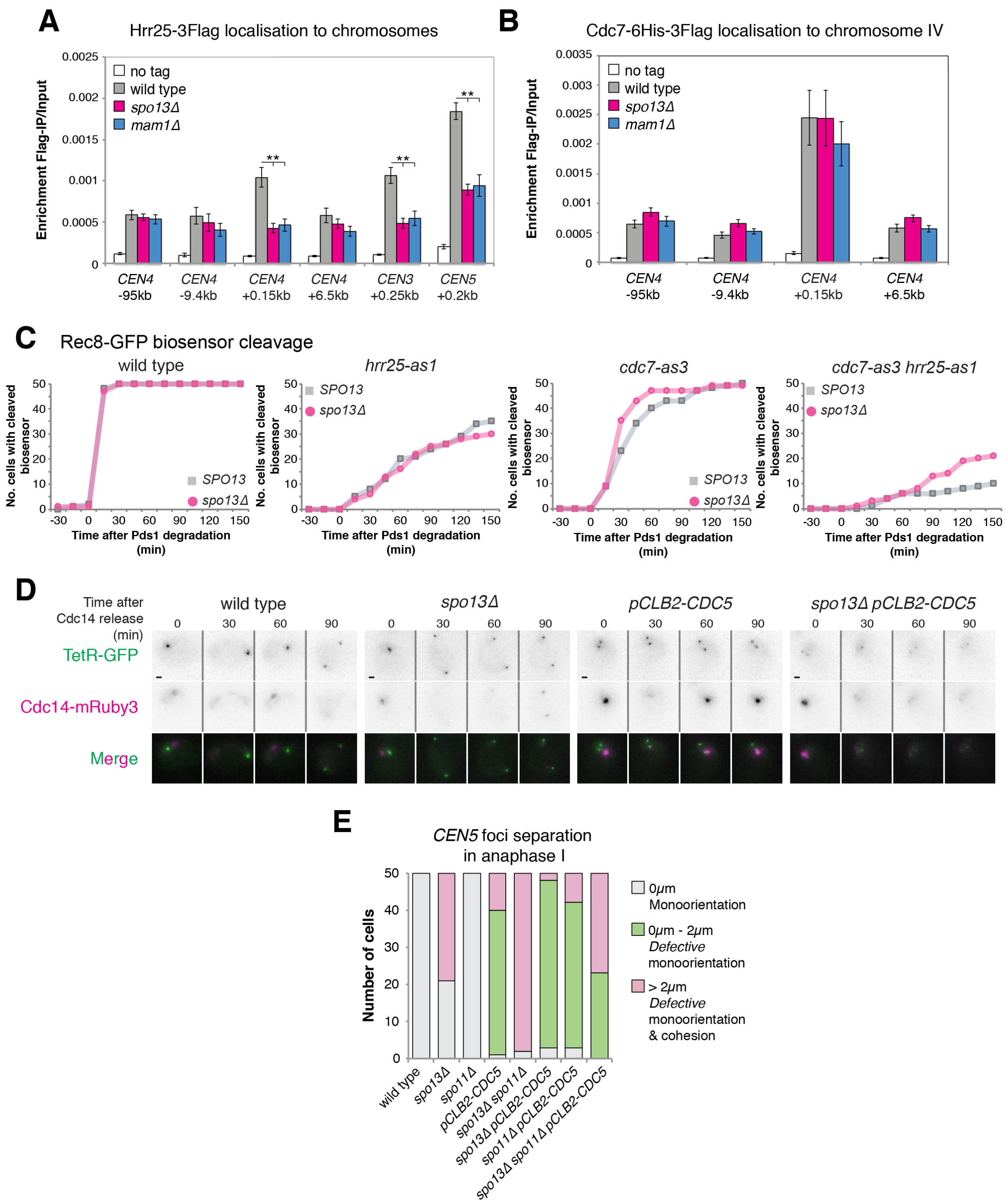

Figure S5. Spo13 represses cohesin phosphorylation, but not chromosomal association of cohesin kinases, related to Figure 6. (A-B) The association of cohesin kinases with chromosomes is not increased in *spo13Δ* mutants. (A) Chromosomal levels of Hrr25-3Flag in metaphase I were determined by anti-Flag ChIP-qPCR at the indicated sites. Mean enrichment values of Hrr25-3Flag from four experimental replicates are shown, with standard error bars (**p<0.01). (B) Chromosomal levels of Cdc7-6His-3Flag were measured by anti-Flag ChIP-qPCR. Mean enrichment values of Cdc7-6His-3Flag from four experimental replicates are shown, with standard error bars. (C) Deletion of *SPO13* enhances cleavage of a separase biosensor upon Cdc7 inhibition. Cells were scored for diffusion of the separase biosensor focus at 15 min intervals. (D-E) Sister chromatid cohesion in *spo13Δ pCLB2-CDC5* cells is partially dependent on recombination. (D) Representative images of heterozygous *CEN5*-GFP dots and *CDC14-mRuby* for indicated genotypes. (E) The maximum distance between two TetR-GFP foci was measured within two time points after the first round of Cdc14 release for 50 cells.

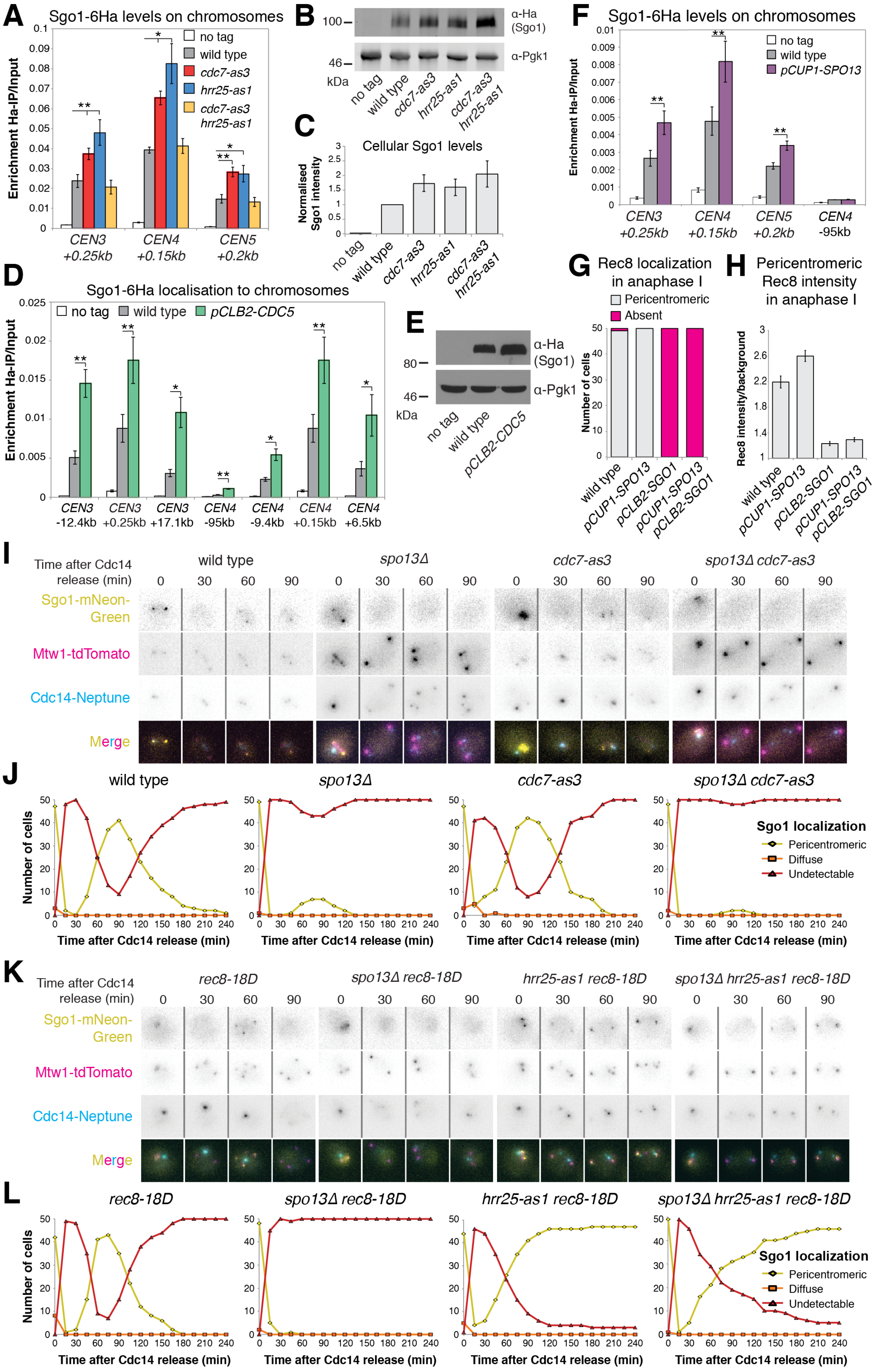

Figure S6. Chromosomal enrichment and cellular levels of Sgo1 are increased upon inhibition of single cohesin kinases, related to Figure 7. (A-C) Centromeric Sgo1 levels are increased upon inhibition of either

Cdc7 or Hrr25. (A) Chromosomal levels of Sgo1-6Ha were measured 75 min after release from a prophase I arrest by ChIP-qPCR at the indicated sites. Mean values are presented from four experimental replicates, with standard error bars (*p<0.05, **p<0.01). (B) Representative immunoblot of metaphase-I-arrested whole cell extracts as in (A) is shown with Pgk1 loading control. (C) Quantification of western blot signal. Graphs show average signal intensity relative to wild type from four biological repeats with standard error bars. (D-E) Sgo1 enrichment on chromosomes and cellular levels are increased in the absence of Cdc5. (D) Chromosomal levels of Sgo1-6Ha were measured by anti-Ha ChIP-qPCR in metaphase I at the indicated sites. Mean enrichment values of Sgo1-6Ha from four experimental replicates is shown, with standard error bars (*p<0.05, **p<0.01). (E) Immunoblot of metaphase I-arrested cultures shows slight increase in whole-cell Sgo1 levels in *pCLB2-CDC5* cells. Pgk1 serves as loading control. (F) *SPO13* overexpression increases centromeric Sgo1 levels. Mean values from four experimental repeats are shown with standard error bars (**p<0.01). (G-H) Overexpression of *SPO13* causes Sgo1-dependent overprotection of pericentromeric cohesin. (G) The presence of pericentromeric Rec8-GFP was scored in 50 anaphase I cells of the indicated genotypes from movies. (H) The intensity of Rec8-GFP was determined. (I-J) Sgo1 does not return to the pericentromere after anaphase I onset in *spo13Δ cdc7-as3* cells. (I) Representative images are shown. (J) The presence of Sgo1 was scored as pericentromeric, diffuse or absent in 50 cells for 4 h after initial Cdc14 release. (K- L) Sgo1 re-associates with the pericentromere independently of cohesin in *spo13Δ hrr25-as1* cells. (K) Representative images are shown. (L) The presence of Sgo1 was scored as pericentromeric, diffuse or absent in 50 cells for 4 h after initial Cdc14 release.

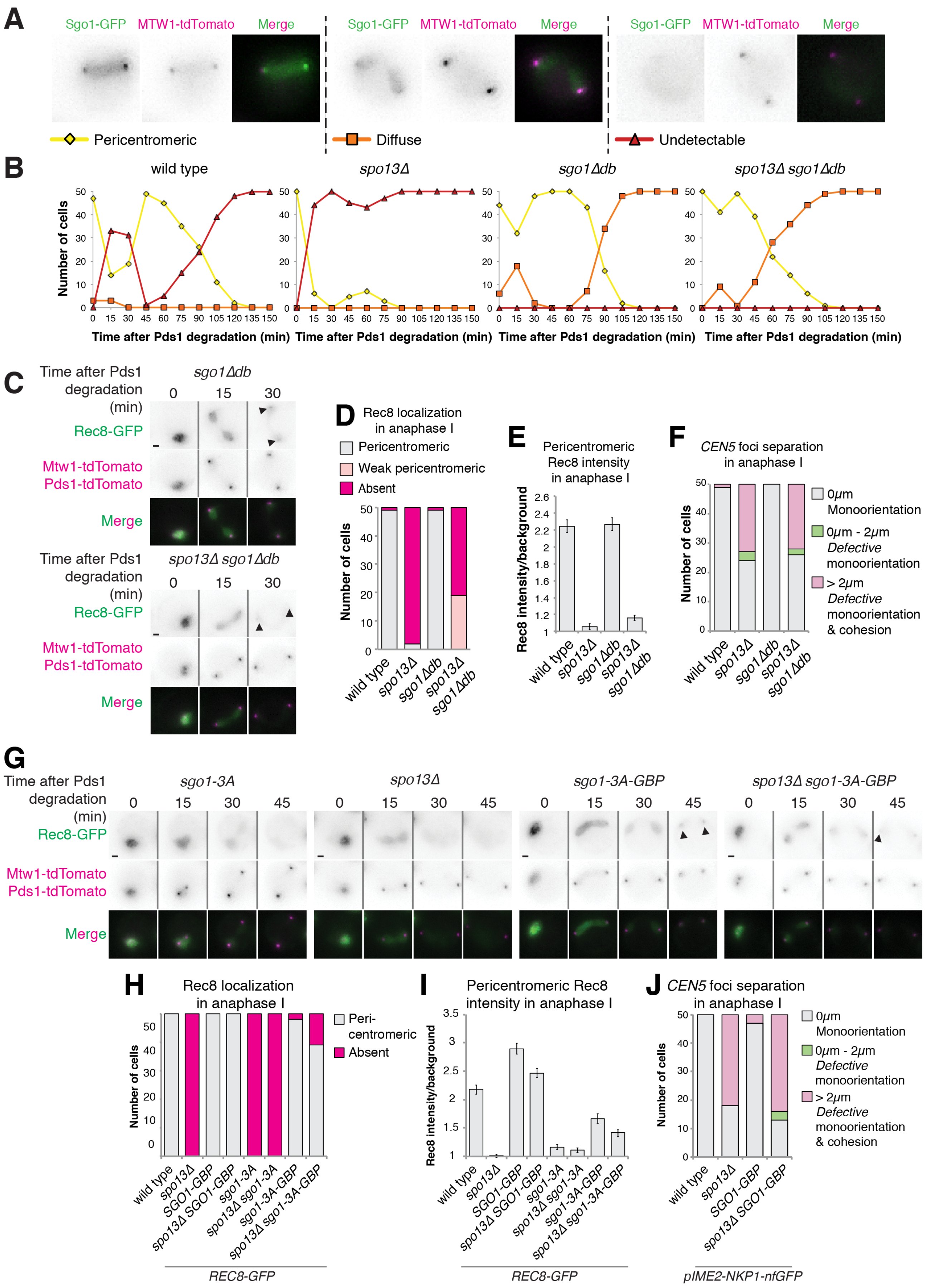

Figure S7. Chromosomal Sgo1 in *spo13Δ* anaphase I cells does not prevent sister chromatid segregation, related to Figure 7. (A-F) Preventing Sgo1 degradation does not restore sister chromatid cohesion in *spo13Δ* mutants. (A-B) Sgo1 degradation is prevented by deletion of its destruction box. (A)

Representative images of Sgo1 localization are shown in anaphase I cells. Images for pericentromeric, diffuse and undetectable localization patterns taken from *sgo1Δdb-GFP spo13Δ*, *sgo1Δdb-GFP* and *spo13Δ* cells, respectively. (B) Categories of Sgo1-GFP localization in 15 min intervals after anaphase I onset were determined from movies. (C-E) Deletion of the Sgo1 destruction box partially rescues pericentromeric cohesin in *spo13Δ* cells. (C) Representative images are shown. Scale bars represent 1µm. Arrows indicate pericentromeric cohesin. (D) The presence of pericentromeric Rec8-GFP in anaphase I was scored for 50 cells per strain. (E) Rec8-GFP intensity was measured. (F) Sister chromatid cohesion is defective in *spo13Δ sgo1Δdb* cells. *CEN5-GFP* distances were measured and scored as described in Figures 2D and 2E except that Pds1-tdTomato degradation was used as a marker for anaphase onset rather than Cdc14 release. (G-I) Tethering of Sgo1, but not Sgo1-3A to Rec8 efficiently restores pericentromeric cohesin in *spo13Δ* cells. (G) Representative live-cell images are shown. Scale bars represent 1µm. Arrows indicate pericentromeric cohesin. (H) The number of cells with pericentromeric cohesin in anaphase I is shown for the indicated genotypes after scoring 50 cells. (I) Rec8-GFP intensity was measured for 50 cells as shown in (G). Error bars represent standard error. Data for wild-type, *spo13Δ*, *SGO1-GBP* and *spo13Δ SGO1-GBP* strains shown in (H) and (I) was replicated from Figures 7D and 7E. (J) Sgo1 tethering to kinetochores does not rescue sister chromatid cohesion in *spo13Δ* cells. Cohesion functionality was determined by categorization of *CEN5-mEos* distances as described in Figures 2D and 2E.

Table S1. List of *Saccharomyces cerevisiae* strains used in this study, related to STAR methods.

Provided as an excel file.

Table S2. List of plasmids generated in this study, related to STAR methods.

Plasmid	Description	Purpose and notes
AMp1062	YIplac128-spo13-m2	*LEU2* integration plasmid carrying *spo13-m2*.
AMp1128	pFA6a-nfGFP-KanMX6	nfGFP tagging plasmid with *KanMX6* marker. nfGFP mutation is G67A. Gene also carries S65T mutation.
AMp1138	YIplac128-TetR-ymEos3.2	*LEU2* integration plasmid carrying *TetR-ymEos3.2*. Yeast codon-optimized mEos3.2 was derived from a synthetic gene construct (GeneArt).
AMp1139	pFA6a-HphMX6-pSPO21	Plasmid for promoter replacement with *pSPO21*.
AMp1147	pFA6a-GBP-His3MX6<br>*GBP* tagging plasmid with *His3MX6* marker.	GBP tagging plasmid with *His3MX6* marker.
AMp1312	pFA6a-HphMX6-pCLB2	Plasmid for promoter replacement with *pCLB2*.
AMp1351	pFA6a-mRuby3-URA3	mRuby3 tagging plasmid with *URA3* marker.
AMp1369	YIplac128-rec8-24A-GFP	*LEU2* integration plasmid carrying *rec8-24A-GFP*.
AMp1396	YIplac128-rec8-poloA-GFP	*LEU2* integration plasmid carrying *rec8-poloA-GFP*.<br>*rec8-poloA mutations are:* S136A/T173A/S179A/S197A/S199A/S215A/T249A/S285A/S386A/S387A/S410A/S421A/S465A/S466A.
AMp1398	YIplac128-rec8-poloA-3HA	*LEU2* integration plasmid carrying *rec8-poloA-3HA*.
AMp1400	pFA6a-3V5-NatMX6	3V5 tagging plasmid with *NatMX6* marker.
AMp1407	pRS303-GFP-REC8(110-531)-LacI	*HIS3* integration plasmid carrying separase biosensor corresponding to amino acids 110-531 of Rec8.
AMp1408	pRS303-GFP-rec8-24A(110-531)-LacI	*HIS3* integration plasmid carrying separase biosensor corresponding to amino acids 110-531 of rec8-24A.
AMp1409	pRS303-GFP-rec8-poloA(110-531)-LacI	*HIS3* integration plasmid carrying separase biosensor corresponding to amino acids 110-531 of rec8-poloA.
AMp1598	pFA6a-mNeonGreen-KanMX6	mNeonGreen tagging plasmid with *KanMX6* marker.
AMp1601	pFA6a-KlTRP1	Gene knockout plasmid with *Kluyveromyces lactis TRP1* marker.
AMp1603	pFA6a-mNeonGreen-KlTRP1	mNeonGreen tagging plasmid with *Kluyveromyces lactis TRP1* marker.
AMp1616	YIplac128-rec8-18D-3HA	*LEU2* integration plasmid carrying *rec8-18D-3HA*.

Plasmid	Description	Purpose and notes
AMp1620	pSPO21-HRR25-nfGFP	*URA3* integration plasmid carrying *HRR25* under control of the *SPO21* promoter and tagged with non-fluorescent GFP.

Table S3. qPCR primers used in this study, related to STAR methods.

For distances from centromeres, "-" indicates the location is upstream of the centromere, whereas "+" indicates the location is downstream of the centromere.

**Chr.**	**Location**	**Distance from centromere**	**Primer pair**	**Sequence**
I	Centromere	+0.02kb	8162	CGGAGCTTTCATTTCAAGCG
			8163	CTAATAAGGTCCACCGCCTA
III	Centromere	-0.05kb	8166	CATATAAACCGAACCCTTCCC
			8167	CCATATTGTTTGGCGCTGAT
III	Centromere	+0.25kb	1279	TGTTGATGGGTTTACAATTT
			1280	CTTTCAATGATTGCTCTAAATC
III	Arm	+103kb	1285	ATGGTACCTAGCTCGTGAAT
			1286	GGATTTGTCAACTTGGAACT
III	Peri-centromere	-12.4kb	4867	ATAGCAACGACGTAAGAGGT
			4868	GCCGAACTTTCTTTCCATA
III	Peri-centromere	+17.1kb	4875	TTAGCGCAGATTCAACAATA
			4876	TAGGCTTGTCATTTGTCCTA
IV	Arm	-95kb	782	AGATGAAACTCAGGCTACCA
			783	TGCAACATCGTTAGTTCTTG
IV	Centromere	+0.15kb	794	CCGAGGCTTTCATAGCTTA
			795	ACCGGAAGGAAGAATAAGAA
IV	Peri-centromere	-10.5kb	910	ACAAAGGATGATTTGTCAGG
			911	CTCTCTCCTTGGCTTGTTTA
IV	Peri-centromere	-6.5kb	1323	TAAGTCAGGACGTATTGGTG
			1324	GAGCCTTCCATAACTTCAAC
IV	Peri-centromere	-5.6kb	1327	AGATCCCTGTAGACTGGATG
			1328	ACGCGTTTGAAGAACATT
IV	Peri-centromere	-3kb	1331	TTTATCATCATCCGCATCTA
			1332	AACGAACAAGATAGGAAACC
IV	Peri-centromere	-2.2kb	1333	TGAAATCCTTCCTTTCTCAG
			1334	AATCGCATTATCAAACAGAA
IV	Peri-centromere	+10.7kb	1337	CGATAGTATTGATTGTGGGA
			1338	CCAGGAAATGCTTCTAACTT
IV	Peri-centromere	-9.4kb	4877	TACAGCAAATGTTGGTGATT
			4878	ACCTGCTTGTTCAACTCTCT
IV	Peri-centromere	+6.5kb	4885	AGAAACCACCCATAATTGAG
			4886	ACGATAGTCAAATTTCCGTT
V	Centromere	+0.05kb	8168	AAGTCTTGTTTAGTGCAAGCC
			8169	ACCGCATTTCCTTGATTTACTG
V	Centromere	+0.2kb	945	TGAAGGTGAGCTTAAGACAG
			946	CAACCATGTTCGTAGCTAAA
V	Arm	-84kb	949	CTACGGTAAATCTGGGTAGG
			950	TCCACTATCAAGTCACCAGA
