# Supplementary material for: Reductional Meiosis I Chromosome Segregation Is Established by Coordination of Key Meiotic Kinases
Source: Dev Cell. 2019 May 20;49(4):526–541.e5. doi: 10.1016/j.devcel.2019.04.003 (PMC6547162; doi:10.1016/j.devcel.2019.04.003)
Supplement: Document S2. Article plus Supplemental Information [file mmc3.pdf]

Article

Developmental Cell

Reductional Meiosis I Chromosome Segregation Is Established by Coordination of Key Meiotic Kinases

Graphical Abstract

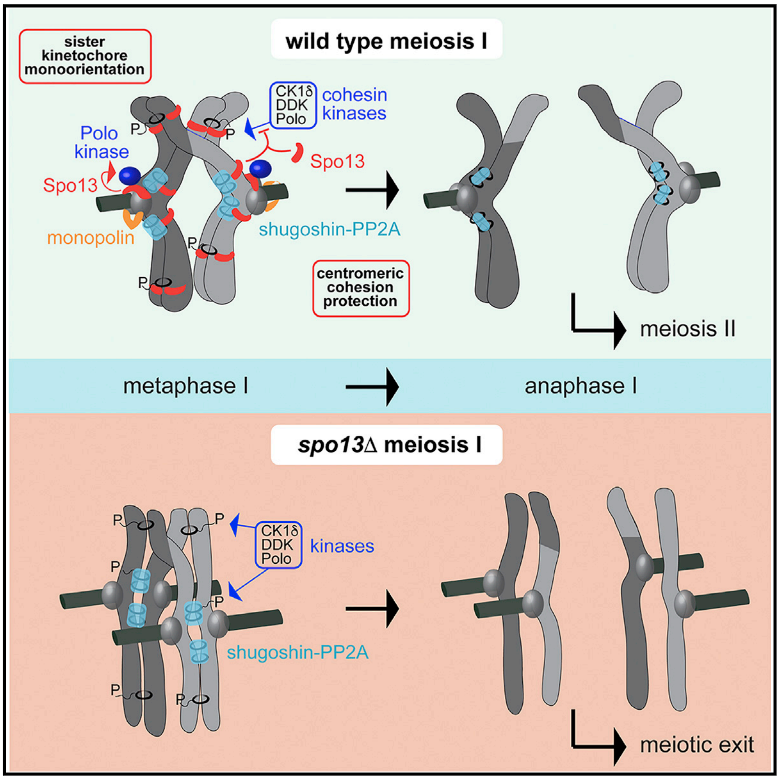

Highlights

- Spo13 recruits Polo kinase to kinetochores to direct sister chromatid co-segregation
- Kinetochore-associated Polo drives co-segregation independently of monopolin
- Spo13 counteracts cohesin kinases to prevent premature loss of centromeric cohesion
- Spo13 restricts CK1δ to allow shugoshin reaccumulation after meiosis I

Authors

Stefan Galander, Rachael E. Barton, Weronika E. Borek, ..., Daniel Robertson, Juri Rappsilber, Adèle L. Marston

Correspondence

adele.marston@ed.ac.uk

In Brief

Segregation of homologs—rather than sister chromatids—is unique to meiosis I. Galander et al. show that the meiosis-I-specific Spo13 protein prevents sister chromatid segregation by controlling the effects of multiple kinases to both enforce sister kinetochore co-orientation and prevent premature loss of cohesion.

Galander et al., 2019, Developmental Cell *49*, 526–541
May 20, 2019 © 2019 The Author(s). Published by Elsevier Inc.
https://doi.org/10.1016/j.devcel.2019.04.003

CellPress

Developmental Cell
Article

Reductional Meiosis I Chromosome Segregation Is Established by Coordination of Key Meiotic Kinases

Stefan Galander,[1] Rachael E. Barton,[1,3] Weronika E. Borek,[1,3] Christos Spanos,[1] David A. Kelly,[1] Daniel Robertson,[1] Juri Rappsilber,[1,2] and Adèle L. Marston[1,4,*]
[1]The Wellcome Centre for Cell Biology, Institute of Cell Biology, School of Biological Sciences, Michael Swann Building, Max Born Crescent, Edinburgh EH9 3BF, UK
[2]Institute of Biotechnology, Technische Universität Berlin, Berlin, Germany
[3]These authors contributed equally
[4]Lead Contact
*Correspondence: adele.marston@ed.ac.uk
https://doi.org/10.1016/j.devcel.2019.04.003

SUMMARY

Meiosis produces gametes through a specialized, two-step cell division, which is highly error prone in humans. Reductional meiosis I, where maternal and paternal chromosomes (homologs) segregate, is followed by equational meiosis II, where sister chromatids separate. Uniquely during meiosis I, sister kinetochores are monooriented and pericentromeric cohesin is protected. Here, we demonstrate that these key adaptations for reductional chromosome segregation are achieved through separable control of multiple kinases by the meiosis-I-specific budding yeast Spo13 protein. Recruitment of Polo kinase to kinetochores directs monoorientation, while independently, cohesin protection is achieved by containing the effects of cohesin kinases. Therefore, reductional chromosome segregation, the defining feature of meiosis, is established by multifaceted kinase control by a master regulator. The recent identification of Spo13 orthologs, fission yeast Moa1 and mouse MEIKIN, suggests that kinase coordination by a meiosis I regulator may be a general feature in the establishment of reductional chromosome segregation.

INTRODUCTION

Unlike mitosis, meiosis requires two rounds of chromosome segregation without intervening DNA replication. Meiosis I is distinctive because homologs, rather than sister chromatids, are segregated, requiring adaptations to the chromosome segregation machinery (Marston, 2014). Firstly, homologous chromosomes recombine to create linkages (chiasmata) that bias their stable attachment to microtubules from opposite spindle poles. Secondly, sister kinetochores monoorient, meaning that they face the same, rather than opposite, spindle poles. Lastly, cohesin, which holds sister chromatids together, is cleaved in two steps. During meiosis I, cohesin cleavage on chromosome arms allows homolog segregation, but cohesin protection in the region around centromeres (called pericentromeres) holds sister chromatids together until meiosis II.

Cohesin comprises three core subunits, Smc1, Smc3, and Scc1, and accessory subunits Scc3 and Pds5 (Marston, 2014). Upon proper attachment of all chromosomes to the spindle, securin (Pds1) is destroyed, liberating separase (Esp1), which cleaves Scc1, triggering chromosome segregation. During meiosis, Rec8 replaces Scc1 (Buonomo et al., 2000; Watanabe and Nurse, 1999) and its cleavage by separase during both anaphase I and II requires prior Rec8 phosphorylation. In budding yeast, three kinases phosphorylate Rec8: CK1δ (Hrr25), Dbf4-dependent kinase (DDK) Cdc7 (Katis et al., 2010), and Polo kinase (Cdc5) (Brar et al., 2006), although the contribution of Cdc5 to cohesin cleavage is under debate (Katis et al., 2010; Attner et al., 2013). During meiosis I, shugoshin (Sgo1) recruits protein phosphatase 2A (PP2A) to the pericentromere to counteract this phosphorylation and prevent Rec8 cleavage (Katis et al., 2004a; Kitajima et al., 2004, 2006; Lee et al., 2008; Marston et al., 2004; Riedel et al., 2006; Tang et al., 2006). Rec8 deprotection in anaphase II requires Hrr25-dependent cohesin phosphorylation and Sgo1 inactivation (Argüello-Miranda et al., 2017; Jonak et al., 2017).

Cohesin also promotes sister kinetochore monoorientation in fission yeast, *A. thaliana*, and *C. elegans*, but not budding yeast (Chelysheva et al., 2005; Monje-Casas et al., 2007; Severson et al., 2009). In fission yeast, Rec8-containing cohesin is thought to juxtapose sister centromeres to create a geometry that favors sister kinetochore monoorientation (Sakuno et al., 2009; Yokobayashi et al., 2003). In contrast, in budding yeast, a dedicated complex called monopolin directs sister kinetochore monoorientation. Monopolin consists of the meiosis-specific Mam1 protein (Tóth et al., 2000), the nucleolar proteins Lrs4 and Csm1 (Rabitsch et al., 2003), and Hrr25 (Petronczki et al., 2006), which together form a V-shaped structure thought to fuse sister kinetochores to form a common microtubule attachment surface (Corbett et al., 2010; Sarangapani et al., 2014).

Re-programming of the chromosome segregation machinery to segregate homologs requires synchronized establishment of sister kinetochore monoorientation and cohesin protection. This predicts the existence of a master regulator that can drive these two adaptations, essentially converting mitosis into meiosis. An attractive candidate is the budding yeast meiosis-I-specific Spo13 protein. Cells lacking *SPO13* undergo

 © 2019 The Author(s). Published by Elsevier Inc.
This is an open access article under the CC BY license (http://creativecommons.org/licenses/by/4.0/).

a single meiotic division, show monoorientation defects, and fail to protect cohesin (Katis et al., 2004b; Klapholz and Esposito, 1980; Lee et al., 2004; Shonn et al., 2002; Galander et al., 2019). Accordingly, Spo13 is required for monopolin localization at kinetochores (Katis et al., 2004b; Lee et al., 2004) and is implicated in ensuring the proper pericentromeric localization of Sgo1 (Kiburz et al., 2005). Functionally orthologous fission yeast Moa1 and mouse MEIKIN are similarly present only in meiosis I (Kim et al., 2015). All three proteins bind Polo kinase and its recruitment to centromeres by fission yeast Moa1 and mouse MEIKIN has been suggested to facilitate monoorientation and cohesin protection (Kim et al., 2015; Matos et al., 2008; Miyazaki et al., 2017).

Here, we reveal how budding yeast Spo13 directs both sister kinetochore monoorientation and cohesin protection to define the meiotic chromosome segregation pattern. We show that recruitment of Polo kinase Cdc5 to kinetochores by Spo13 is critical for monoorientation but not cohesin protection. Instead, Spo13 protects cohesin by restricting the effects of the cohesin kinases Hrr25 and DDK, thereby both limiting cohesin phosphorylation and promoting retention of the Sgo1 cohesin protector. Overall, Spo13 orchestrates coordinated temporal and spatial control on key meiotic kinases to establish the meiotic segregation pattern.

RESULTS

Spo13 Recruits Cdc5 to Centromeres

To test if Spo13, like Moa1 and MEIKIN, recruits Polo kinase to centromeres, we analyzed chromosomal Cdc5 by chromatin immunoprecipitation and qPCR (ChIP-qPCR). Cdc5 enrichment at centromeric, but not pericentromeric or arm sites, was significantly reduced in *spo13Δ* metaphase-I-arrested cells and in the *spo13-m2* mutant, which is deficient in binding Cdc5 (Matos et al., 2008) (Figure 1A). Cellular Cdc5 levels (Figure S1A) and metaphase I arrest efficiency (Figure S1B), known to be less robust in *spo13Δ* cells (Katis et al., 2004b), were comparable. Reduced centromeric Cdc5 was also not an indirect consequence of the loss of monoorientation in *spo13Δ* and *spo13-m2* cells because Cdc5 and Spo13 associate with centromeres normally in the absence of the monopolin component Mam1 (Figures S1C and S1D).

Consistently, overexpression of *SPO13*, but not *spo13-m2*, from the copper-responsive *CUP1* promoter increased Cdc5, though not Spo13, levels at centromeres (Figures 1B and 1C). Both Spo13 and Cdc5 levels were increased at a chromosomal arm site and cellular Cdc5 levels were also modestly elevated upon *SPO13* overexpression (Figure S1E), suggesting that stabilization of Cdc5 enhances its chromosomal association. However, less Spo13-m2 associated with centromeres, compared to Spo13, even when over-produced (Figures 1C and S1E), suggesting co-dependence of Spo13 and Cdc5 for their centromeric localization. We conclude that centromeric enrichment of Cdc5 depends on its association with Spo13.

Spo13 Associates with Kinetochores and Cohesin-Rich Sites

Spo13 accumulates throughout the nucleus prior to metaphase I and is also found associated with chromosomes at centromeres and cohesin arm sites before being degraded in anaphase I (Figure S1F; Katis et al., 2004b; Sullivan and Morgan, 2007). To determine Spo13 dependence on cohesin (Rec8), we performed calibrated Spo13-3Flag ChIP followed by sequencing (ChIP-seq) in prophase-arrested cells. Total cellular levels of Spo13 and the most prominent Spo13 peaks at centromeres were independent of Rec8 (Figures 1D, 1E, S1G, and S1H). Smaller, Rec8-dependent Spo13 peaks were found at non-centromeric sites occupied by Rec8. Average Spo13 signal around all centromeres was narrower in *rec8Δ* cells than wild type (Figure 1E) and the difference of the profiles around *CEN1* (Figure 1D) revealed a bimodal peak, reminiscent of the Rec8 peak. Therefore, Spo13 localization to chromosome arms and pericentromeres depends on cohesin, while Spo13 association with centromeres is cohesin independent.

Kinetochore-Bound Cdc5 Is Sufficient for Monoorientation

To determine whether kinetochore recruitment of Cdc5 underlies Spo13 function in monoorientation and cohesin protection, we artificially targeted Cdc5 to kinetochores (Cdc5-Kt; Figures 2A and S2A). To assay kinetochore monoorientation, we visualized heterozygous *CEN5*-tdTomato foci, which split into two distinct foci during metaphase I if monoorientation is defective. While rarely observed in wild-type cells (<1%), split *CEN5*-tdTomato foci are observed in approximately 30% of *spo13Δ* metaphase-I-arrested cells. Remarkably, Cdc5-Kt reduced this fraction by more than half (Figure 2B). Lrs4 hyperphosphorylation, which correlates with monopolin localization to kinetochores and is lost in the absence of *SPO13* and in *spo13-m2* (Matos et al., 2008), was also partially rescued by Cdc5-Kt in *spo13Δ* cells (Figure 2C). Therefore, Spo13-mediated recruitment of Cdc5 to kinetochores promotes Lrs4 phosphorylation and sister-kinetochore monoorientation.

To further assay the effect of Cdc5-Kt, we established a live-cell sister kinetochore monoorientation and cohesin protection assay. We followed heterozygous fluorophore-labeled *CEN5* foci through meiosis I. Depending on whether or not monoorientation is established and/or pericentromeric cohesin is retained, three different scenarios result when cells enter anaphase I (Figure 2D). First, in wild-type cells, a single *CEN5* focus segregates to one of the spindle poles. Second, if monoorientation is lost, split *CEN5* foci remain in close proximity (<2 μm) because protected pericentromeric cohesin holds sister chromatids together. Third, in cells defective for both monoorientation and sister chromatid cohesion, *CEN5* foci separate over a greater distance (>2 μm). We categorized cells based on the distance between *CEN5* foci in anaphase I (as assessed by release of Cdc14 from the nucleolus or by Pds1 disappearance; Figure 2D). The majority (~64%) of *spo13Δ* cells fail to co-segregate *CEN5* foci during anaphase I, but remarkably Cdc5-Kt (Figure 2E), though not kinase-defective Cdc5^{N209A}-Kt (Figure S2B), almost completely restored sister chromatid co-segregation. Cdc5-Kt also suppressed the high frequency of sister kinetochore biorientation in *spo13Δ spo11Δ* cells caused by the absence of homolog-linking chiasmata (Figure 2E). Hence, enrichment of Cdc5 kinase at kinetochores is a crucial function of Spo13 in monoorientation.

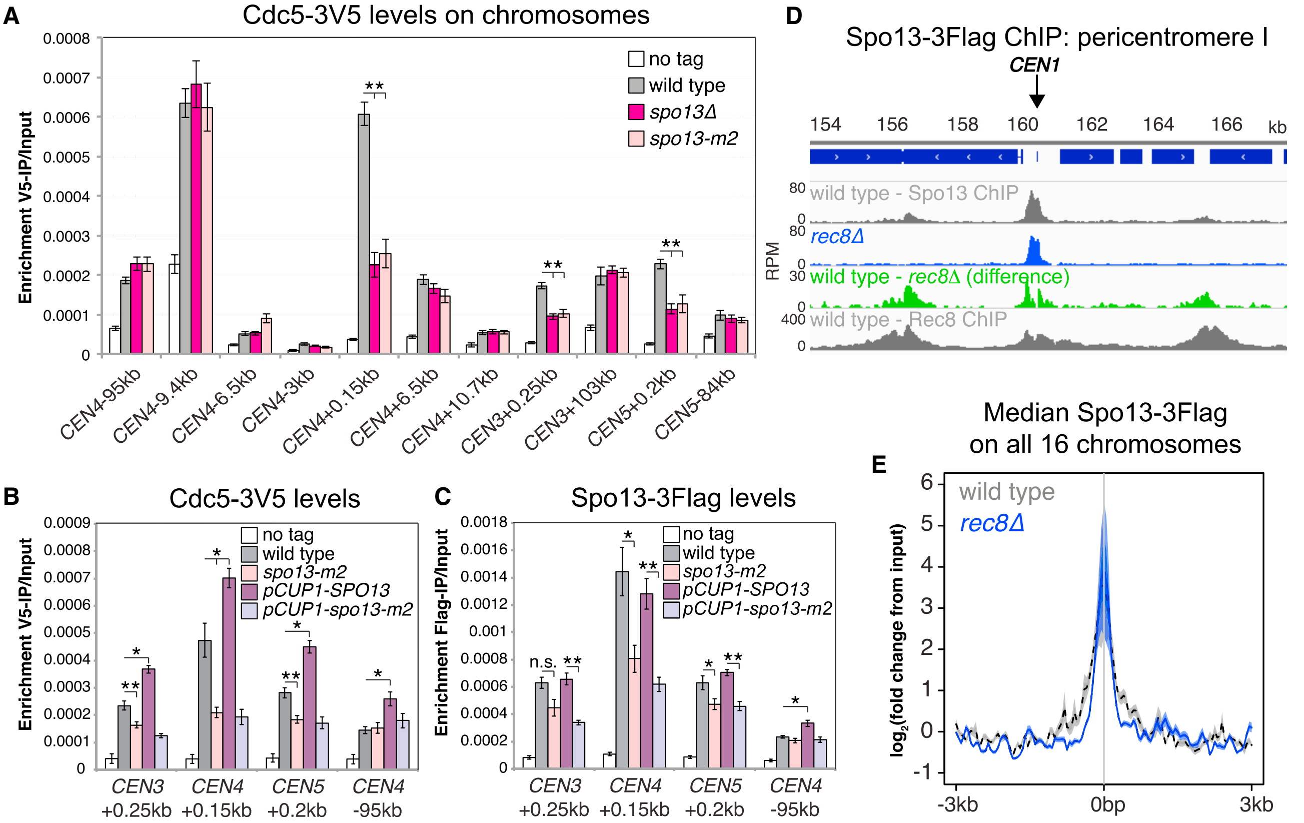

Figure 1. Cdc5 Localization to Centromeres Depends on Spo13

(A) Enrichment of Cdc5-3V5 during metaphase I.

(B and C) Effect of *SPO13* overexpression on Cdc5-3V5 (B) and Spo13-3Flag (C) enrichment in metaphase I.

(A–C) Mean ChIP-qPCR values from four biological replicates with standard error bars (n.s., not significant, *p < 0.05, **p < 0.01).

(D and E) Spo13-3Flag ChIP-seq and Rec8-3Ha ChIP-seq from prophase-arrested wild-type and *rec8Δ* cells. (D) Close-up of *CEN1* pericentromere. (E) Median Spo13-3Flag signal averaged within a 6 kb region surrounding the centromere.

See also Figure S1.

Cdc5-Kt Rescues Sister Chromatid Co-segregation Independently of Monopolin

Partial Lrs4 hyperphosphorylation in *CDC5-Kt spo13Δ* cells (Figure 2C) suggested that Cdc5-Kt may promote monoorientation by restoring recruitment of monopolin, which requires Spo13 for its localization at kinetochores (Katis et al., 2004b; Lee et al., 2004). Surprisingly, although Cdc5-Kt enhanced centromeric Mam1 recruitment, it did so only in the presence of *SPO13* (Figure 2F), suggesting that Cdc5-Kt promotes monoorientation independently of monopolin recruitment to kinetochores. Indeed, remarkably, Cdc5-Kt did not require the Mam1, Lrs4, or Csm1 components of monopolin to promote monoorientation in metaphase-I-arrested cells (Figures S2C–S2E) or for sister chromatid co-segregation in anaphase I (Figures 2G, S2F, and S2G), regardless of the presence of Spo13. Therefore, Spo13-dependent recruitment of Cdc5 to kinetochores directs sister chromatid co-segregation through a mechanism independent of monopolin-dependent sister kinetochore crosslinking.

We asked if these effects were specific to Cdc5 by tethering Hrr25 (Figure S2H), a kinase component of monopolin that is recruited to kinetochores by Mam1 (Petronczki et al., 2006). While Hrr25-Kt did not prevent sister *CEN5* foci separating at metaphase I (Figure S2I), it partially restored the co-segregation of sister chromatids in anaphase I in *mam1Δ* and *mam1Δ spo13Δ*, but not *spo13Δ* cells (Figure S2J), confirming the functionality of Hrr25-Kt. This suggests that Hrr25 may play its most critical role in monoorientation during anaphase I and demonstrates the specific requirement for Cdc5 recruitment to kinetochores by Spo13.

We also tested the effect of Cdc5-Kt on the segregation of homologs to opposite poles during meiosis I. Imaging homozygous *CEN5*-tdTomato foci revealed homolog co-segregation in a fraction (~18%) of Cdc5-Kt cells, which increased to ~50% in the absence of Mam1 and/or Spo13 (Figure 2H). Therefore, while Cdc5-Kt nearly always (>95%) directs sister kinetochore co-segregation during meiosis I, even in the absence of monopolin (Figure 2G), the effect on homolog co-segregation is more modest and, interestingly, is suppressed by the presence of monopolin. Although the reasons for these observations remain unclear, forcing Cdc5 to kinetochores in an unregulated manner may enhance its effect, such that not only sister kinetochores but also homologous kinetochores are co-oriented. We conclude that monopolin and kinetochore-associated Cdc5 play distinct roles in ensuring proper kinetochore orientation during meiosis I.

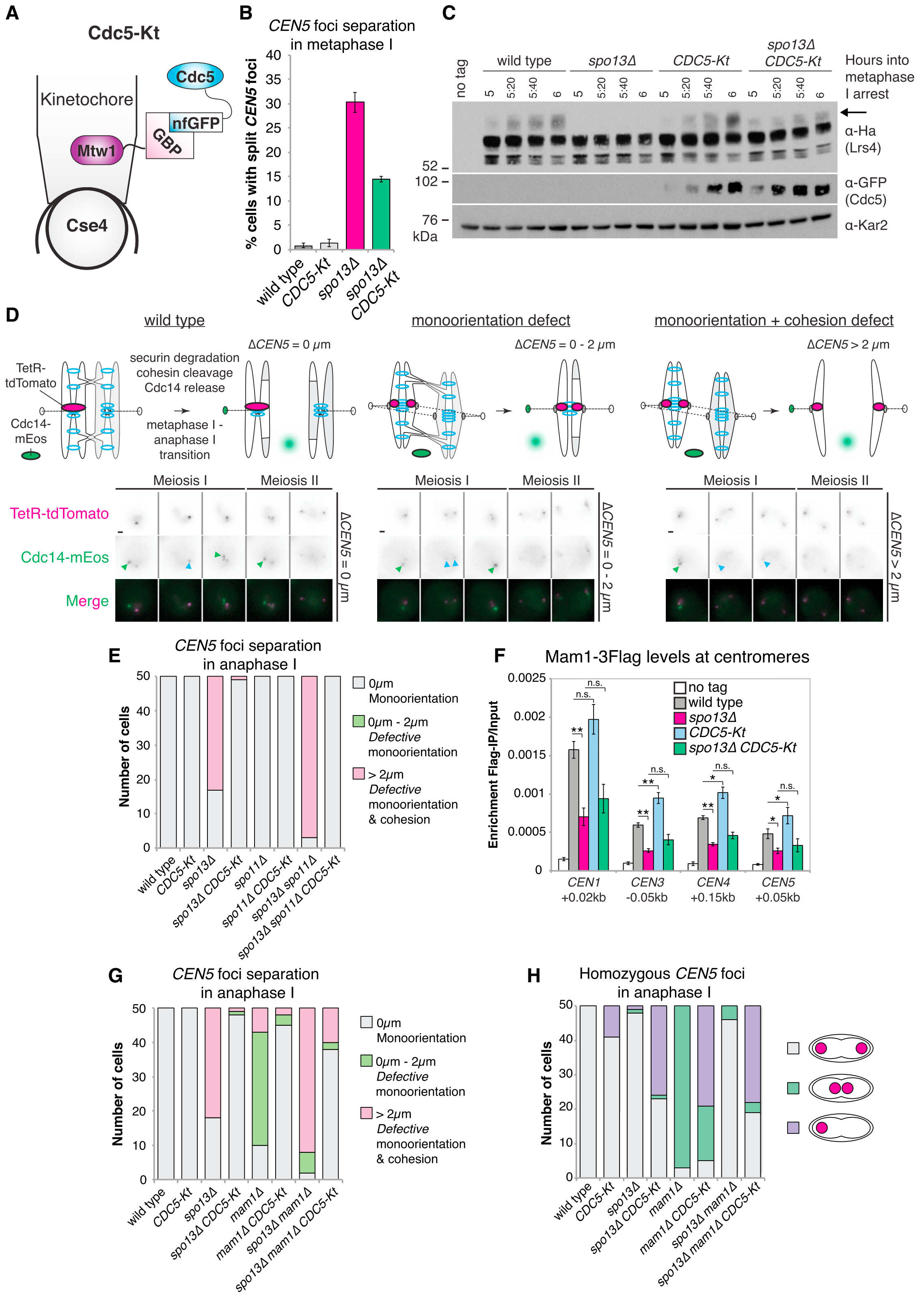

Figure 2. Cdc5-Kt Restores Sister Chromatid Co-segregation to Monoorientation Mutants

(A) Schematic representation of Cdc5-Kt.

(B) Mean number of metaphase-I-arrested cells with two distinct tdTomato foci from three experimental replicates with standard error bars (n = 200).

(legend continued on next page)

Cdc5-Kt Promotes Pericentromeric Cohesin Retention but Is Insufficient for Sister Chromatid Cohesion

To test whether centromeric Cdc5 is required for cohesin protection, we asked whether *spo13-m2* would permit sister chromatid segregation during meiosis I in *mam1Δ* mutants. Because of the loss of monoorientation, *mam1Δ* cells biorient sister kinetochores in meiosis I, but sister chromatid segregation is prevented because centromeric cohesion persists (Tóth et al., 2000). In contrast to *spo13Δ mam1Δ* cells, which separate sister chromatids in anaphase I (Figure 2G), *spo13-m2 mam1Δ* double mutants largely retain sister chromatid cohesion, similar to *mam1Δ* single mutants (Figure S3A). However, unlike *spo13Δ*, *spo13-m2* cells show only minor monoorientation defects (Figure S3A), and residual Spo13-Cdc5 interaction in this mutant (Matos et al., 2008) means a potential role for kinetochore-recruited Cdc5 in cohesion protection could not be ruled out.

As an alternative approach, we asked whether Cdc5-Kt allows retention of pericentromeric cohesin in anaphase I *spo13Δ* cells by imaging Rec8-mNeonGreen (mNG). Faint Rec8-mNG foci that co-localize with the kinetochore marker Dsn1-tdTomato persist in wild-type anaphase I cells, but not in *spo13Δ* cells, indicating a failure to protect cohesin (Figures S3B and S3C). However, Cdc5-Kt restored pericentromeric Rec8 foci in 52% of anaphase I *spo13Δ* cells (Figures S3B and S3C) and increased pericentromeric Rec8 intensity in wild-type and *spo13Δ* backgrounds (Figure S3D). The rescue of the monoorientation defect of *spo13Δ* cells by Cdc5-Kt (Figure 2E) precludes assaying cohesion directly in the live-cell assay (Figure 2D). Instead, centromeric cohesion functionality can be inferred by scoring *CEN5* foci separation after anaphase I (Figures S3E and S3F). Wild-type and *pCLB2-SGO1* controls split heterozygous *CEN5* foci soon after the first round of Cdc14 release in response to meiosis II spindle tension (Figures S3E and S3F). We also observed split *CEN5* foci in 78% of *spo13Δ* cells (Figures S3E and S3F). Because meiosis II spindles do not form in *spo13Δ* cells (Figure S3G), this post-anaphase I *CEN5* foci separation must be the result of cohesion defects, rather than spindle tension. Cdc5-Kt neither delayed the appearance of split foci nor reduced their frequency (88%) in the absence of *SPO13* (Figure S3F). Therefore, although Cdc5-Kt increases pericentromeric Rec8-mNG in anaphase I *spo13Δ* cells, cohesion defects persist. We conclude that Spo13 functions other than Cdc5 recruitment to kinetochores are important for cohesion protection.

Cohesin-Associated Cdc5 Promotes Cohesion Loss

Paradoxically, Cdc5-dependent phosphorylation of Rec8 was reported to be important not for its protection but for its cleavage (Attner et al., 2013; Brar et al., 2006). Indeed, in Cdc5-depleted cells, the slower migrating forms of Rec8 that appear after prophase I exit in wild-type cells are largely absent (Figure 3A) and the fastest migrating form (Figure 3A, arrowheads), which likely corresponds to unphosphorylated, protected Rec8 at pericentromeres persists. Consistent with this interpretation, the fastest migrating, presumed protected, Rec8 species rapidly disappears in cells depleted of the cohesin-protector Sgo1, even when Cdc5 is also depleted (Figure 3B, arrowheads). Thus, Cdc5 may not be essential for cohesin protection.

To directly determine the effect of Cdc5-directed Rec8 phosphorylation on cohesin retention and sister chromatid cohesion, we tethered Cdc5-GBP to Rec8-GFP (henceforth Cdc5-Coh). Cohesin loading prior to prophase I is comparable in Cdc5-Coh and wild-type cells (Figures S3H and S3I). However, upon nuclear division at anaphase I, distinct pericentromeric Rec8-GFP foci were absent from Cdc5-Coh cells. Instead, diffuse nuclear signal was observed, even in the absence of *SPO13* (Figures 3C and 3D), which likely represents cleaved Rec8-GFP fragments bound to nuclear Cdc5-GBP rather than cohesin conferring sister chromatid cohesion, since nuclear division occurs. Consistently, kinetochore-proximal Rec8 intensity in Cdc5-Coh cells was lower than in wild type but higher than in *spo13Δ* cells (Figure 3E). Therefore, Cdc5-Coh promotes cohesin removal in anaphase I, a conclusion reinforced by analysis of *CEN5* foci segregation: while Cdc5-Coh had no impact in a wild-type background, it caused *CEN5* foci to segregate to opposite poles in virtually all *spo13Δ* cells (Figure 3F). Although it is unclear why Cdc5-Coh enhances sister chromatid biorientation in *spo13Δ* cells, this confirms that cohesin-associated Cdc5 does not universally protect Rec8 and indicates a specific role for kinetochore-bound Cdc5 in promoting sister kinetochore monoorientation. Cdc5-Coh also permitted *mam1Δ* cells to segregate sister chromatids to opposite poles in anaphase I, providing further evidence that Cdc5 promotes cohesion loss (Figure 3G). Therefore, in contrast to kinetochore-bound Cdc5, which promotes retention of, albeit non-functional, centromeric cohesin, cohesin-bound Cdc5 promotes cohesion dissolution.

Sgo1-PP2A Localizes to Pericentromeres in Metaphase I Independently of Spo13

Since Cdc5-Kt only modestly increased pericentromeric cohesin in anaphase I *spo13Δ* cells and was insufficient for functional cohesion, Spo13 must protect cohesion through other mechanisms. Spo13 may promote localization of the Sgo1-PP2A cohesin protector at pericentromeres (Kiburz et al., 2005), though earlier reports found no impairment of Sgo1 localization in *spo13Δ* cells (Katis et al., 2004b; Lee et al., 2004). Consistently,

(C) Immunoblot analysis of Lrs4-6Ha, Cdc5-GFP, and Kar2 loading control. Arrow indicates hyperphosphorylated Lrs4-6Ha.

(D) Assay for monoorientation and cohesion defects with representative images below. Scale bars, 1 μm. Green arrows, nucleolar Cdc14; Cyan arrows, Cdc14 at SPBs in anaphase I.

(E) Frequency of heterozygous *CEN5* distance categories from (D) after live-cell imaging. Maximum distance between two TetR-tdTomato foci within two time points after initial Cdc14 release was measured (n = 50).

(F) Mean enrichment of Mam1-3Flag in metaphase I from six experimental replicates, with standard error bars (*p < 0.05, **p < 0.01).

(G) *CEN5* distance categories determined as in Figure 2E.

(H) Frequency of homozygous *CEN5*-tdTomato foci segregating (gray), co-segregating (purple), or failing to segregate (green) within two time points after the first round of Cdc14 release (n = 50).

See also Figure S2.

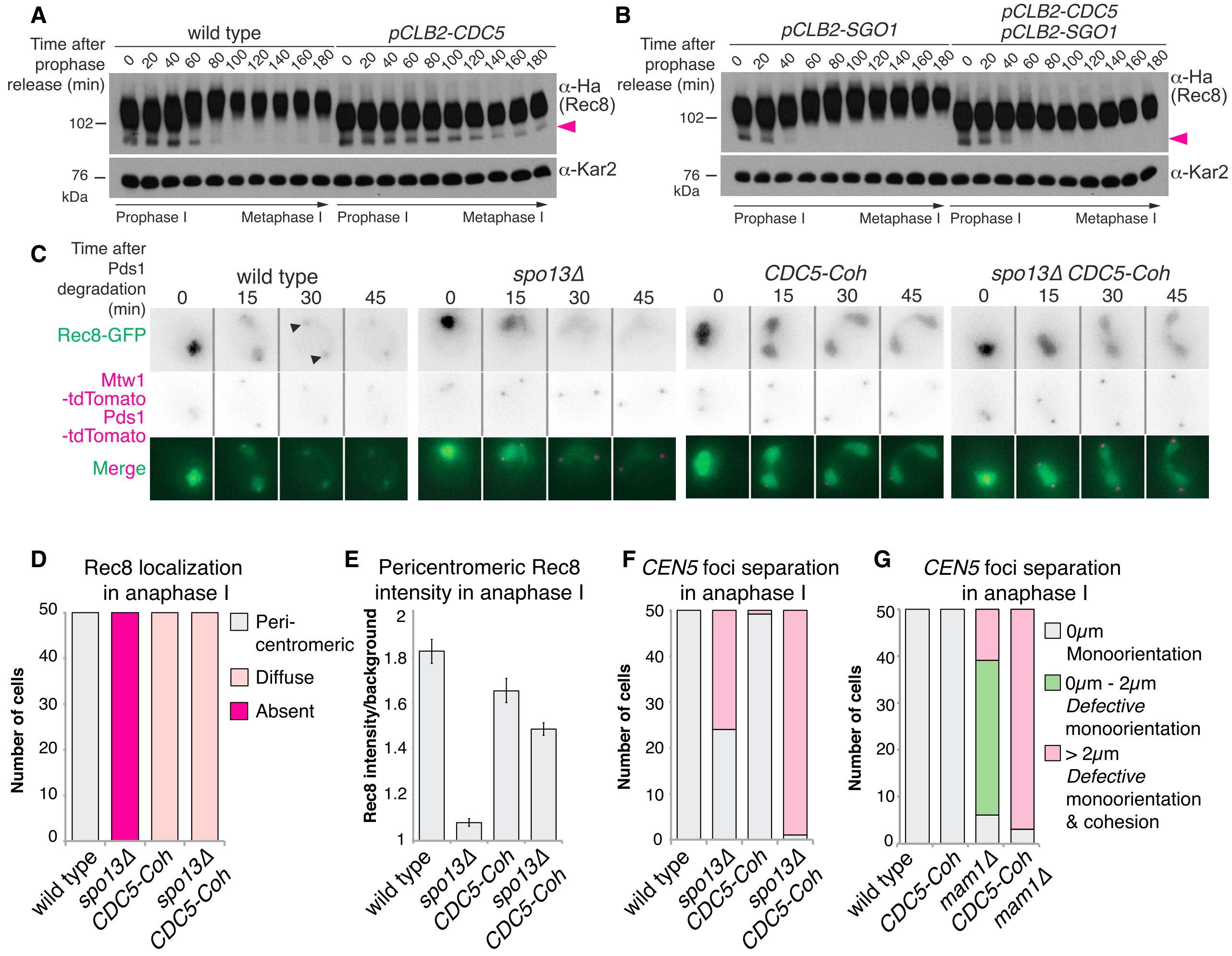

Figure 3. Cohesin-Associated Cdc5 Promotes Cohesion Loss

(A and B) Immunoblot analysis of Rec8-3Ha and Kar2 loading control for wild-type (left) and Cdc5-depleted (right) cells (A) or for cells depleted of either Sgo1 alone (left) or both Sgo1 and Cdc5 (right) (B). Cells were released from prophase into a metaphase I arrest. Arrowhead indicates unphosphorylated Rec8.

(C–E) Time-lapse series of Rec8-GFP in Cdc5-Coh cells. (C) Representative images, arrows indicate pericentromeric cohesin. (D) Scoring for presence or (E) average intensity of pericentromeric Rec8-GFP in anaphase I with standard error bars (n = 50).

(F and G) Heterozygous *CEN5* distance categories as in Figure 2E for *spo13Δ* (F) or *mam1Δ* (G) cells carrying Cdc5-Coh.

See also Figure S3.

GFP-tagged Sgo1 or PP2A regulatory subunit, Rts1, showed similar kinetochore-proximal localization in live wild-type and *spo13Δ* cells progressing from prophase I into metaphase I (Figures 4A and 4B). Furthermore, Sgo1-6Ha ChIP-seq profiles were virtually indistinguishable in wild-type and *spo13Δ* metaphase I cells along a representative chromosome (Figure 4C), or averaged across all pericentromeres (Figure 4D), and ChIP-qPCR confirmed quantitatively similar levels (Figure 4E), despite slightly reduced cellular Sgo1 levels in *spo13Δ* cells (Figure 4F). Moreover, Sgo1 localization during metaphase I in *spo13Δ* cells corresponds to the domain of pericentromeric cohesin where Rec8 is known to be enriched and ordinarily protected in wild-type cells (Figure 4C; Kiburz et al., 2005). We conclude that meiotic cohesion loss in *spo13Δ* mutants cannot be explained by de-localization of Sgo1 from the pericentromere in metaphase I.

Spo13 Deters Cohesin Phosphorylation

Rec8 cleavage in anaphase I requires its prior phosphorylation, which is counteracted by Sgo1-PP2A at the pericentromere. Since Sgo1-PP2A is localized normally in *spo13Δ* cells, Spo13 must either preclude the requirement for cohesin phosphorylation, or counteract cohesin phosphorylation in parallel to Sgo1-PP2A. To distinguish between these possibilities, we compared the extent of pericentromeric Rec8 phosphorylation in wild-type, *spo13Δ*, and *SPO13*-overexpressing cells. To specifically isolate the pericentromeric pool of Rec8, we immunoprecipitated Sgo1 and used mass spectrometry to analyze relative changes in phosphorylation of co-precipitating Rec8. As a positive control, we immunoprecipitated Sgo1-3A, which cannot bind PP2A (Xu et al., 2009) and is therefore expected to increase pericentromeric Rec8 phosphorylation. Analysis of Sgo1's interaction partners confirmed its interaction with cohesin (unchanged

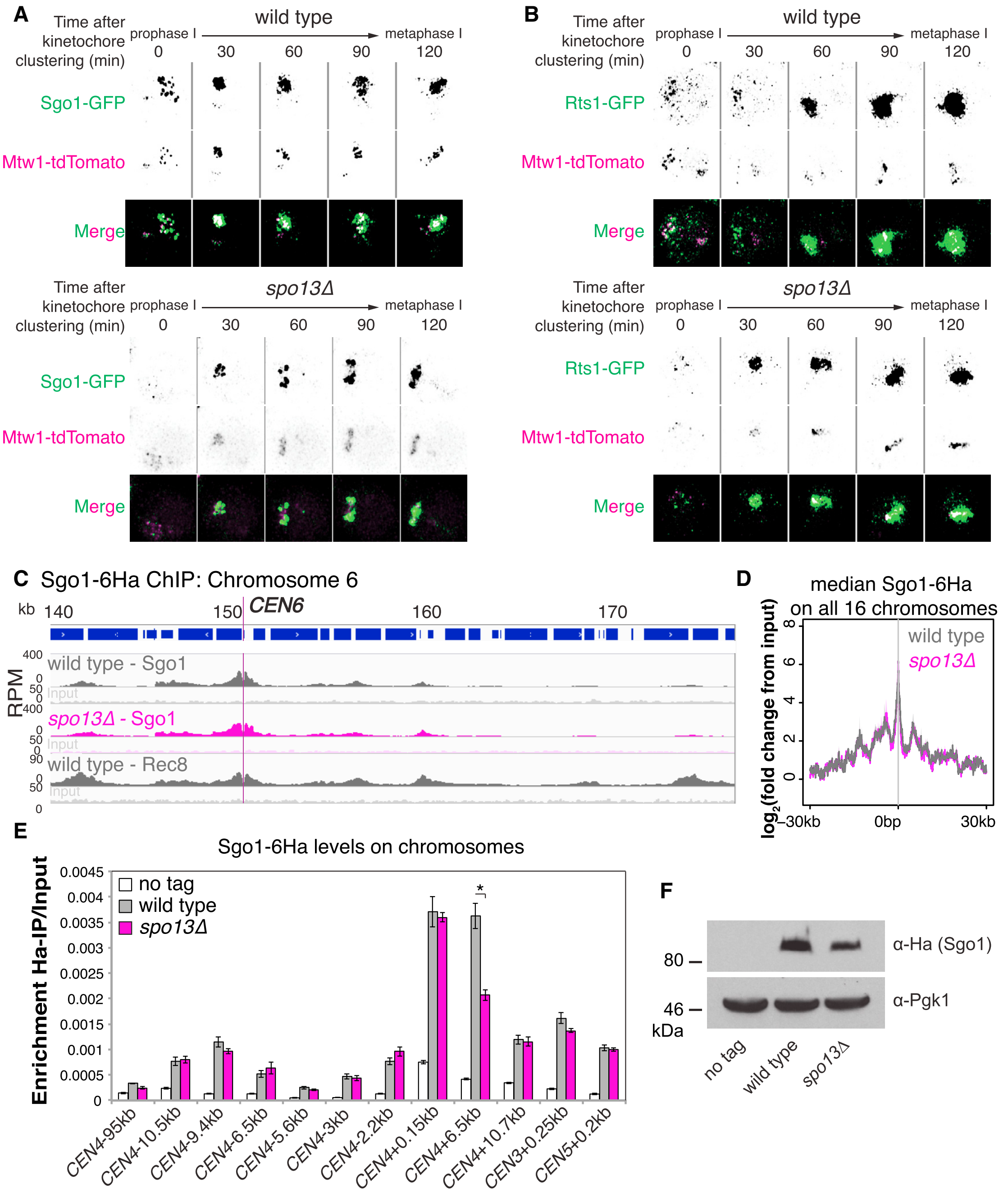

Figure 4. Cohesin Protection Is Set up in *spo13Δ* Mutants

(A and B) Representative images show co-localization of kinetochores (Mtw1-tdTomato) and either Sgo1-yEGFP (A) or Rts1-yEGFP (B) in wild-type and *spo13Δ* live cells.

(C and D) Sgo1-6Ha ChIP-Seq in wild-type and *spo13Δ* cells 75 min after release from prophase I. (C) Comparison of Sgo1 and Rec8 localization along chromosome 6. (D) Median composite Sgo1-6Ha signal surrounding all centromeres.

(E) Mean Sgo1 enrichment by ChIP-qPCR in *spo13Δ* mutants at metaphase I from four experimental repeats with standard error bars (*$p < 0.05$).

(F) Immunoblot analysis of Sgo1-6Ha and Pgk1 loading control on whole-cell extracts from (E).

in *spo13Δ*), specific loss of monopolin in *spo13Δ*, and loss of PP2A in the *sgo1-3A* mutant, as expected (Figure S4A). While Rec8 phosphopeptides were not detectably enriched in the *sgo1-3A* mutant over wild type, we observed a depletion of unphosphorylated Rec8 peptides (Figures 5A, 5B, S4B, and S4C), suggesting that Rec8 hyperphosphorylation precludes phosphopeptide detection. The changes in Rec8 phosphopeptides in *spo13Δ* were modest: while unphosphorylated Rec8 peptides were comparable in abundance to wild type, Rec8 phosphopeptides were mildly enriched over wild type

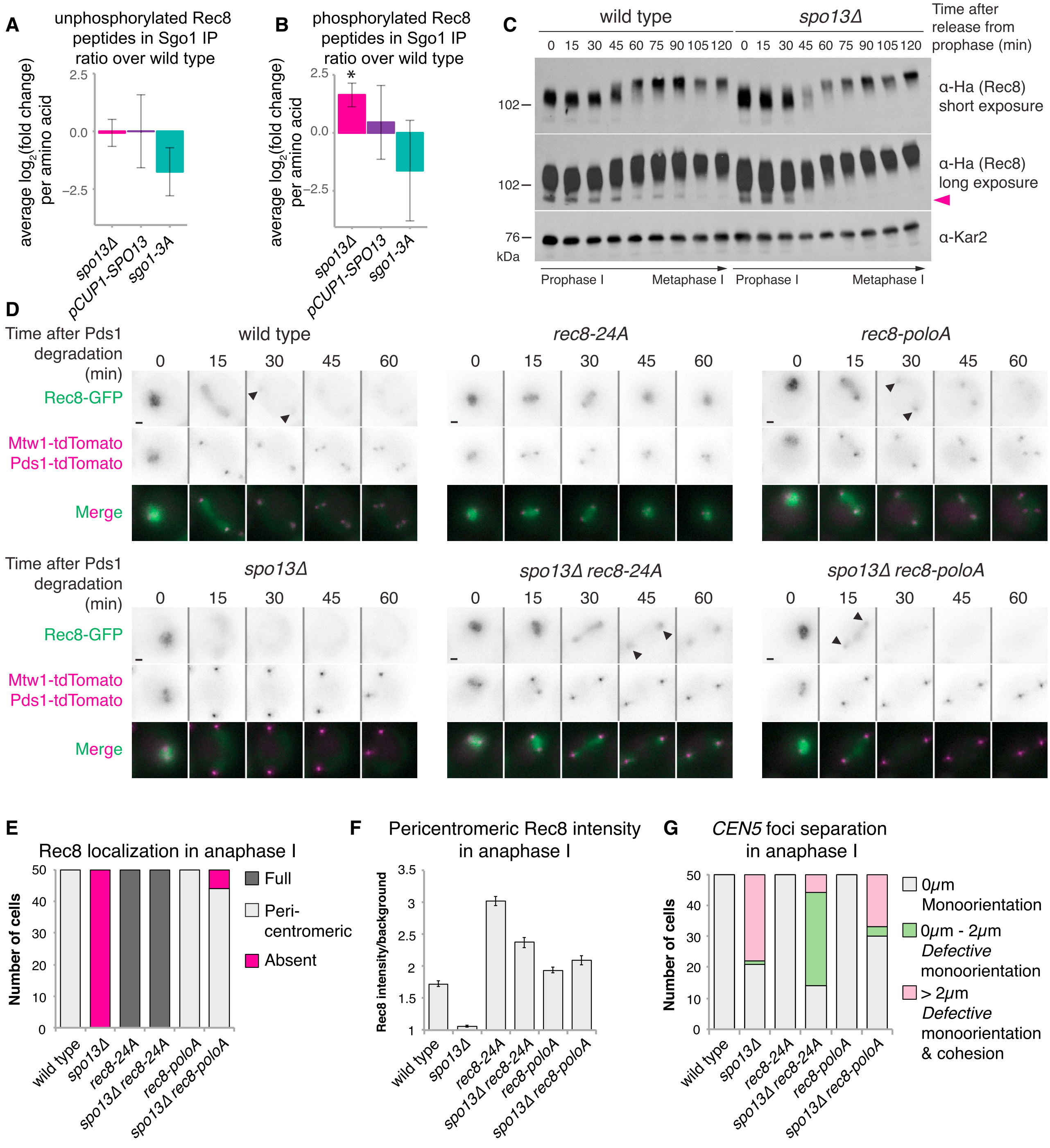

Figure 5. Cohesin Phosphorylation Is Required for Loss of Sister Chromatid Cohesion in *spo13Δ* Cells

(A and B) Average per residue ratio of $\log_2$(fold enrichment over wild type) of Rec8 non-phospho- (A) and phosphopeptides (B) in three replicate Sgo1 immunoprecipitates from cells harvested 75 min after release from prophase (metaphase I). Error bars represent standard deviation (*$p < 0.05$, one sample t test).

(C) Immunoblot analysis of Rec8-3Ha and Kar2 loading control.

(D–G) Effect of *SPO13* deletion on localization of Rec8-GFP and phosphomutant variants. (D) Representative images from time-lapse series. Scale bars, 1 μm. Arrows indicate pericentromeric Rec8-GFP. (E) Scoring and (F) intensity of pericentromeric Rec8-GFP with standard error bars. (G) Heterozygous *CEN5-GFP* distances as in Figure 2E using Pds1-tdTomato as anaphase I marker.

See also Figure S4.

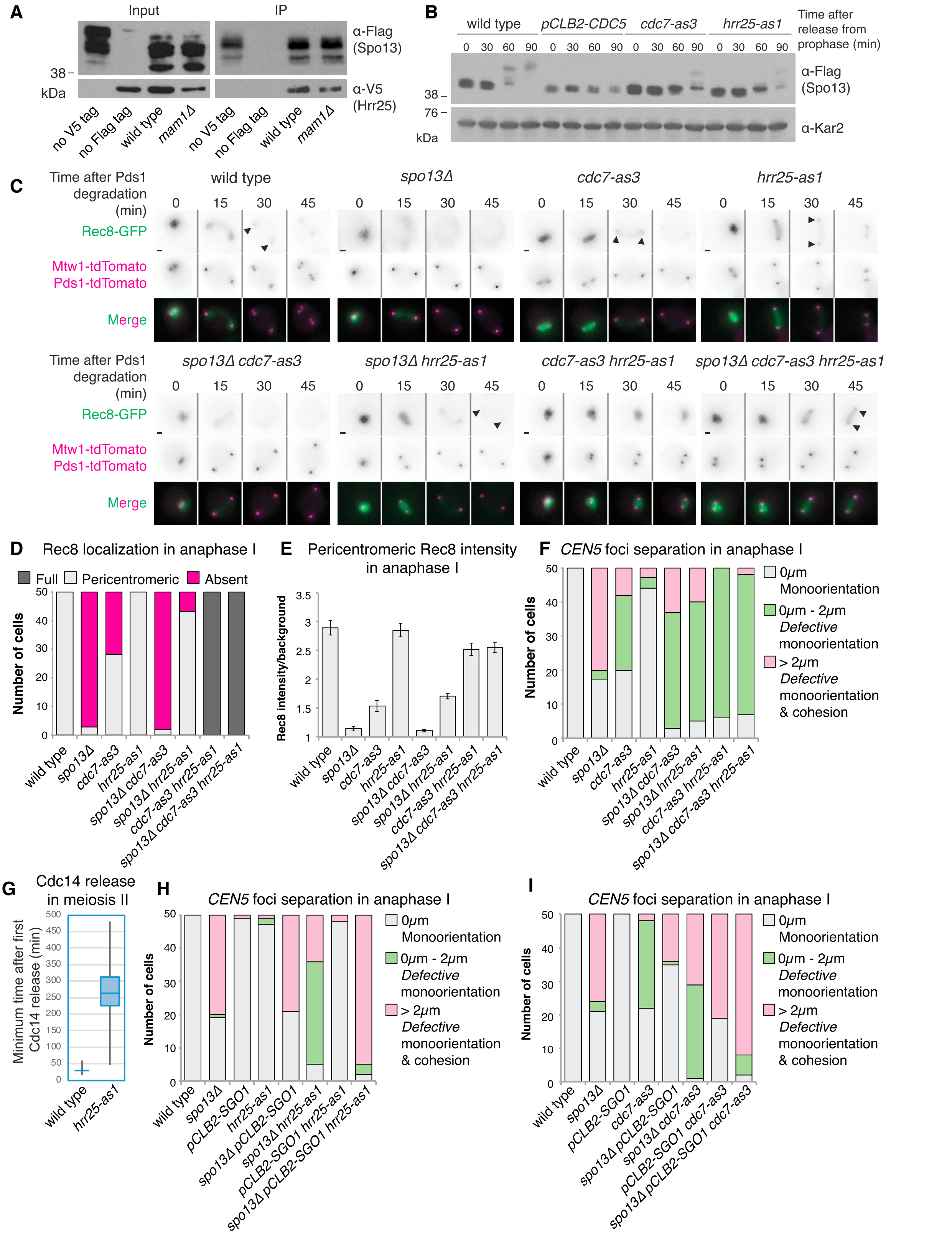

Figure 6. Inhibition of Cohesin Kinases Prevents Cohesion Loss in *spo13Δ* Mutants in an Sgo1-Dependent Manner

(A) Immunoblot shows Hrr25-V5 co-immunoprecipitates with Spo13-3Flag.

(B) Immunoblot analysis of Spo13-3Flag and Kar2 loading control after release from prophase into a metaphase I arrest.

(legend continued on next page)

(Figures 5A, 5B, S4B, and S4C). However, *SPO13* overexpression did not detectably alter the abundance of either phosphorylated or unphosphorylated Rec8 peptides (Figures 5A, 5B, S4B, and S4C). Western blot analysis of Rec8 mobility in cells progressing from prophase I into a metaphase I arrest confirmed that the faster migrating, unphosphorylated, and presumed pericentromeric form of Rec8 disappeared more quickly in *spo13Δ* cells than wild type (Figure 5C). Taken together, these findings are consistent with the idea that Spo13 deters cohesin phosphorylation.

Restricting Rec8 Phosphorylation Prevents Cohesin Loss in *spo13Δ* Cells

We tested whether reducing Rec8 phosphorylation can prevent cohesin loss during anaphase I by mutating only a subset of phosphorylation sites, thereby reducing overall phosphorylation but not completely abrogating cleavage. We mutated 11 previously identified and 3 putative Cdc5-dependent phosphorylation sites (Brar et al., 2006) to alanine (henceforth called Rec8-poloA) and used a non-phosphorylatable, and therefore uncleavable, version of Rec8 (Rec8-24A) (Katis et al., 2010) as a control. A separase biosensor (Yaakov et al., 2012), comprising a fragment of Rec8-GFP tethered to a chromosomal arm site (Figure S4D), confirmed that while the Rec8-24A mutations blocked cleavage, Rec8-poloA was cleaved in a manner indistinguishable from wild-type Rec8 in both wild-type and *spo13Δ* cells (Figures S4E–S4H). Next, we followed GFP-tagged Rec8 and phosphomutant variants through meiosis in otherwise wild-type or *spo13Δ* cells (Figure 5D). As expected, at anaphase I, Rec8-GFP persisted in the vicinity of the kinetochores in wild type but disappeared in *spo13Δ* cells (Figures 5D and 5E). In contrast, non-phosphorylatable *rec8-24A* prevented bulk cohesin cleavage whether or not Spo13 was present (Figures 5D and 5E). Interestingly, while the bulk of Rec8-poloA-GFP was lost from chromosomes, a small pool was retained in the pericentromere, even in *spo13Δ* cells, albeit transiently (Figures 5D and 5E). Consistently, the signal intensity of pericentromeric Rec8-poloA-GFP in *spo13Δ* anaphase I cells was comparable to that of either Rec8-GFP or Rec8-poloA-GFP in wild-type anaphase I cells (Figure 5F). Nevertheless, the transiently retained Rec8-poloA-GFP in *spo13Δ* cells could not prevent sister chromatid segregation during anaphase I, though the non-phosphorylatable Rec8-24A could (Figure 5G). The delay in pericentromeric cohesin cleavage in *rec8-poloA* cells lacking *SPO13*, despite timely loss of arm cohesin, suggests that it is the synergistic effects of the Rec8-poloA mutations and pericentromeric Sgo1-PP2A that impair cohesin cleavage. This indicates that a threshold level of Rec8 phosphorylation is required for its cleavage and that Sgo1-PP2A and Spo13 synergize to counteract this phosphorylation at the pericentromere.

Spo13 Prevents Premature Cohesion Loss by Limiting the Effects of the Cohesin Kinases

We hypothesized that Spo13 may ensure cohesin protection by regulating the cohesin kinases. Indeed, Spo13 binds Cdc5 and is inferred to interact with DDK, which itself is a Cdc5 binding factor (Matos et al., 2008). Hrr25-3V5 also co-immunoprecipitates with Spo13-3Flag, independent of Hrr25 recruitment to kinetochores by Mam1 (Figure 6A; Petronczki et al., 2006). Furthermore, full Spo13 phosphorylation depends not only on DDK and Cdc5 (Matos et al., 2008) but also on Hrr25 (Figure 6B). Therefore, Spo13 associates with, and is likely to be phosphorylated by, all three cohesin kinases.

We asked whether Spo13 prevents the cohesin kinases associating with chromosomes. However, chromosomal levels of Hrr25, Cdc7, or Cdc5 are not increased in *spo13Δ* cells (Figures 1A, S5A, and S5B). Rather, as expected, *spo13Δ* cells showed decreased levels of Hrr25 at centromeres, similar to cells lacking the monopolin subunit Mam1, which is known to recruit it (Figure S5A) and which itself is lost in *spo13Δ* cells (Katis et al., 2004b; Lee et al., 2004; Figure 2F).

Next, we tested whether Spo13 can influence the ability of the cohesin kinases to trigger phosphorylation-dependent Rec8 cleavage using specifically inhibitable versions of Hrr25 and Cdc7 (Hrr25-as1 and Cdc7-as3). During meiosis I, Hrr25 and Cdc7 trigger cleavage of chromosomal arm cohesin redundantly, so that inhibition of both kinases blocks all chromosome segregation, while inhibition of a single kinase delays Rec8 cleavage (Katis et al., 2010). We confirmed this using the Rec8-GFP separase biosensor targeted to a chromosomal arm site (where the phosphatase Sgo1-PP2A is absent) and further found that deletion of *SPO13* partially abrogated the cleavage delay caused by inhibition of Cdc7 but not Hrr25 (Figure S5C). As expected, inhibition of Hrr25 and Cdc7 also prevented removal of all endogenous Rec8-GFP in wild-type and *spo13Δ* cells (Figures 6C–6E). Surprisingly, however, Rec8-GFP was undetectable in 44% of anaphase I *cdc7-as3* mutants and virtually all *spo13Δ cdc7-as3* cells (Figure 6D). In contrast, inhibition of Hrr25-as1 in *spo13Δ* increased the number of cells with detectable pericentromeric Rec8-GFP and its average intensity in anaphase I (Figure 6E). We then examined the effects of Hrr25 and Cdc7 kinase inhibition on cohesion during anaphase I (Figure 6F). Both kinases are individually required for monopolin function (Matos et al., 2008; Petronczki et al., 2006), and inhibition of Cdc7, Hrr25, or both kinases increased sister kinetochore biorientation to some extent; however, centromeres separated only a short distance, indicating that centromeric cohesion is preserved, even in the *spo13Δ* background. Curiously, Hrr25 inhibition had only a minor effect on sister kinetochore biorientation in an otherwise wild-type background. This is not due to a failure to inhibit Hrr25 because meiosis II Cdc14 release, which specifically

(C–E) Inhibition of Hrr25 and Cdc7 in *spo13Δ* cells. (C) Representative time-lapse images. Scale bars, 1 μm. Arrows indicate pericentromeric cohesin. (D) Scoring of Rec8-GFP localization. (E) Intensity of pericentromeric Rec8-GFP with standard error bars.

(F) Heterozygous *CEN5-GFP* distance categorization upon Hrr25 and Cdc7 inhibition as in Figure 2E.

(G) Confirmation of *hrr25-as1* inhibition. Time between rounds of Cdc14 release measured from the experiment in Figure 6H. Boxes represent first and third quartile, whiskers indicate minimum and maximum values and the horizontal line shows the median.

(H and I) Sister chromatid cohesion upon Hrr25 (H) and Cdc7 (I) inhibition requires Sgo1. Heterozygous *CEN5-GFP* distances scored as in Figure 2E.

See also Figure S5.

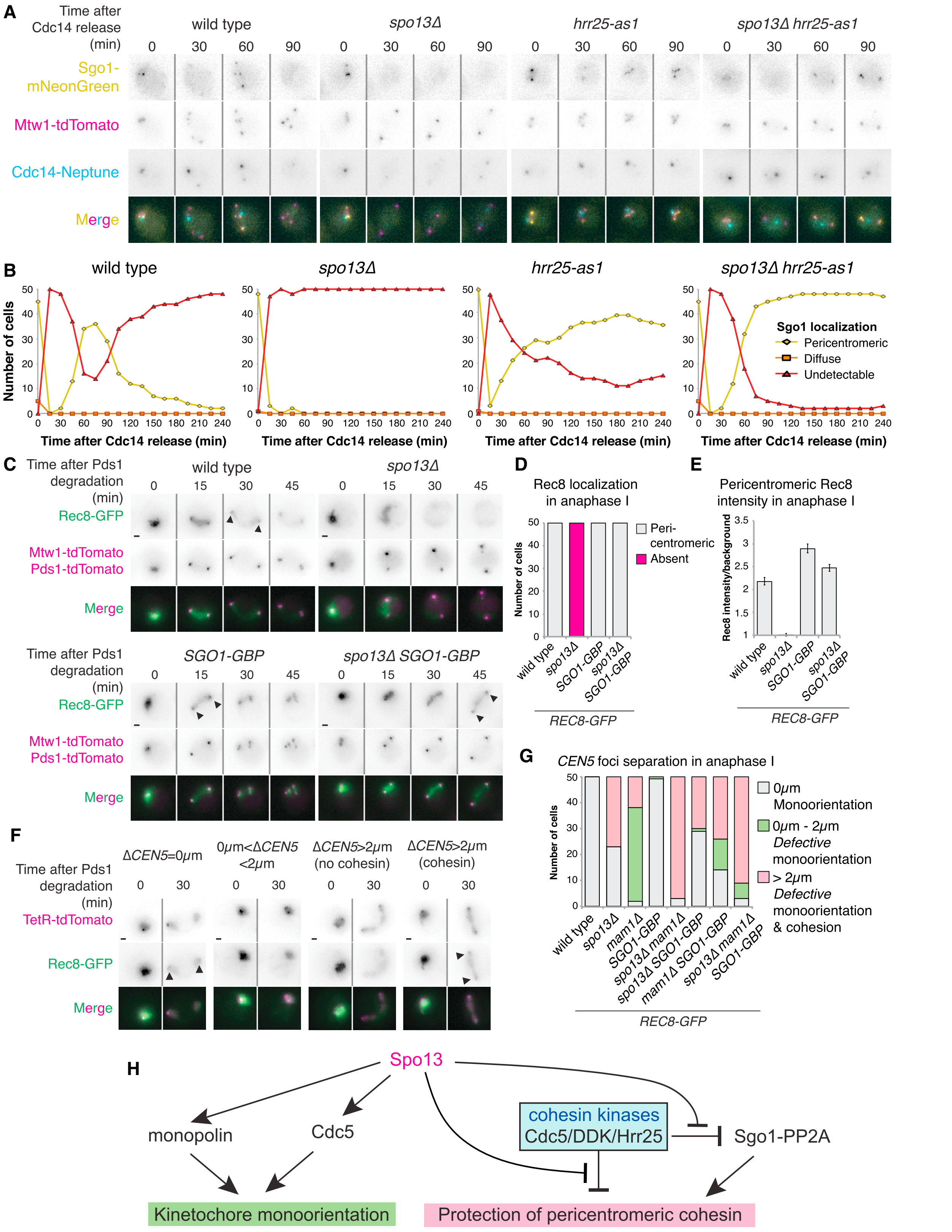

(legend on next page)

requires Hrr25, was blocked (Figure 6G) and in *spo13Δ* cells, sister chromatid segregation was prevented (Figure 6F). The reason why Hrr25-as1 inhibition has a more modest effect on monoorientation compared to *mam1Δ* remains unclear, but Hrr25 kinase activity may have a regulatory role since it is also not required for kinetochore fusion *in vitro* (Sarangapani et al., 2014). Unexpectedly, given that Rec8-GFP was undetectable in anaphase I (Figure 6D), *cdc7-as3* also prevents sister chromatid segregation in *spo13Δ* cells. Therefore, remarkably, inhibition of a single cohesin kinase is sufficient to restore cohesion to *spo13Δ* anaphase I cells. Depletion of Cdc5 in *spo13Δ* cells also prevented sister chromatid segregation (Figures S5D and S5E). However, this is partially overcome by abolishing recombination upon deleting *SPO11* (Figures S5D and S5E), indicating that the perceived sister chromatid cohesion in *spo13Δ pCLB2-CDC5* cells is in part due to the requirement for Cdc5 to resolve DNA joint molecules (Clyne et al., 2003; Matos et al., 2011). Furthermore, the faster migrating unphosphorylated Rec8 isoforms, likely the pericentromeric pool, do not persist in cells lacking both Sgo1 and Cdc5 (Figure 3B). This indicates that Cdc5 is not essential for phosphorylation of the pericentromeric pool of cohesin. Together, these observations demonstrate that reducing cohesin phosphorylation by inhibition of individual cohesin kinases, Hrr25 and DDK, is sufficient to prevent sister chromatid separation in the absence of *SPO13*.

Sgo1-PP2A and Spo13-Dependent Regulation of Cohesin Kinases together Protect Pericentromeric Cohesin

Our findings suggest that Spo13 regulates cohesin cleavage by counteracting the effects of the cohesin kinases, thereby synergizing with Sgo1-PP2A to maintain pericentromeric cohesin phosphorylation below a threshold important for its cleavage. If this idea were correct, inactivation of the Sgo1-PP2A phosphatase would be expected to restore pericentromeric cohesin cleavage in *spo13Δ* cells where a single kinase is inhibited. Indeed, Hrr25 or Cdc7 inhibition blocked sister chromatid segregation in *spo13Δ* mutants only in the presence of Sgo1 (Figures 6H and 6I). Similarly, depletion of Sgo1 allows spindle elongation and sister chromatid segregation in *pCLB2-CDC5* cells (Brar et al., 2006). Since Sgo1 is restricted to the pericentromere, the relevant cohesion that prevents sister chromatid segregation in *spo13Δ hrr25-as1* and *spo13Δ cdc7-as3* cells must also reside in the pericentromere. Importantly, this confirms that endogenous Sgo1 is functional in cells lacking Spo13, at least in the absence of cohesin kinase activity, reinforcing the conclusion that Spo13 and Sgo1-PP2A protect pericentromeric cohesin through parallel and non-redundant pathways. Together, these results indicate that Spo13 counteracts Rec8 phosphorylation by cohesin kinases to prevent its cleavage.

Spo13 Influences Sgo1 through Control of Cohesin Kinases

Our data indicate that the cohesin kinases promote cohesion loss in *spo13Δ* cells by directly phosphorylating Rec8 to promote its cleavage. However, Hrr25 is also known to promote Sgo1 removal from chromosomes during meiosis II (Argüello-Miranda et al., 2017), so Spo13-dependent regulation of the cohesin kinases might additionally be important for maintenance of Sgo1-PP2A function during meiosis I. Indeed, inhibition of either *hrr25-as1* or *cdc7-as3*, or depletion of Cdc5, increased total cellular Sgo1 levels and its association with centromeres (Figures S6A–S6E). Curiously, and for reasons that are unclear, centromeric Sgo1 levels in *hrr25-as1 cdc7-as3* double mutants are comparable to wild type (Figure S6A), suggesting that these kinases influence Sgo1 localization both positively and negatively. If Spo13 counteracts the effects of cohesin kinases, then its overproduction would be expected to both increase chromosomally associated Sgo1 and enhance cohesin protection in meiosis I. Indeed, centromeric Sgo1 levels (Figure S6F) and the intensity of pericentromeric Rec8-GFP in anaphase I were increased upon *SPO13* overexpression, with the latter being dependent on the presence of Sgo1 (Figures S6G and S6H).

Our findings implicate Spo13 in restricting cohesin kinases, which in turn negatively regulate Sgo1 association with chromosomes (Figures S6A–S6E; Argüello-Miranda et al., 2017), yet Sgo1-PP2A is normally localized in *spo13Δ* metaphase I cells (Figure 4). Since cohesin cleavage occurs only at anaphase I onset, we reasoned that the effects of *SPO13* deletion on Sgo1-PP2A might only be apparent at this stage. In wild type, pericentromeric Sgo1-mNG is detected in metaphase I, greatly reduces in intensity at anaphase I onset, re-accumulates during metaphase II, and disappears during anaphase II (Figures 7A and 7B). In contrast, Sgo1 permanently disappears at anaphase I onset in *spo13Δ* cells (Figures 7A and 7B). Remarkably, inhibition of Hrr25-as1, but not Cdc7-as3, caused the reappearance of Sgo1 at pericentromeres after anaphase I onset in *spo13Δ* cells (Figures 7A, 7B, S6I, and S6J). Sgo1 reappearance in anaphase I does not require the persistence of pericentromeric cohesin since it also occurred in *spo13Δ hrr25-as1* cells expressing non-protectable Rec8-18D (Figures S6K and S6L). We conclude that Hrr25 removes Sgo1 from chromosomes upon anaphase I onset in *spo13Δ* cells.

Figure 7. Contribution of Post-Metaphase I Sgo1 Loss to Defective Cohesion in *spo13Δ* Cells

(A and B) Time lapse of Sgo1 returning to the pericentromere after anaphase I onset. (A) Representative images. (B) Scoring of Sgo1 localization in 50 cells for 4 h after initial Cdc14 release.

(C–E) Sgo1 tethering to Rec8. (C) Representative images. Scale bars, 1 μm. Arrows indicate pericentromeric cohesin. (D) The presence of pericentromeric cohesin in anaphase I was scored in 50 cells. (E) Average intensity of pericentromeric Rec8-GFP with standard error bars.

(F and G) Sister chromatid cohesion upon tethering of Sgo1 to Rec8. (F) Representative images from time-lapse. Scale bars, 1 μm. Arrows indicate pericentromeric cohesin. (G) Categories of heterozygous *CEN5-GFP* at anaphase I onset scored by measuring the maximum distance between two TetR-tdTomato foci within two time points after bulk Rec8 cleavage for 50 cells per strain.

(H) Model for regulation of meiosis I chromosome segregation by Spo13. For details, see text.

See also Figures S6 and S7.

Cohesin-Tethered Sgo1 Restores Pericentromeric Cohesin, but Not Cohesion, to *spo13Δ* Cells

To determine whether premature Sgo1 removal causes cohesin loss in *spo13Δ* cells, we used a version of Sgo1 lacking its recognition site for APC/C^{Cdc20}-dependent degradation (Eshleman and Morgan, 2014), which persists at pericentromeres during anaphase I (Figures S7A and S7B), and imaged Rec8-GFP throughout meiosis (Figure S7C). Interestingly, weak pericentromeric Rec8-GFP foci were detected in 38% of *spo13Δ sgo1Δdb* cells (Figure S7D), although the intensity was barely increased over that observed in *spo13Δ* cells (Figure S7E). Despite this apparent retention of pericentromeric cohesin in *spo13Δ sgo1Δdb* cells, we found that sister chromatid segregation frequency was nearly identical to that observed in *spo13Δ* cells (Figure S7F). These results suggest that preventing Sgo1 degradation during anaphase I is insufficient to restore sister chromatid cohesion to *spo13Δ* cells.

While Sgo1Δdb persisted at pericentromeres and marginally increased Rec8 levels during anaphase I, it may be subject to Hrr25-dependent removal and not be robustly associated with cohesin, explaining why it is incapable of restoring sister chromatid cohesion. As a complementary approach, we irreversibly forced Sgo1-cohesin interaction using the GFP-GBP tethering system, therefore ensuring continued Rec8-Sgo1 interaction at anaphase I onset. Strikingly, forcing Rec8-Sgo1 interaction resulted in Rec8-GFP retention at the pericentromere in all *spo13Δ* anaphase I cells, with signal intensity comparable to that of wild-type cells (Figures 7C–7E). The majority of this signal corresponds to uncleaved Rec8, since tethering Sgo1-3A, which cannot bind PP2A or prevent cohesin cleavage, increased pericentromeric Rec8-GFP intensity only modestly during anaphase I (Figures S7G–S7I).

Surprisingly, forcing Rec8-Sgo1 interaction in *spo13Δ* mutants did not prevent the segregation of heterozygous *CEN5*-tdTomato foci to opposite poles in anaphase I, despite the presence of cohesin (Figures 7F and 7G). The persistence of pericentromeric cohesin that does not provide sister chromatid cohesion in *spo13Δ* cells with forced Rec8-Sgo1 interaction (Figures 7F and 7G) is reminiscent of the behavior of *spo13Δ* cells expressing *rec8-poloA* (Figures 5D–5G) or *CDC5-Kt* (Figures S3B–S3F). Equally, tethering of Sgo1 to the kinetochore component Nkp1 did not prevent sister chromatid segregation in anaphase I *spo13Δ* cells (Figure S7J). Overall, these findings show that preventing Hrr25-dependent Sgo1 removal from pericentromeres during anaphase I alone is insufficient to restore sister chromatid cohesion, although pericentromeric cohesin persists. We conclude that Spo13-dependent control of cohesin kinases protects pericentromeric cohesion both by preventing cohesin phosphorylation and by maintaining Sgo1-PP2A function in anaphase I.

DISCUSSION

Establishment of the reductional meiosis I chromosome segregation pattern requires a number of seemingly unrelated modifications to the chromosome segregation machinery. Intriguingly, accumulating evidence suggests that a central, meiosis-specific, regulatory protein establishes at least two of these processes: sister kinetochore monoorientation and cohesin protection. These key meiosis I regulators, which essentially convert mitosis into meiosis I, include fission yeast Moa1, mouse MEIKIN (Kim et al., 2015; Miyazaki et al., 2017), and budding yeast Spo13. Here, we have shown that while Spo13 elicits both sister kinetochore monoorientation and pericentromeric cohesion through regulation of key meiotic kinases, it does so via distinct mechanisms (Figure 7H). Sister kinetochore monoorientation is achieved by Spo13-dependent recruitment of Cdc5 to kinetochores. Pericentromeric cohesin protection is achieved by controlling the cohesin kinases, thereby preventing cohesin phosphorylation both directly and, by preventing Sgo1-PP2A removal, indirectly. Overall, we reveal that Spo13 orchestrates the action of key meiotic kinases to establish reductional chromosome segregation in meiosis I.

Kinase Recruitment to Kinetochores—a Mechanism to Bias toward Monoorientation?

The unifying feature of the meiosis I regulators, Spo13, Moa1, and MEIKIN, is interaction with their respective Polo kinases (Kim et al., 2015; Matos et al., 2008; Miyazaki et al., 2017). We show that centromeric recruitment of Cdc5 by Spo13 is required for sister kinetochore monoorientation. Tethering Cdc5 to kinetochores in *spo13Δ* mutants restored sister chromatid co-segregation at anaphase I. Remarkably, Cdc5-Kt enforces sister chromatid co-segregation independently of monopolin-directed kinetochore fusion. Instead, kinetochore-localized Cdc5 may bias sister kinetochores toward segregating to the same pole. Rather than monopolin-based monoorientation, which is specific to budding yeast, inducing this bias might be the conserved effect of Polo kinase recruitment to kinetochores by Spo13, Moa1, and MEIKIN. How such a bias might be achieved is unclear, but kinetochore-associated human Plk1 stabilizes initial kinetochore-microtubule attachments (Liu et al., 2012), and perhaps a similar activity of Spo13-Cdc5 might support sister kinetochore monoorientation. We note that since Cdc5-Kt also induces homolog co-segregation in the absence of monopolin, regulated recruitment of Cdc5 to kinetochores is likely to be important. Indeed, while Cdc5-Kt is constitutively targeted to kinetochores during meiosis, in wild-type cells Spo13 is degraded in anaphase I so specific kinetochore targeting of Cdc5 is restricted to metaphase I (Sullivan and Morgan, 2007).

In budding yeast, monopolin-dependent sister kinetochore fusion is the main requirement for sister kinetochore monoorientation. While the kinetochore localization of monopolin is perturbed in *spo13Δ* cells (Katis et al., 2004b; Lee et al., 2004; Matos et al., 2008), curiously, and despite a requirement for Cdc5 in recruiting monopolin to kinetochores (Clyne et al., 2003), Cdc5-Kt does not rescue monopolin association with kinetochores. This suggests that Spo13 recruits or maintains monopolin at kinetochores in a Cdc5-independent manner. The kinetochore recruitment of monopolin depends on its association with the core kinetochore protein, Dsn1, an interaction that is likely to be regulated by phosphorylation (Corbett et al., 2010; Plowman et al., 2018; Sarkar et al., 2013). Spo13 also associates with, and influences, two further kinases, DDK and Hrr25, both of which are required for monopolin association with kinetochores (Matos et al., 2008; Petronczki et al., 2006). It is conceivable that as we show for cohesin protection, Spo13 regulates sister kinetochore monoorientation by affecting multiple key kinases.

The Role of Cdc5 in Cohesin Removal

Previous reports have suggested that kinetochore recruitment of Cdc5 is the key function of fission yeast Moa1 and mouse MEIKIN in cohesin protection (Kim et al., 2015; Miyazaki et al., 2017). In budding yeast, however, the role of Cdc5 in cohesin function during meiosis has been controversial. While it is agreed that Cdc5 binds to and phosphorylates cohesin, it was unclear whether Cdc5-dependent cohesin phosphorylation contributes to cohesin cleavage (Attner et al., 2013; Brar et al., 2006; Katis et al., 2010). Indeed, conversely, at least in cells lacking the meiosis-specific APC/C activator Ama1, Cdc5 promotes cohesin protection (Katis et al., 2010). We have provided a possible solution to this apparent paradox, as we demonstrate that Cdc5 affects cohesin cleavage in different ways, depending on its localization. Cohesin-tethered Cdc5 promotes loss of pericentromeric cohesion. Whether this is due to cohesin cleavage or via the cleavage-independent cohesin removal pathway mediated by Rad61/Wapl (Challa et al., 2016, 2019; Yu and Koshland, 2005) remains to be determined. In contrast, kinetochore-bound Cdc5 enhances pericentromeric cohesin retention in anaphase I, though this does not confer functional cohesion. Therefore, while Spo13-dependent Cdc5 recruitment to kinetochores may support centromeric cohesin protection, as reported for fission yeast Moa1 and mouse MEIKIN (Kim et al., 2015; Miyazaki et al., 2017), other functions of Spo13 are essential.

Cohesin Kinases Drive Premature Loss of Pericentromeric Cohesion in *spo13Δ* Cells

The role of Spo13 and its functional homologs in cohesin protection has long been elusive. Although a function in localizing Sgo1 has been suggested (Kiburz et al., 2005; Kim et al., 2015; Miyazaki et al., 2017), we and others, could not find any evidence that the centromeric localization of Sgo1 in metaphase I is altered in the absence of Spo13 (Lee et al., 2004). Instead, we reveal that cohesin kinases are the main driver of premature cohesin loss in *spo13Δ* cells via a two-pronged mechanism, affecting both cohesin itself and its protector, Sgo1-PP2A. Firstly, inhibition of Hrr25 and Cdc7 or depletion of Cdc5, all restored sister chromatid cohesion in the absence of Spo13 and this was dependent on Sgo1. The simplest interpretation of these data is that Spo13 and Sgo1-PP2A together ensure that Rec8 phosphorylation is maintained at a level below that sufficient for Rec8 cleavage. Since Sgo1 is restricted to the pericentromere, only Rec8 in this region will be below the phosphorylation threshold required for cleavage. Secondly, inhibition of Hrr25 allows Sgo1 reaccumulation after anaphase I onset in *spo13Δ* cells. Therefore, Spo13-dependent Hrr25 restraint also promotes cohesin protection by maintaining Sgo1 at pericentromeres in anaphase I cells. Interestingly, Hrr25-mediated Sgo1 removal might explain the eventual cleavage of pericentromeric Rec8-poloA, which transiently persists into anaphase I in *spo13Δ* cells (Figures 5D–5F). While forcibly tethering Sgo1 to Rec8 partially restored pericentromeric cohesin, it failed to support centromeric cohesion. Therefore, although Spo13-dependent reaccumulation of Sgo1-PP2A likely facilitates cohesion protection, it is not the only important effect of Spo13-dependent cohesin kinase control.

Our findings also highlight an important paradox: how does Spo13 antagonize the cohesin kinases in anaphase I, when Spo13 is normally degraded (Sullivan and Morgan, 2007)? Hrr25 is required for meiosis II exit and this function must be inhibited in meiosis I (Argüello-Miranda et al., 2017). Our findings that Spo13 interacts with Hrr25 and that inhibition of this kinase specifically rescued multiple *spo13Δ*-associated phenotypes leads us to speculate that Spo13 could act as an inhibitor of Hrr25-mediated meiotic exit and that cohesin deprotection in the absence of Spo13 might be an inevitable side effect of this process occurring prematurely.

STAR★METHODS

Detailed methods are provided in the online version of this paper and include the following:

- KEY RESOURCES TABLE
- CONTACT FOR REAGENT AND RESOURCE SHARING
- EXPERIMENTAL MODEL AND SUBJECT DETAILS
  - Yeast Strains and Plasmids
  - Growth Conditions
- METHOD DETAILS
  - Chromatin Immunoprecipitation
  - Immunoprecipitation
  - Western Blotting
  - Mass Spectrometry
  - Immunofluorescence and Fixed-Cell Microscopy
  - Live-Cell Imaging
- QUANTIFICATION AND STATISTICAL ANALYSIS
- DATA AND SOFTWARE AVAILABILITY

SUPPLEMENTAL INFORMATION

Supplemental Information can be found online at https://doi.org/10.1016/j.devcel.2019.04.003.

ACKNOWLEDGMENTS

We thank Robin Allshire, Angelika Amon, Kevin Hardwick, Nancy Hollingsworth, Heinrich Leonhardt, David Morgan, Ulrich Rothbauer, and Wolfgang Zachariae for yeast strains and plasmids; Ken Sawin for sharing equipment; John Weir, Hongtao Yu, and members of the Marston lab for discussion; and Eris Duro and Nadine Vincenten for comments on the manuscript. This research was funded by Wellcome through PhD studentships to S.G. (096994) and R.E.B. (102316), Senior Research Fellowships to A.L.M. (107827) and J.R. (103139), an instrument grant (108504), and core funding for The Wellcome Centre for Cell Biology (203149).

AUTHOR CONTRIBUTIONS

Conceptualization, S.G. and A.L.M.; Methodology, S.G., R.E.B., W.E.B., and C.S.; Investigation, S.G., R.E.B., and W.E.B.; Software, D.A.K.; Formal analysis, D.R. W.E.B, and C.S.; Writing – Original Draft, S.G. and A.L.M.; Writing – Review and Editing, S.G., A.L.M., W.E.B., and R.E.B.; Visualization, S.G., W.E.B., and A.L.M.; Supervision, A.L.M. and J.R.; Funding acquisition, A.L.M.

DECLARATION OF INTERESTS

The authors declare no competing interests.

Received: March 27, 2018
Revised: December 5, 2018
Accepted: March 31, 2019
Published: April 25, 2019

REFERENCES

Argüello-Miranda, O., Zagoriy, I., Mengoli, V., Rojas, J., Jonak, K., Oz, T., Graf, P., and Zachariae, W. (2017). Casein kinase 1 coordinates cohesin cleavage, gametogenesis, and exit from M Phase in meiosis II. Dev. Cell *40*, 37–52.

Attner, M.A., Miller, M.P., Ee, L.S., Elkin, S.K., and Amon, A. (2013). Polo kinase Cdc5 is a central regulator of meiosis I. Proc. Natl. Acad. Sci. USA *110*, 14278–14283.

Benjamin, K.R., Zhang, C., Shokat, K.M., and Herskowitz, I. (2003). Control of landmark events in meiosis by the CDK Cdc28 and the meiosis-specific kinase Ime2. Genes Dev. *17*, 1524–1539.

Brar, G.A., Kiburz, B.M., Zhang, Y., Kim, J.E., White, F., and Amon, A. (2006). Rec8 phosphorylation and recombination promote the step-wise loss of cohesins in meiosis. Nature *441*, 532–536.

Buonomo, S.B., Clyne, R.K., Fuchs, J., Loidl, J., Uhlmann, F., and Nasmyth, K. (2000). Disjunction of homologous chromosomes in meiosis I depends on proteolytic cleavage of the meiotic cohesin Rec8 by separin. Cell *103*, 387–398.

Carlile, T.M., and Amon, A. (2008). Meiosis I is established through division-specific translational control of a cyclin. Cell *133*, 280–291.

Challa, K., Lee, M.S., Shinohara, M., Kim, K.P., and Shinohara, A. (2016). Rad61/Wpl1 (Wapl), a cohesin regulator, controls chromosome compaction during meiosis. Nucleic Acids Res. *44*, 3190–3203.

Challa, K., Fajish, V.G., Shinohara, M., Klein, F., Gasser, S.M., and Shinohara, A. (2019). Meiosis-specific prophase-like pathway controls cleavage-independent release of cohesin by Wapl. PLoS Genet. *5*, e1007851.

Chelysheva, L., Diallo, S., Vezon, D., Gendrot, G., Vrielynck, N., Belcram, K., Rocques, N., Márquez-Lema, A., Bhatt, A.M., Horlow, C., et al. (2005). AtREC8 and AtSCC3 are essential to the monopolar orientation of the kinetochores during meiosis. J. Cell Sci. *118*, 4621–4632.

Clyne, R.K., Katis, V.L., Jessop, L., Benjamin, K.R., Herskowitz, I., Lichten, M., and Nasmyth, K. (2003). Polo-like kinase Cdc5 promotes chiasmata formation and cosegregation of sister centromeres at meiosis I. Nat. Cell Biol. *5*, 480–485.

Corbett, K.D., Yip, C.K., Ee, L.S., Walz, T., Amon, A., and Harrison, S.C. (2010). The monopolin complex crosslinks kinetochore components to regulate chromosome-microtubule attachments. Cell *142*, 556–567.

Eshleman, H.D., and Morgan, D.O. (2014). Sgo1 recruits PP2A to chromosomes to ensure sister chromatid bi-orientation in mitosis. J Cell Sci. *127*, 4974–4983.

Galander, S., Barton, R.E., Kelly, D.A., and Marston, A.L. (2019). Spo13 prevents premature cohesin cleavage during meiosis. Wellcome Open Res. *4*, 29.

Hu, B., Petela, N., Kurze, A., Chan, K.L., Chapard, C., and Nasmyth, K. (2015). Biological chromodynamics: a general method for measuring protein occupancy across the genome by calibrating ChIP-seq. Nucleic Acids Res. *43*, e132.

Jonak, K., Zagoriy, I., Oz, T., Graf, P., Rojas, J., Mengoli, V., and Zachariae, W. (2017). APC/C-Cdc20 mediates deprotection of centromeric cohesin at meiosis II in yeast. Cell Cycle *16*, 1145–1152.

Katis, V.L., Gálová, M., Rabitsch, K.P., Gregan, J., and Nasmyth, K. (2004a). Maintenance of cohesin at centromeres after meiosis I in budding yeast requires a kinetochore-associated protein related to MEI-S332. Curr. Biol. *14*, 560–572.

Katis, V.L., Matos, J., Mori, S., Shirahige, K., Zachariae, W., and Nasmyth, K. (2004b). Spo13 facilitates monopolin recruitment to kinetochores and regulates maintenance of centromeric cohesion during yeast meiosis. Curr. Biol. *14*, 2183–2196.

Katis, V.L., Lipp, J.J., Imre, R., Bogdanova, A., Okaz, E., Habermann, B., Mechtler, K., Nasmyth, K., and Zachariae, W. (2010). Rec8 phosphorylation by casein kinase 1 and Cdc7-Dbf4 kinase regulates cohesin cleavage by separase during meiosis. Dev. Cell. *18*, 397–409.

Kiburz, B.M., Reynolds, D.B., Megee, P.C., Marston, A.L., Lee, B.H., Lee, T.I., Levine, S.S., Young, R.A., and Amon, A. (2005). The core centromere and Sgo1 establish a 50-kb cohesin-protected domain around centromeres during meiosis I. Genes Dev. *19*, 3017–3030.

Kim, J., Ishiguro, K.-I., Nambu, A., Akiyoshi, B., Yokobayashi, S., Kagami, A., Ishiguro, T., Pendás, A.M., Takeda, N., Sakakibara, Y., et al. (2015). Meikin is a conserved regulator of meiosis-I-specific kinetochore function. Nature *517*, 466–471.

Kitajima, T.S., Kawashima, S.A., and Watanabe, Y. (2004). The conserved kinetochore protein shugoshin protects centromeric cohesion during meiosis. Nature *427*, 510–517.

Kitajima, T.S., Sakuno, T., Ishiguro, K.-I., Iemura, S.-I., Natsume, T., Kawashima, S.A., and Watanabe, Y. (2006). Shugoshin collaborates with protein phosphatase 2A to protect cohesin. Nature *441*, 46–52.

Klapholz, S., and Esposito, R.E. (1980). Recombination and chromosome segregation during the single division meiosis in SPO12-1 and SPO13-1 diploids. Genetics *96*, 589–611.

Klein, F., Mahr, P., Gálová, M., Buonomo, S.B., Michaelis, C., Nairz, K., and Nasmyth, K. (1999). A central role for cohesins in sister chromatid cohesion, formation of axial elements, and recombination during yeast meiosis. Cell *98*, 91–103.

Kutrowska, B.W., Narczyk, M., Buszko, A., Bzowska, A., and Clark, P.L. (2007). Folding and unfolding of a non-fluorescent mutant of green fluorescent protein. J. Phys. Condens. Matter *19*, 285223.

Lee, B.H., and Amon, A. (2003). Role of Polo-like kinase CDC5 in programming meiosis I chromosome segregation. Science *300*, 482–486.

Lee, B.H., Kiburz, B.M., and Amon, A. (2004). Spo13 maintains centromeric cohesion and kinetochore coorientation during meiosis I. Curr. Biol. *14*, 2168–2182.

Lee, J., Kitajima, T.S., Tanno, Y., Yoshida, K., Morita, T., Miyano, T., Miyake, M., and Watanabe, Y. (2008). Unified mode of centromeric protection by shugoshin in mammalian oocytes and somatic cells. Nat. Cell Biol. *10*, 42–52.

Liu, D., Davydenko, O., and Lampson, M.A. (2012). Polo-like kinase-1 regulates kinetochore-microtubule dynamics and spindle checkpoint silencing. J. Cell Biol. *198*, 491–499.

MacLean, B., Tomazela, D.M., Shulman, N., Chambers, M., Finney, G.L., Frewen, B., Kern, R., Tabb, D.L., Liebler, D.C., and MacCoss, M.J. (2010). Skyline: an open source document editor for creating and analyzing targeted proteomics experiments. Bioinformatics *26*, 966–968.

Marston, A.L. (2014). Chromosome segregation in budding yeast: sister chromatid cohesion and related mechanisms. Genetics *196*, 31–63.

Marston, A.L., Tham, W.H., Shah, H., and Amon, A. (2004). A genome-wide screen identifies genes required for centromeric cohesion. Science *303*, 1367–1370.

Matos, J., Blanco, M.G., Maslen, S., Skehel, J.M., and West, S.C. (2011). Regulatory control of the resolution of DNA recombination intermediates during meiosis and mitosis. Cell *147*, 158–172.

Matos, J., Lipp, J.J., Bogdanova, A., Guillot, S., Okaz, E., Junqueira, M., Shevchenko, A., and Zachariae, W. (2008). Dbf4-dependent CDC7 kinase links DNA replication to the segregation of homologous chromosomes in meiosis I. Cell *135*, 662–678.

Miyazaki, S., Kim, J., Yamagishi, Y., Ishiguro, T., Okada, Y., Tanno, Y., Sakuno, T., and Watanabe, Y. (2017). Meikin-associated polo-like kinase specifies Bub1 distribution in meiosis I. Genes Cells *22*, 552–567.

Monje-Casas, F., Prabhu, V.R., Lee, B.H., Boselli, M., and Amon, A. (2007). Kinetochore orientation during meiosis is controlled by Aurora B and the monopolin complex. Cell *128*, 477–490.

Petronczki, M., Matos, J., Mori, S., Gregan, J., Bogdanova, A., Schwickart, M., Mechtler, K., Shirahige, K., Zachariae, W., and Nasmyth, K. (2006). Monopolar attachment of sister kinetochores at meiosis I requires casein kinase 1. Cell *126*, 1049–1064.

Plowman, R., Singh, N., Duro, E., Payan, A., Corbett, K.D., and Marston, A.L. (2018). The molecular basis of Monopolin recruitment to the kinetochore. bioRxiv, 456798.

Rabitsch, K.P., Petronczki, M., Javerzat, J.P., Genier, S., Chwalla, B., Schleiffer, A., Tanaka, T.U., and Nasmyth, K. (2003). Kinetochore recruitment of two nucleolar proteins is required for homolog segregation in meiosis I. Dev. Cell *4*, 535–548.

Rappsilber, J., Ishihama, Y., and Mann, M. (2003). Stop and go extraction tips for matrix-assisted laser desorption/ionization, nanoelectrospray, and LC/MS sample pretreatment in proteomics. Anal. Chem. *75*, 663–670.

Riedel, C.G., Katis, V.L., Katou, Y., Mori, S., Itoh, T., Helmhart, W., Gálová, M., Petronczki, M., Gregan, J., Cetin, B., et al. (2006). Protein phosphatase 2A protects centromeric sister chromatid cohesion during meiosis I. Nature *441*, 53–61.

Rothbauer, U., Zolghadr, K., Tillib, S., Nowak, D., Schermelleh, L., Gahl, A., Backmann, N., Conrath, K., Muyldermans, S., Cardoso, M.C., et al. (2006). Targeting and tracing antigens in live cells with fluorescent nanobodies. Nat. Methods *3*, 887–889.

Sakuno, T., Tada, K., and Watanabe, Y. (2009). Kinetochore geometry defined by cohesion within the centromere. Nature *458*, 852–858.

Sarangapani, K.K., Duro, E., Deng, Y., Alves, Fde L., Ye, Q., Opoku, K.N., Ceto, S., Rappsilber, J., Corbett, K.D., Biggins, S., et al. (2014). Sister kinetochores are mechanically fused during meiosis I in yeast. Science *346*, 248–251.

Sarkar, S., Shenoy, R.T., Dalgaard, J.Z., Newnham, L., Hoffmann, E., Millar, J.B.A., and Arumugam, P. (2013). Monopolin subunit Csm1 associates with MIND complex to establish monopolar attachment of sister kinetochores at meiosis I. PLoS Genet. *9*, e1003610.

Severson, A.F., Ling, L., van Zuylen, V., and Meyer, B.J. (2009). The axial element protein HTP-3 promotes cohesin loading and meiotic axis assembly in C. elegans to implement the meiotic program of chromosome segregation. Genes Dev. *23*, 1763–1778.

Shonn, M.A., McCarroll, R., and Murray, A.W. (2002). Spo13 protects meiotic cohesin at centromeres in meiosis I. Genes Dev. *16*, 1659–1671.

Sullivan, M., and Morgan, D.O. (2007). A novel destruction sequence targets the meiotic regulator Spo13 for anaphase-promoting complex-dependent degradation in anaphase I. J. Biol. Chem. *282*, 19710–19715.

Tang, Z., Shu, H., Qi, W., Mahmood, N.A., Mumby, M.C., and Yu, H. (2006). PP2A is required for centromeric localization of Sgo1 and proper chromosome segregation. Dev. Cell *10*, 575–585.

Tóth, A., Rabitsch, K.P., Gálová, M., Schleiffer, A., Buonomo, S.B., and Nasmyth, K. (2000). Functional genomics identifies monopolin: a kinetochore protein required for segregation of homologs during meiosis I. Cell *103*, 1155–1168.

Vincenten, N., Kuhl, L.M., Lam, I., Oke, A., Kerr, A.R., Hochwagen, A., Fung, J., Keeney, S., Vader, G., and Marston, A.L. (2015). The kinetochore prevents centromere-proximal crossover recombination during meiosis. Elife *4*, 923.

Wan, L., Zhang, C., Shokat, K.M., and Hollingsworth, N.M. (2006). Chemical inactivation of cdc7 kinase in budding yeast results in a reversible arrest that allows efficient cell synchronization prior to meiotic recombination. Genetics *174*, 1767–1774.

Watanabe, Y., and Nurse, P. (1999). Cohesin Rec8 is required for reductional chromosome segregation at meiosis. Nature *400*, 461–464.

Xu, Z., Cetin, B., Anger, M., Cho, U.S., Helmhart, W., Nasmyth, K., and Xu, W. (2009). Structure and function of the PP2A-shugoshin interaction. Mol. Cell *35*, 426–441.

Yaakov, G., Thorn, K., and Morgan, D.O. (2012). Separase biosensor reveals that cohesin cleavage timing depends on phosphatase PP2A(Cdc55) regulation. Dev. Cell *23*, 124–136.

Yokobayashi, S., Yamamoto, M., and Watanabe, Y. (2003). Cohesins determine the attachment manner of kinetochores to spindle microtubules at meiosis I in fission yeast. Mol. Cell. Biol. *23*, 3965–3973.

Yu, H.G., and Koshland, D. (2005). Chromosome morphogenesis: condensin-dependent cohesin removal during meiosis. Cell *123*, 397–407.

Zhang, X., Smits, A.H., van Tilburg, G.B., Ovaa, H., Huber, W., and Vermeulen, M. (2018). Proteome-wide identification of ubiquitin interactions using UbIA-MS. Nat. Protoc. *13*, 530–550.

STAR★METHODS

KEY RESOURCES TABLE

REAGENT or RESOURCE	SOURCE	IDENTIFIER
Antibodies		
Mouse anti-Ha (HA11)	BioLegend	Cat# MMS-101R; RRID: AB_291262
Mouse anti-Ha (12CA5)	Roche	Cat# 11583816001; RRID: AB_514505
Mouse anti-GFP	Sigma	Cat# 11814460001; RRID: AB_390913
Mouse anti-V5	Bio-Rad	Cat# MCA1360; RRID: AB_322378
Mouse anti-FLAG M2	Sigma	Cat# F1804; RRID: AB_262044
Rabbit anti-Kar2	Lab stock	N/A
Rabbit anti-Pgk1	Lab stock	N/A
Sheep anti-mouse HRP	GE Healthcare	Cat# NXA931; RRID: AB_772209
Donkey anti-rabbit HRP	GE Healthcare	Cat# NA934; RRID: AB_772206
Donkey anti-mouse IRDye 800CW	Li-COR Biosciences	Cat# 926-32212; RRID: AB_621847
Donkey anti-rabbit IRDye 680RD	Li-COR Biosciences	Cat# 926-68073; RRID: AB_10954442
Rat anti-tubulin	Bio-Rad	Cat# MCA77G; RRID: AB_325003
Donkey anti-rat FITC	Jackson ImmunoResearch	Cat# 712-095-153; RRID: AB_2340652
Chemicals, Peptides and Recombinant Proteins		
β-estradiol	Sigma	E2758
Benzonase®	Merck	71206-3
Chelex® 100	Bio-Rad	1422822
Proteinase K	Life Technologies	25530049
Dynabeads	ThermoFisher	10009D
PP1	Toronto Research Chemicals	A602980
1-NM-PP1	Toronto Research Chemicals	A603004
Trypsin	Pierce	90057
High-Select™ TiO2 Phosphopeptide Enrichment Kit	ThermoFisher	A32993
NuPage LDS Sample buffer	ThermoFisher	NP0008
Quick Blunting kit	NEB	E1201S
Klenow fragment	NEB	M0212S
Quick Ligation kit	NEB	M2200S
Phusion® High-Fidelity DNA polymerase	NEB	M0530S
AMPure XP beads	Beckman Coulter	A63881
Aprotinin	Melford	A2301
Chymostatin	Melford	C1104
Leupeptin (Hemisulphate)	Melford	L1001
E64	Melford	E1101
Pepstatin A	Melford	P2203
Antipain, dihydrochloride	Melford	A0105
AEBSF hydrochloride 98%	ACROS Organics	32811010
N-Ethylmaleimidine 99+%	ACROS Organics	156100050
COmplete-EDTA-free tablets	Roche	11873580001
Microcystin-L	LKT Laboratories	M3406
Glusulase	Perkin Elmer	NEE154001EA
Zymolyase	AMS Biotechnology (Europe)	120491-1
p-phenylenediamine	Sigma-Aldrich	695106

(*Continued on next page*)

Continued		
REAGENT or RESOURCE	SOURCE	IDENTIFIER
Deposited Data		
ChIP-seq data has been deposited on the Genome Expression Omnibus (GEO) and can be accessed using accession numbers	This study	GSE112167 (Sgo1-6Ha), GSE112170 (Spo13-3Flag) and GSE123546 (Rec8-3Ha).
The mass spectrometry proteomics data have been deposited to the ProteomeXchange Consortium via the PRIDE partner repository.	This study	PXD012627
Experimental Models: Organisms/Strains		
Yeast strains used in this study	n/a	See Table S1
Oligonucleotides		
Oligonucleotides used in this study for qPCR	n/a	See Table S3
Recombinant DNA		
Plasmids generated in this study	n/a	See Table S2
Software		
Image J plugin "DV_DotCounter" for analysis of microscopy data is available on Github	This study and Galander et al., 2019	https://doi.org/10.5281/zenodo.2553082)
Image J plugin "YeastLineProfiler" for analysis of microscopy data is available on Github	This study and Galander et al., 2019	https://doi.org/10.5281/zenodo.2560099
Other		
NEXTflex-6 DNA Barcodes	Perkin Elmer	514101

CONTACT FOR REAGENT AND RESOURCE SHARING

Further information and requests for resources and reagents should be directed to and will be fulfilled by Lead Contact, Adele L. Marston (adele.marston@ed.ac.uk).

EXPERIMENTAL MODEL AND SUBJECT DETAILS

Yeast Strains and Plasmids

All yeast strains are SK1 derivatives and are listed in Table S1. Plasmids generated in this study are listed in Table S2. Gene deletions, promoter replacements and gene tags were introduced using standard PCR-based methods. Specific depletion of proteins (Sgo1, Cdc20, Cdc5) during meiosis was achieved by placement of genes under the mitosis-specific *CLB2* promoter (Lee and Amon, 2003). For prophase block-release experiments, strains carried *pGAL1-NDT80*, *pGPD1-GAL4.ER* (Benjamin et al., 2003). Strains carrying *rec8-24A* (Katis et al., 2010), *rec8-18D* (Argüello-Miranda et al., 2017) and *hrr25-as1* (Petronczki et al., 2006) were described previously. GFP binding protein (GBP) (Rothbauer et al., 2006), *cdc7-as3* (Wan et al., 2006), *RTS1-GFP* (Katis et al., 2010) and separase biosensor constructs (Yaakov et al., 2012) were kind gifts from Ulrich Rothbauer and Heinrich Leonhardt, Nancy Hollingsworth, Wolfgang Zachariae and David Morgan, respectively. A yeast strain with *rec8-poloA* was generated from a synthetic gene construct (GeneArt) which carries the mutations S136A, T173A, S179A, S197A, S199A, S215A, T249A, S285A, S386A, S387A, S410A, S421A, S465A and S466A. Non-fluorescent GFP (nfGFP) was generated by introduction of the S65T and G67A mutations as described (Kutrowska et al., 2007).

Growth Conditions

Cells were prepared for sporulation as described by Vincenten et al. (2015). Briefly, after cryostorage diploid cells were thawed on YPG plates (1% yeast extract, 2% Bacto peptone, 2.5% glycerol, and 2% agar) for 16 h, before growing on 4% YPDA plates for 8-24 h (1% yeast extract, 2% Bacto peptone, 4% glucose, 2% agar and 0.3mM adenine). A small amount of culture was inoculated into liquid YPDA (1% yeast extract, 2% Bacto peptone, 2% glucose and 0.3mM adenine) and grown for 24 h. Cultures were diluted to OD_{600} = 0.3-0.5 in BYTA (1% yeast extract, 2% Bacto tryptone, 1% potassium acetate, 50mM potassium phthalate) medium and grown for approximately 16 h. Cells were washed twice with sterile deionised water and resuspended in sporulation medium (SPO; 0.3% potassium acetate, pH 7) at OD_{600} = 2.5. All growth steps were performed at 30°C. For prophase block-release, experiments were performed as above except that 5-6 h after resuspension in SPO medium, β-estradiol was added to 1μM to induce release from the prophase arrest, as outlined by Carlile and Amon (2008). To inhibit Hrr25-as1 and Cdc7-as3, cells were treated with 5μM 1-NM-PP1 (Toronto Research Chemicals) and 20μM PP1 (Toronto Research Chemicals), respectively. In experiments where at least one strain carried *cdc7-as3*, all strains also carried *pGAL-NDT80* and *GAL4-ER* to enable synchronous release from a prophase I arrest (Carlile and Amon, 2008) and PP1 (and 1-NM-PP1, if *hrr25-as1* was also part of the experiment) was added

at the same time as β-estradiol i.e. at prophase exit. In experiments where Hrr25-as1 was the only kinase to inhibit, 1-NM-PP1 was added 1 h after resuspending cells in SPO. This procedure was followed because Hrr25-as1 inhibition does not impact meiotic progression until meiotic exit whereas Cdc7-as3 inhibition results in meiotic arrest either in S phase or prophase.

For overexpression experiments using the copper-inducible promoter, *pCUP1*, 50 μM $CuSO_4$ was added to metaphase I-arrested (*pCLB2-CDC20*) cells 4.5 h after resuspension in SPO medium. In case of prophase arrest/release experiments, 50 μM $CuSO_4$ was added 30 min prior to release with β -estradiol. For the experiments with the separase biosensor, cells were supplied with 100 nM $CuSO_4$ at the time of resuspension in SPO medium.

METHOD DETAILS

Chromatin Immunoprecipitation

ChIP-qPCR and ChIP-seq were performed as previously described (Vincenten et al., 2015). Briefly, cells were harvested and washed twice in TBS buffer (20 mM Tris-HCl pH7.5, 150 mM NaCl) and once in FA lysis buffer (100 mM HEPES-KOH pH7.5, 300 mM NaCl, 2 mM EDTA, 2% TritonX, 0.2% Na Deoxycholate) containing 0.1% SDS (FA lysis buffer/0.1% SDS). Cell pellets were resuspended in 300μl FA lysis buffer/0.5% SDS containing 1x cOmplete protease inhibitor (PI) cocktail (Roche) and 1mM PMSF and lysed using silicon beads in FastPrep-24 homogeniser (MP Biomedicals). The mixture was centrifuged and washed once with FA lysis buffer/0.1% SDS+PI/PMSF. Cells were resuspended in 500μl FA lysis buffer/0.1% SDS+PI/PMSF and disrupted in a Bioruptor Plus sonicating water bath (Diagenode). Cell debris was removed by centrifugation. 500μl FA lysis buffer/0.1% SDS+PI/PMSF was added to the supernatant. After a further round of centrifugation, 300μl FA lysis buffer/0.1% SDS+PI/PMSF were added to the supernatant and 10μl of this solution was removed as Input. IP was performed overnight using 1ml of cell lysate and 7.5μl mouse anti-Ha (12CA5, Roche), 5μl mouse anti-Flag (M2, Sigma), 10μl mouse anti-V5 (SV5-Pk1, Bio-Rad) or 10μl mouse anti-GFP (Roche) together with 15μl of prewashed Protein G-conjugated Dynabeads (Life Technologies). After overnight incubation, IPs were washed in a tube magnet successively with 1ml of each of CWB1 (FA lysis buffer/0.1% SDS/ 275 mM NaCl), CWB2 (FA lysis buffer/0.1% SDS/ 500 mM NaCl), CWB3 (10 mM Tris-HCl, pH 8, 0.25 M LiCl, 1mM EDTA, 0.5% NP-40, 0.5% Na Deoxycholate) and TE (10 mM Tris-HCl, pH 8, 1 mM EDTA). Remaining wash buffer was removed and 200μl 10% Chelex 100 (Bio-Rad) solution was added to both IP and Input before boiling at 100°C for 10 min. 2.5μl proteinase K (10mg/ml; Life Technologies) was added and samples incubated at 55°C for 30 min before boiling for a further 10 min at 100°C. Samples were centrifuged briefly and 130μl of supernatant removed for qPCR. qPCR was performed in a 20μl Express SYBR GreenER (Life Technologies) reaction and run on a Roche Lightcycler. ChIP enrichment was determined as follows: ΔCT was calculated according to $\Delta CT = (CT_{(ChIP)} - [CT_{(Input)} - \log E\ (\text{Input dilution factor})])$ where E represents the specific primer efficiency value. % Enrichment was then calculated according to the formula $E^{-\Delta CT}$. qPCR was performed in technical triplicates from each of at least three or more independent biological replicates (cultures). The geometric mean of technical replicates was used as the CT value in the above formula. Error bars represent standard error of the mean enrichment/input averaged over biological replicates. Primers for qPCR analysis are listed in Table S3.

For calibrated ChIP-Seq with an internal reference, we modified the procedure described by Hu et al. (2015). Rather than *Candida glabrata*, *S. pombe* carrying Rad21-6HA (strain spAM638) or Rad21-3FLAG (spAM1863) was used as the calibration genome. For each IP, 100ml of *S. pombe* cells were grown in YES to OD_{595}=0.25-0.3, fixed and frozen as described for *S. cerevisiae*. *S. pombe* cell pellets were resuspended in 400μL of cold 1x FA lysis buffer/0.5% SDS+PI/PMSF and mixed with thawed *S. cerevisiae* pellet (approximately 50ml cells OD_{600}=1.8). Cells were processed as for ChIP-qPCR. However, instead of using Chelex, ChIP-seq samples were eluted in TES and de-crosslinked by incubating 400μl of eluate with 40μl proteinase K (10mg/ml) over night. Samples were cleaned up using the Promega Wizard Kit and eluted in 35μl dH_2O.

ChIP-Seq libraries were prepared using NEXTflex-6 DNA Barcodes in DNA LoBind tubes (Eppendorf). Input and IP DNA blunt and phosphorylated ends were generated using the Quick blunting kit (NEB), before addition of dA tails by Klenow enzyme. Adapters were ligated using T4 DNA ligase before consecutive selection of fragments >100bp then >150-200bp using AMPure beads. Libraries were amplified by PCR using NextFlex PCR primers before two further rounds of AMPure purification to collect fragments 100-250 bp in size. Library quality was assessed on a Bioanalyzer (Agilent) and quantified using Qubit before sequencing on a MiniSeq instrument (Illumina). Calculation of Occupancy Ratio (OR) and data analysis was performed as described by Hu et al. (2015). Briefly, reads were mapped to both the *S. pombe* calibration genome and *S. cerevisiae* experimental genome and the number of reads mapping to each genome was determined. Occupancy ratio was then determined using the formula Wc*IPx/Wx*IPc where W=Input; IP=ChIP; c=calibration genome (*S. pombe*) and x=experimental genome (*S. cerevisiae*). The number of reads at each position was normalized using the OR and visualised using the Integrated Genome Viewer (Broad Institute).

Immunoprecipitation

Approximately 5g (co-immunoprecipitation) or 30g (IP for mass spectrometry) of yeast cells were lysed mechanically using a Retsch RM100 electric mortar-grinder. Ground yeast were resuspended in H0.15 buffer (25mM Hepes (pH 8.0), 2mM $MgCl_2$, 0.1mM EDTA (pH 8.0), 0.5mM EGTA-KOH (pH 8.0), 15% glycerol, 0.1% NP-40, 150mM KCl) containing protease and phosphatase inhibitors (CLAAPE (2000x stock in DMSO contains 10mg/ml each of Chymostatin, Leupeptin, Aprotinin, Antipain, Pepstatin A and E64– 2x final), 2mM Pefabloc (AEBSF), 0.8 mM Na-orthovanadate, 0.2μM Microcystin LR, 1x Roche cOmplete EDTA-free protease inhibitor cocktail, 2mM NEM, 4mM beta-glycerophosphate, 2mM Na pyrophosphate, 10mM NaF) and incubated with 40U/ml benzonase (Novagen) at 4°C for 1h. Debris was removed by centrifugation and lysates were incubated for 2.5 h at 4°C with Protein G Dynabeads,

previously conjugated to mouse anti-Flag (M2, Sigma) or mouse anti-V5 (SV5-Pk1, Bio-Rad) and, for co-immunoprecipitation only, blocked for 1 h in 5% milk. Beads were washed three times in H0.15 buffer before elution at 50°C for 15 min in NuPAGE LDS sample buffer (ThermoFisher) with 5% β-mercaptoethanol.

Western Blotting

For western immunoblotting, samples were fixed in TCA, acetone-washed and whole cell extracts prepared by bead-beating in TE containing protease inhibitors before running on SDS-PAGE and transferring to nitrocellulose membrane. Antibodies used were mouse anti-Ha (HA11, Covance) at 1:1000 dilution, mouse anti-GFP (Roche) at 1:1000, mouse anti-V5 (SV5-Pk1, Bio-Rad) at 1:2000, mouse anti-Flag (M2, Sigma) at 1:1000, rabbit anti-Pgk1 (lab stock) at 1:10000, rabbit anti-Kar2 (lab stock) at 1:20000, sheep anti-mouse-HRP (GE Healthcare) at 1:5000, donkey anti-rabbit-HRP (GE Healthcare) at 1:10000, donkey anti-mouse-IRDye 800CW (LI-COR Biosciences) at 1:10000 and donkey anti-rabbit-IRDye 680RD (LI-COR Biosciences) at 1:10000. Quantitative western blotting was performed using an Odyssey CLx Infrared Imaging System (LI-COR Biosciences) and quantified using ImageStudio 5.2.5 (LI-COR Biosciences).

Mass Spectrometry

Protein samples were run on a bis-tris gel, stained with Coomassie; bands were excised and de-stained with 50mM ammonium bicarbonate and acetonitrile (ACN) and proteins were digested with trypsin. In brief, proteins were reduced in 10mM dithiothreitol for 30 min at 37°C and alkylated in 55mM iodoacetamide for 20 min at ambient temperature in the dark. They were then digested overnight at 37°C with 12.5ng/μL trypsin. Following digestion, peptides were eluted with 80% ACN + 0.1% TFA solution and concentrated until dry by vacuum. High-Select™ TiO2 Phosphopeptide Enrichment Kit (Thermo Fisher) was used according to manufacturer's instructions, with considerable sonication time needed to resuspend peptides in the Binding Buffer. Both the flow-through (containing nonphosphopeptides) and the eluate were concentrated until dry by vacuum centrifugation. The flow-through sample was resuspended in 100μL 0.1% TFA and spun onto StageTips as described previously (Rappsilber et al., 2003). Peptides were eluted from StageTips in 40μL of 80% ACN in 0.1% TFA and concentrated down to 1μL by vacuum. All samples were then prepared for LC-MS/MS analysis by diluting them to 5μL with 0.1% TFA. LC-MS-analyses were performed on an Orbitrap Fusion™ Lumos™ Tribrid™ Mass Spectrometer (Thermo Fisher Scientific, UK) coupled on-line, to an Ultimate 3000 RSLCnano Systems (Dionex, Thermo Fisher Scientific, UK). Peptides were separated on a 50cm EASY-Spray column (Thermo Fisher Scientific, UK) assembled in an EASY-Spray source (Thermo Fisher Scientific, UK) and operated at a constant temperature of 50°C. Mobile phase A consisted of 0.1% formic acid in water while mobile phase B consisted of 80% ACN and 0.1% formic acid. Peptides were loaded onto the column at a flow rate of 0.3μL/min and eluted at a flow rate of 0.2μL/min according to the following gradient: 2 to 40% buffer B in 150 min, then to 95% in 11 min. Survey scans were performed at 120,000 resolution (scan range 350-1500 m/z) with an ion target of 4.0E5. MS2 was performed in the ion trap at rapid scan mode with ion target of 2.0E4 and HCD fragmentation with normalized collision energy of 27. The isolation window in the quadrupole was set at 1.4 Thomson. Only ions with charge between 2 and 7 were selected for MS2. The MaxQuant software platform version 1.6.1.0 was used to process raw files and search was conducted against the *Saccharomyces cerevisiae* (strain SK1) complete/reference proteome set of Saccharomyces Genome Database (released in December, 2016), using the Andromeda search engine. The first search peptide tolerance was set to 20ppm while the main search peptide tolerance was set to 4.5ppm. Isotope mass tolerance was 2ppm and maximum charge of 7. A maximum of two missed cleavages were allowed. Fixed modifications: cysteine carbamidomethylation; variable modifications: oxidation of methionine, acetylation of the N-terminus, phosphorylation of serine, threonine and tyrosine. Label-free quantitation (LFQ) analysis was performed by the MaxLFQ algorithm. FDR was set to 1%.

Flow-through sample data was used to identify Sgo1 interactors. LFQ data was processed using DEP R package (Zhang et al., 2018). Imputation was performed using "MinProb" function with default parameters. Phospho-enriched sample data was used to analyse Rec8 phosphopeptides, while the flow-through sample data was used to analyse Rec8 nonphosphopeptides. First, the signal intensity of each individual Rec8 peptide was measured using Skyline (MacLean et al., 2010), with the same variable and fixed modifications set as described above. The following procedure was then applied to both phospho- and nonphosphopeptides. First, a normalization factor, derived by measuring peptide intensities of Sgo1-bound Smc3, was applied. Second, as each Rec8 residue could have been detected multiple times (because of variable modifications or mis-cleaved peptides), a sum of intensities of all observed peptides containing each Rec8 residue was generated. The logarithm of the obtained number was then taken, and the $\log_2$ difference between of sum intensity between wild-type and mutant cells was plotted. Plots in Figure 5 (A and B) show an average ratio of $\log_2$(fold enrichment over wild type) of Rec8 nonphospho- (A) and phosphopeptides (B) per amino acid, generated by averaging the ratios presented in Figures S4B and S4C. Raw data are available via ProteomeXchange with identifier PXD012627.

Immunofluorescence and Fixed-Cell Microscopy

Meiotic spindles were visualised by indirect immunofluorescence. Briefly, 200μl meiotic culture was collected, and the pellet resuspended in 3.7% formaldehyde in 0.1M KPi pH 6.4. After overnight fixation, cell pellets were washed 3 times in 1ml of 0.1M KPi pH 6.4. before resuspending in 1ml of 1.2M sorbitol-citrate. Fixed cells were resuspended in digestion mix (200 μl 1.2M sorbitol-citrate, 20 μl glusulase (Perkin Elmer) and 6 μl zymolyase (10mg/ml; AMS Biotechnology (Europe)) for at least 2 h at 30°C. Spheroplasts were washed once in 1ml sorbitol-citrate and resuspended in sorbitol-citrate before adhering to multi-well polylysine-treated slides and fixed in MeOH for 3 min, immersed in acetone for 10s and allowed to dry. Cells adhered to wells were incubated with rat anti-tubulin

primary antibody (Bio-Rad) at 1:50 dilution in PBS/BSA (1% BSA, 0.04M K_2HPO_4, 0.01M KH_2PO_4, 0.15 M NaCl, 0.1% NaN_3) for 2h, washed five times in PBS/BSA. Secondary anti-rat FITC conjugated antibody (Jackson Immunoresearch) was added at 1:100 dilution in PBS/BSA, incubated a further 2h and wells washed a further five times with PBS/BSA. The supernatant was aspirated and 3μl DAPI-MOUNT (1mg/ml p-phenylenediamine, 0.04M K_2HPO_4, 0.01M KH_2PO_4, 0.15M NaCl, 0.1% NaN_3, 0.05μg/ml DAPI, 90% glycerol) added to each well and a coverslip placed on the slide before imaging or storing at -20°C.

To visualise *CEN5* TetR-tdTomato foci, cells were fixed as previously described (Klein et al., 1999). Briefly, cells were fixed with 3.7% formaldehyde for 8 minutes before washing in 500μl PBS. Cell pellets were resuspended in 20μl PBS before scoring by fluorescence microscopy.

Live-Cell Imaging

Unless stated, live-cell imaging was performed on a DeltaVision Elite system (Applied Precision) connected to an inverted Olympus IX-71 microscope with a 100x UPlanSApo NA 1.4 oil lens. Images were taken using a Photometrics Cascade II EMCCD camera. The Deltavision system was controlled using SoftWoRx software. Live-cell imaging for Figures 2E, 2G, 2J, 3F, 3G, 6G, 6H, 7A, 7B, S2B, S2F, S2G, S2H, S3E, S3F, S6I, and S6L was performed on a Zeiss Axio Observer Z1 (Zeiss UK, Cambridge) equipped with a Hamamatsu Flash 4 sCMOS camera, Prior motorised stage and Zen 2.3 acquisition software. Enhanced resolution imaging in Figures 4A and 4B was performed on a Zeiss LSM 880 laser scanning confocal equipped with an Airyscan detector (Zeiss UK, Cambridge). A high NA oil immersion alpha Plan Apochromat x100/1.46 objective was used for enhanced resolution. Laser intensity was kept as low as possible to maintain cell viability and reduce phototoxicity.

Cells were imaged at 30°C (unless stated) on either an ONIX microfluidic perfusion platform by CellASIC or Ibidi 4-well or 8-well dishes. Cells were pre-grown in culture flasks for ~3 h before transfer to either microfluidics plates or concanavalin A-coated Ibidi dishes. In the latter case, cells were left to attach for 20 min. Imaging began about 30 min later, with images being acquired every 15 min for 12-15 h. Seven to eight z-stacks were acquired with 0.85μm spacing. For prophase block-release experiments, cells were arrested in culture flasks for 5 h before release. Drugs were added and cells left to shake for a further 5 min before transfer to microfluidics plates (Figures 5D, 5G, 7C, 7E, S1F, S3A, S6G, S6H, S7G, and S7J) or attachment to Ibidi dishes (all others). Image panels were assembled using Image Pro Premier, version 9.1 (Media Cybernetics). Images were analysed using ImageJ 1.48v (National Institutes of Health). Final image assembly was carried out using Adobe Photoshop CS5.1 and Adobe Illustrator CS5.1. Rec8-GFP intensities were measured a custom plugin for ImageJ. The first plugin "DV_DotCounter" (https://doi.org/10.5281/zenodo.2553082) applied a Z projection to each colour channel and allowed the user to select a cell of interest. Kinetochores in the red channel were identified by Yen autothreshold and their XY central coordinates, mean intensity and area recorded. The coordinates were then used to measure mean intensity in the corresponding location in the green channel, equivalent to pericentromeric Rec8-GFP. A different plugin "YeastLineProfiler" (https://doi.org/10.5281/zenodo.2560099) was used in experiments where pericentromeric cohesin was likely to be found in between kinetochores (which is thought to occur in cells that bi-orient in meiosis I but retain cohesin), the XY coordinates in the red channel were used to generate a line profile between the 2 kinetochores in both colour channels over exactly the same pixels. The 2 brightest peaks in the line profile of the green channel were calculated to give the maximum intensity value for each. Rec8-GFP intensity was measured in this manner for Figures 5F and 6E. Rec8-GFP intensity in anaphase I was measured within the first two time points after Pds1-tdTomato degradation.

QUANTIFICATION AND STATISTICAL ANALYSIS

Conventions used to indicate statistical significance between conditions are described in figure legends. All ChIP experiments were performed in at least three biological repeats. Statistical significance for ChIP experiments was calculated using the paired t-test with a two-tailed distribution. Proteomic data in Figures 5A and 5B was analysed by one-sample t-test. p-values in volcano plots (Figure S4A) were calculated using t-test. For all imaging experiments, 50 different cells were analysed per strain. To quantify signal intensity of Rec8-GFP in anaphase I, GFP channel intensity was normalised to the average background GFP signal from 50 cells as measured in wild-type meiosis II cells. Western blot quantifications (Figure S6C) were performed from four different repeats. For each repeat, the ratio of Sgo1-6Ha over wild type was calculated for any given strain and divided by the corresponding signal ratio of Kar2 (loading control) over wild type.

DATA AND SOFTWARE AVAILABILITY

ChIP-seq data has been deposited on the Genome Expression Omnibus (GEO) and can be accessed using accession numbers GSE112167 (Sgo1-6Ha), GSE112170 (Spo13-3Flag) and GSE123546 (Rec8-3Ha).

Plugins for Image J have been deposited on Github https://github.com/dkelly604/DV_DotCounter and https://github.com/dkelly604/YeastLineProfiler. The proteomics dataset is available at ProteomeXchange with identifier PXD012627.

Developmental Cell, Volume *49*

Supplemental Information

Reductional Meiosis I Chromosome Segregation Is Established by Coordination of Key Meiotic Kinases

Stefan Galander, Rachael E. Barton, Weronika E. Borek, Christos Spanos, David A. Kelly, Daniel Robertson, Juri Rappsilber, and Adèle L. Marston

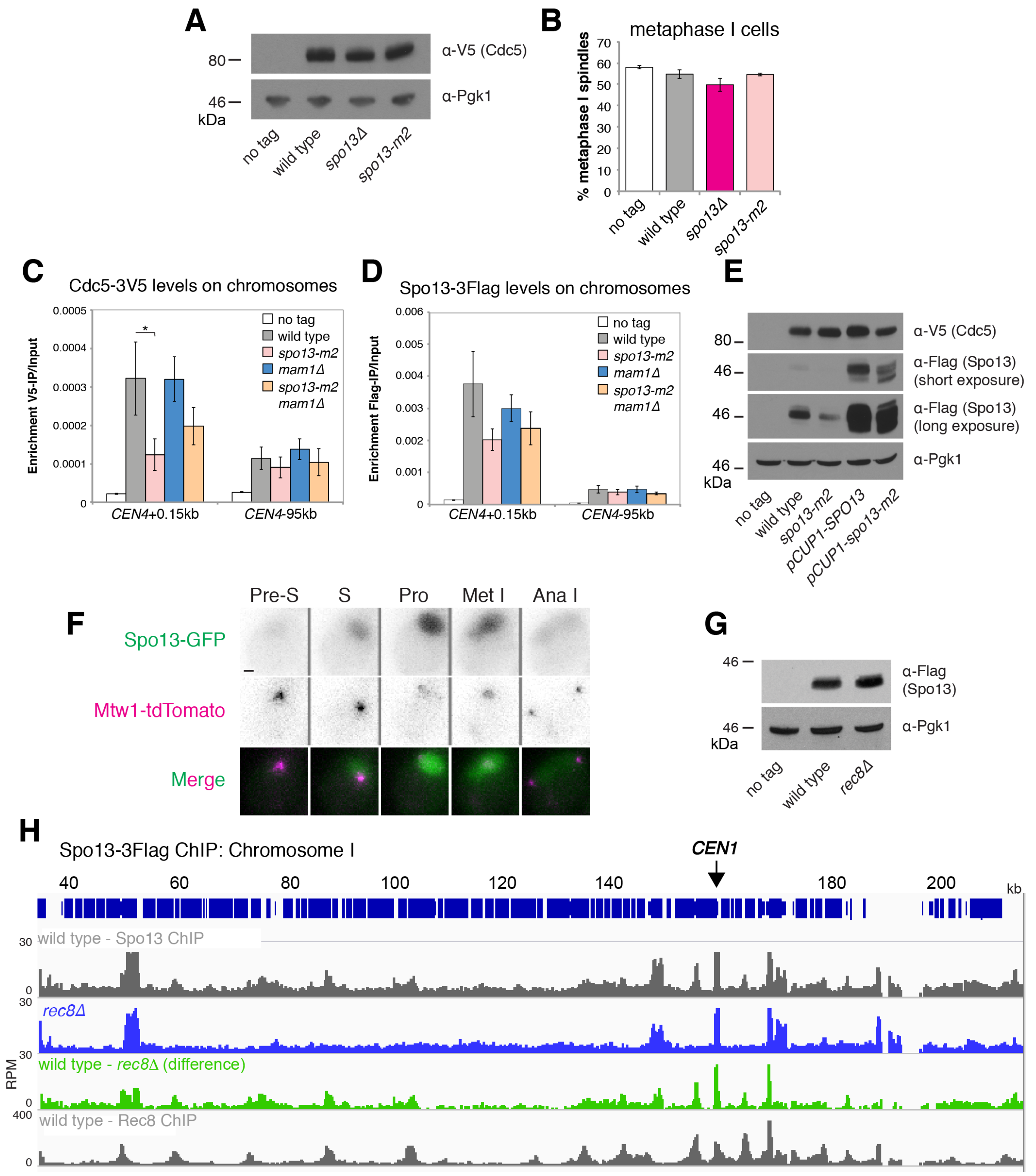

Figure S1. Spo13 and Cdc5 chromosome enrichment and cellular levels in monoorientation mutants, related to Figure 1. (A-B) Cdc5 levels and metaphase I arrest efficiency are comparable for wild type, *spo13Δ* and *spo13-m2* in the experiment in Figure 1A. (A) Immunoblots from a representative experiment show Cdc5-3V5 levels in whole cell extracts. (B) Percentage of cells with metaphase I spindles after tubulin immunofluorescence (n = 200). (C-D) Loss of centromeric Cdc5 in *spo13-m2* mutants is not caused by defective monoorientation. Mean enrichment values of Cdc5-3V5 (C) and Spo13-3Flag (D) in metaphase I with standard error bars (n = 4) (*p<0.05). (E) Immunoblot confirms Spo13 overproduction and the effect on Cdc5 levels in whole cell extracts for the experiments shown in Figure 1B and C. (F) Spo13 is present throughout the nucleus from S phase until anaphase I. Representative images of wild-type cells carrying Spo13-GFP and Mtw1-tdTomato undergoing meiosis. Scale bar, 1 µm. (G-H) Spo13 levels are comparable in wild type and *rec8Δ*, despite Rec8 being required for the association of Spo13 with some chromosomal sites. Data is from the experiment in Figures 1D and 1E. Cells were arrested in prophase. (G) Immunoblot shows Spo13 levels are comparable in wild type and *rec8Δ*. (H) ChIP-seq enrichment of Spo13-3Flag on chromosome I is shown with a different scale on the y axis to show Spo13 enrichment sites on chromosome arms. Pgk1 serves as loading control in A, E and G.

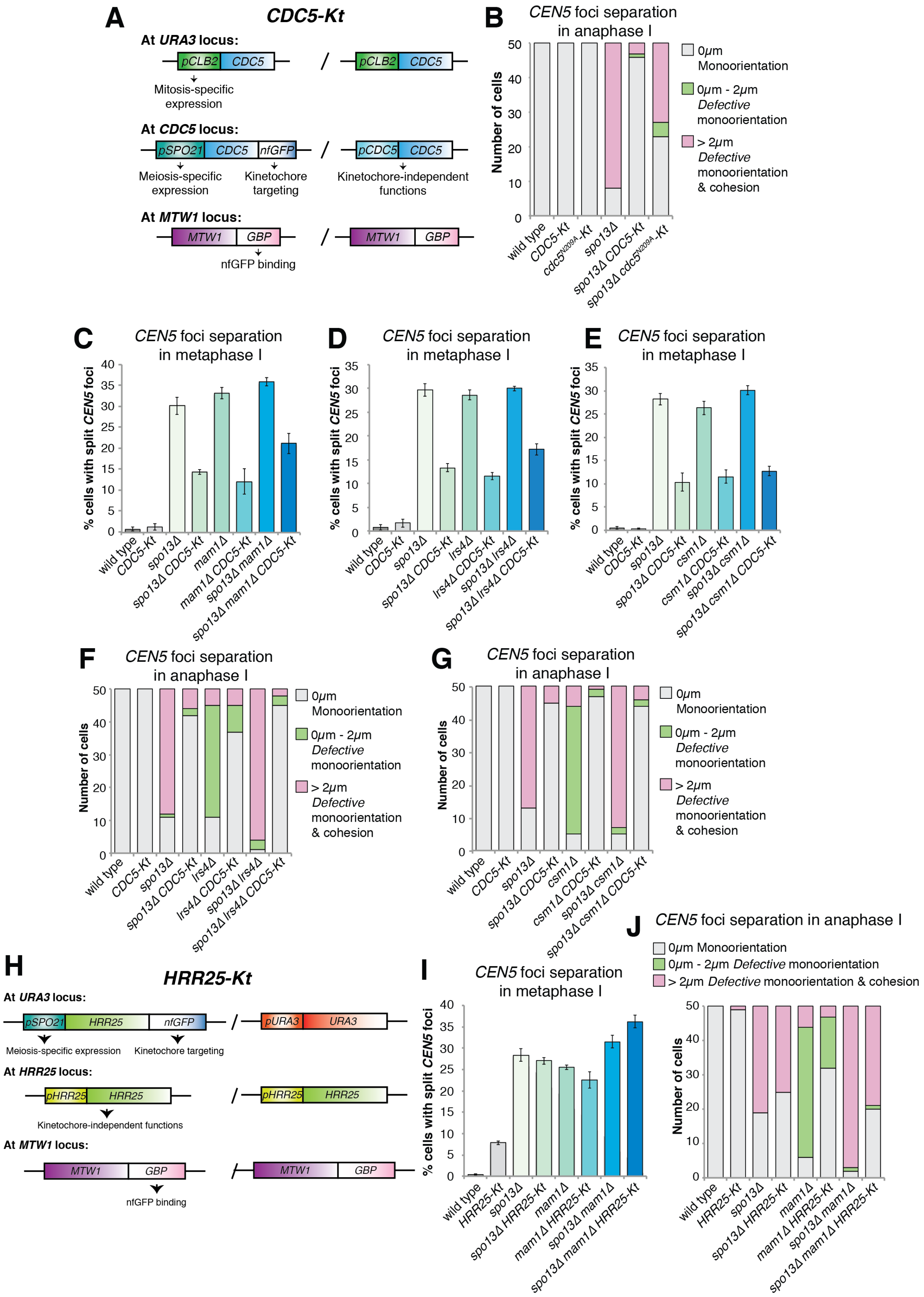

Figure S2. Cdc5-Kt, but not Hrr25-Kt, rescues monoorientation in *spo13Δ* cells, related to Figure 2. (A) Schematic representation of Cdc5 tethering setup within the genome. Cdc5 tagged with a non-

fluorescent version of GFP (Cdc5-nfGFP) (Kutrowska et al., 2007) was produced from the meiosis-specific promoter *pSPO21* and the kinetochore protein Mtw1 was tagged with GFP-binding protein (GBP), a nanobody that specifically recognizes the GFP protein (Rothbauer et al., 2006). These Cdc5-kinetochore bound cells (Cdc5-Kt) also produced untagged Cdc5 from the endogenous promoter to allow its access to other meiotic targets. (B) Kinase-defective Cdc5-Kt does not rescue the monoorientation phenotype of *spo13Δ* mutants. Frequency of *CEN5* distance categories as shown in Figure 2D is shown for the indicated genotypes after live-cell imaging with the maximum distance between two TetR-tdTomato foci measured as in Figure 2E. (C-E) Cdc5-Kt partially rescues the monoorientation defects caused by absence of monopolin complex components Mam1 (C), Lrs4 (D) and Csm1 (E). The number of cells with two distinct tdTomato foci in metaphase-I-arrested cells was scored (n = 200). Graphs show mean values of three experimental replicates with standard error bars. For panel (C), data from Figure 2B was duplicated. (F-G) Co-segregation of sister chromatids is rescued by Cdc5-Kt in *lrs4Δ* (F) and *csm1Δ* (G) mutants. Frequency of *CEN5* distance categories as shown in Figure 2D is shown for the indicated genotypes after live-cell imaging with the maximum distance between two TetR-tdTomato foci measured as in Figure 2E. (H-J) Hrr25-Kt partially rescues the monoorientation defect caused by absence of Mam1, but not Spo13. (H) Schematic representation of Hrr25 tethering setup within the genome. (I) The number of cells with two distinct tdTomato foci in metaphase-I-arrested cells was scored. Graphs show mean values of three experimental replicates with standard error bars. (J) The maximum distance between two TetR-tdTomato foci was measured within two time points after the first round of Cdc14 release for 50 cells.

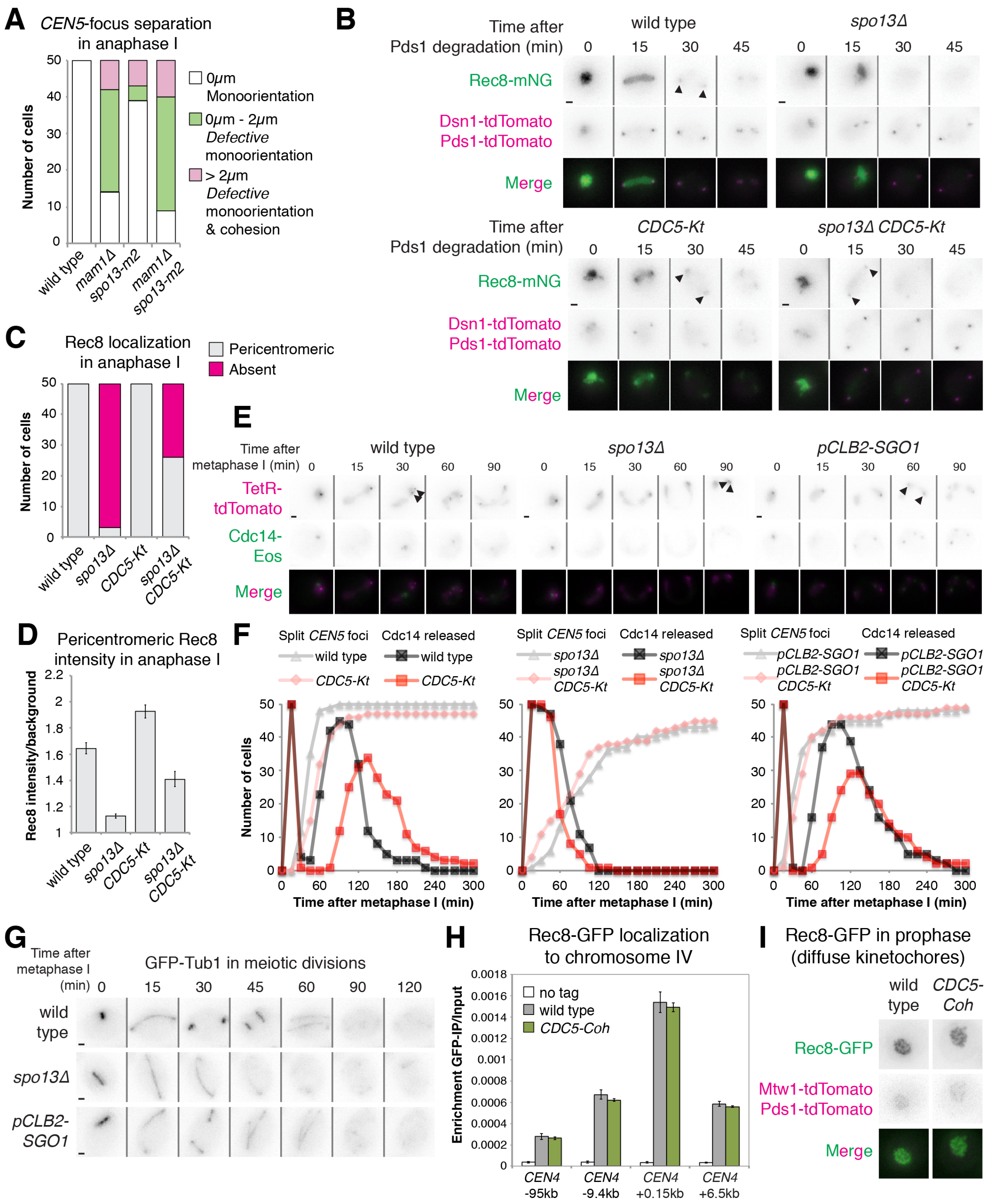

Figure S3. Cdc5-Kt does not restore cohesion in the absence of *SPO13*, related to Figure 3. (A) Effect of *spo13-m2* on monoorientation and cohesin protection. Frequency of *CEN5* distance categories after measurement of the maximum distance between two TetR-GFP foci in the time interval between Pds1-tdTomato degradation and spindle pole body duplication after live-cell imaging of 50 cells. (B-D) Cdc5-Kt increases pericentromeric cohesin in anaphase I. (B) Representative images from movies of cells carrying Rec8-mNG, Dsn1-tdTomato and Pds1-tdTomato with the indicated genotypes are shown. Scale bars represent 1μm. Arrows indicate pericentromeric Rec8-mNG. (C) Frequency of cells with the indicated pattern of Rec8-mNG localization is shown for the indicated genotypes. (D) The average intensity of pericentromeric Rec8-mNG is shown for the indicated genotypes with standard error bars. (E-F) Sister chromatid cohesion is lost in *spo13Δ CDC5-Kt* cells after anaphase I onset. Sgo1-depleted cells are shown as

a control. (E) Representative movies from live cells carrying heterozygous *CEN5*-tdTomato foci and Cdc14-mEos. Arrowheads indicate split *CEN5*-tdTomato foci. (F) Movies were scored for the presence of two *CEN5*-tdTomato foci and Cdc14 release for 300 minutes after metaphase I (time point before first Cdc14 release) at 15 min intervals. For *CEN5* foci separation, the accumulation of cells splitting foci at least once is shown. Only cells with co-segregating sister chromatids in anaphase I were scored. Note that while wild-type and *pCLB2-SGO1* cells undergo two rounds of Cdc14 release, *spo13Δ* cells exhibit only a single release of Cdc14. (G) *CEN5* foci separation in *spo13Δ* cells after anaphase I is not due to spindle forces since no metaphase II spindle forms. Representative images of meiotic spindles (GFP-Tub1) from the indicated genotypes. In contrast, meiosis II spindles form in wild type and *pCLB2-SGO1* and can account for *CEN5* foci splitting. (H-I) Rec8 loading is comparable in wild-type and *CDC5-Coh* strains. (H) ChIP-qPCR of Rec8-GFP prior to prophase. Cells were all *ndt80Δ* background and harvested for ChIP 6h after inducing sporulation. Mean values from three independent biological repeats are shown with standard error bars. (I) Representative images of Rec8-GFP from wild-type and CDC5-Coh cells in prophase, as judged by dispersed kinetochore protein Mtw1-tdTomato.

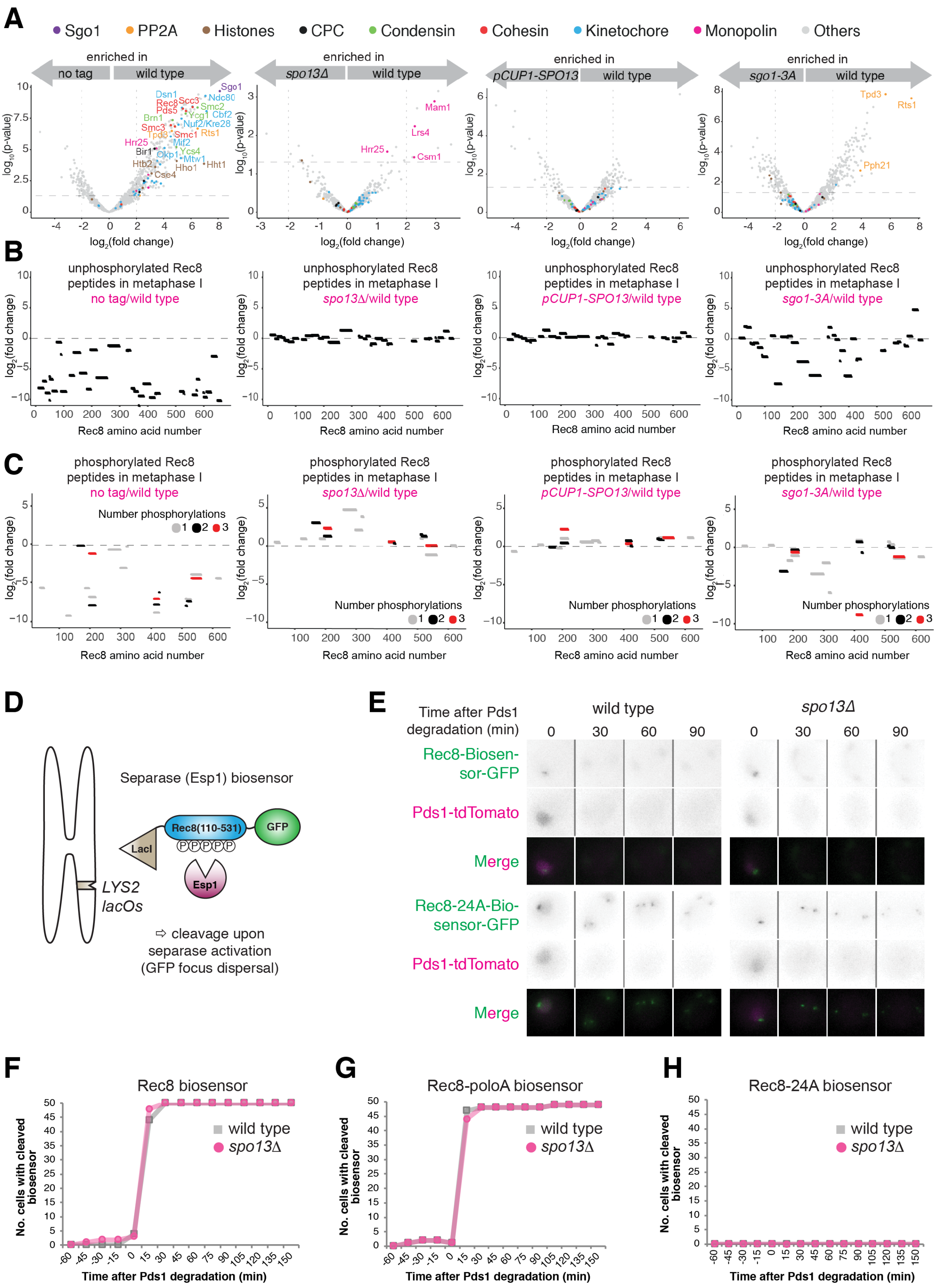

Figure S4. Spo13 and cohesin phosphorylation, related to Figure 5. (A) The pericentromeric proteome is largely unchanged upon deletion or overexpression of *SPO13*. Immunoprecipitates from Figures 5A and 5B were analysed for interacting proteins by mass spectrometry. Enrichment of proteins in wild-type cells compared with no tag, *spo13Δ*, *sgo1-3A* and *SPO13* overexpression is shown. Proteins belonging to distinct

pericentromeric protein complexes are coloured as indicated. (B-C) Cohesin phosphorylation in metaphase I is largely unaffected in the absence of *SPO13*. Analysis of unphosphorylated (B) and phosphorylated (C) Rec8 peptides co-precipitated with Sgo1-3V5, presented as relative intensities. Each bar indicates a single Rec8 peptide with amino acids of Rec8 indicated on the x-axis. For (C) mono-, di- and tri-phosphorylated forms of the same peptide are indicated by differently coloured bars. (D-H) *rec8-24A*, but not *rec8-poloA*, is a poor substrate for cleavage by separase in wild-type and *spo13Δ* cells. (D) Schematic illustration of a separase biosensor. (E) Representative images of separase biosensor cleavage in wild-type and *spo13Δ* cells. Cells with either a wild-type Rec8 or Rec8-24A biosensor fragment are shown to illustrate cleavage or non-cleavage upon Pds1 degradation. (F-H) Cells carrying a Rec8 (F), Rec8-poloA (G) or Rec8-24A (H) separase biosensor were followed by live-cell imaging and scored for diffusion of the biosensor focus at 15 min intervals.

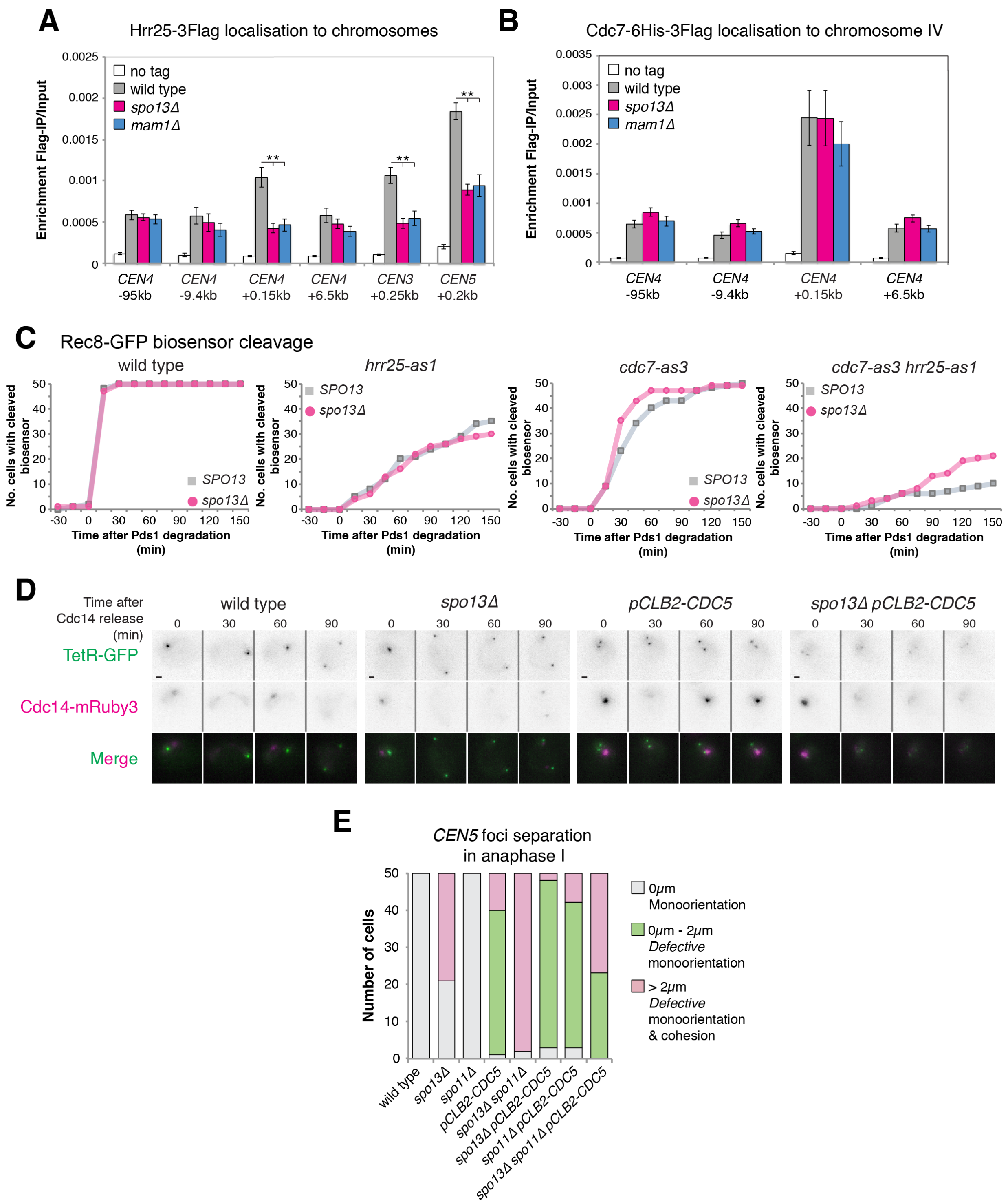

Figure S5. Spo13 represses cohesin phosphorylation, but not chromosomal association of cohesin kinases, related to Figure 6. (A-B) The association of cohesin kinases with chromosomes is not increased in *spo13Δ* mutants. (A) Chromosomal levels of Hrr25-3Flag in metaphase I were determined by anti-Flag ChIP-qPCR at the indicated sites. Mean enrichment values of Hrr25-3Flag from four experimental replicates are shown, with standard error bars (**p<0.01). (B) Chromosomal levels of Cdc7-6His-3Flag were measured by anti-Flag ChIP-qPCR. Mean enrichment values of Cdc7-6His-3Flag from four experimental replicates are shown, with standard error bars. (C) Deletion of *SPO13* enhances cleavage of a separase biosensor upon Cdc7 inhibition. Cells were scored for diffusion of the separase biosensor focus at 15 min intervals. (D-E) Sister chromatid cohesion in *spo13Δ pCLB2-CDC5* cells is partially dependent on recombination. (D) Representative images of heterozygous *CEN5*-GFP dots and *CDC14-mRuby* for indicated genotypes. (E) The maximum distance between two TetR-GFP foci was measured within two time points after the first round of Cdc14 release for 50 cells.

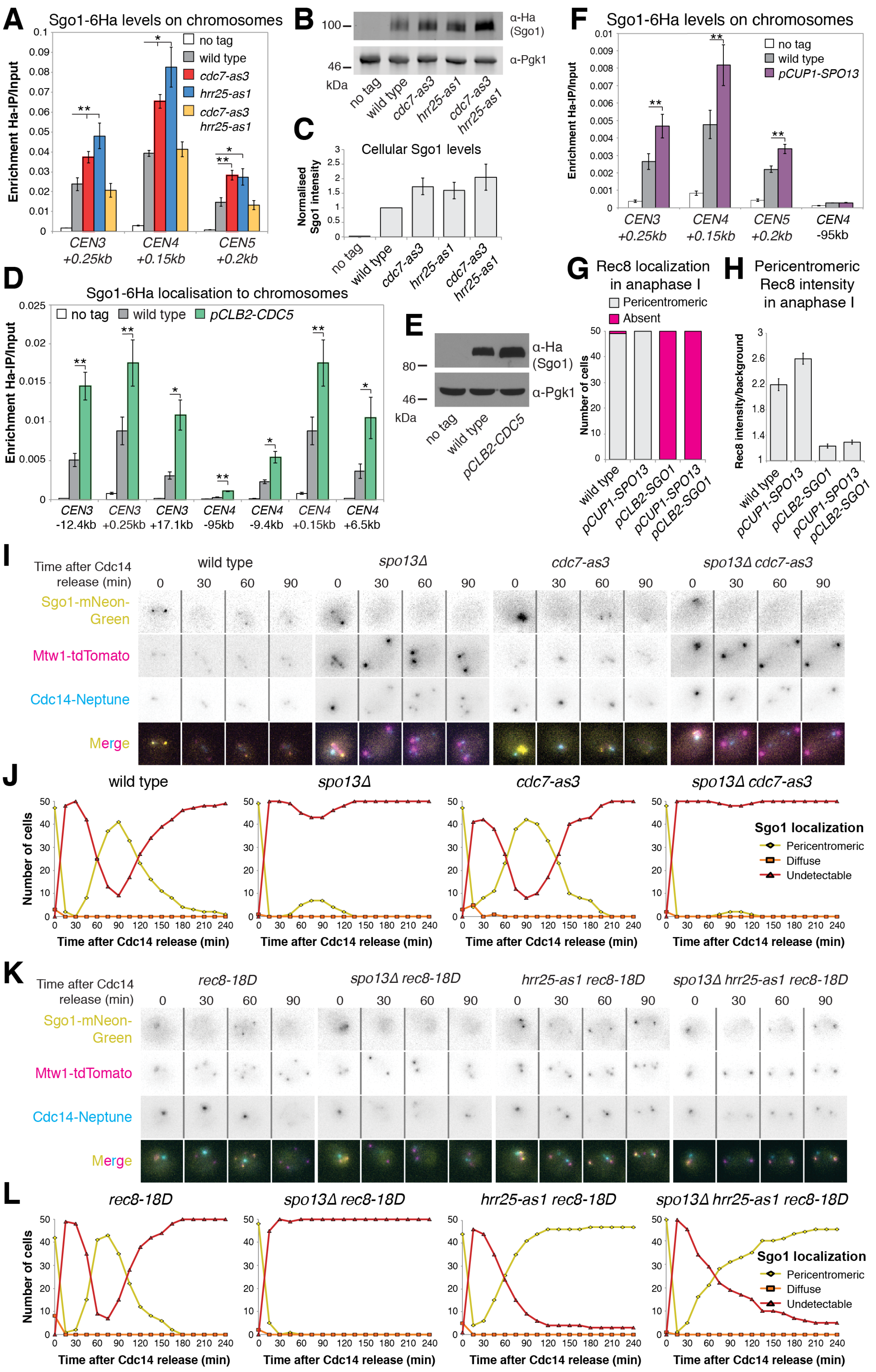

Figure S6. Chromosomal enrichment and cellular levels of Sgo1 are increased upon inhibition of single cohesin kinases, related to Figure 7. (A-C) Centromeric Sgo1 levels are increased upon inhibition of either

Cdc7 or Hrr25. (A) Chromosomal levels of Sgo1-6Ha were measured 75 min after release from a prophase I arrest by ChIP-qPCR at the indicated sites. Mean values are presented from four experimental replicates, with standard error bars (*p<0.05, **p<0.01). (B) Representative immunoblot of metaphase-I-arrested whole cell extracts as in (A) is shown with Pgk1 loading control. (C) Quantification of western blot signal. Graphs show average signal intensity relative to wild type from four biological repeats with standard error bars. (D-E) Sgo1 enrichment on chromosomes and cellular levels are increased in the absence of Cdc5. (D) Chromosomal levels of Sgo1-6Ha were measured by anti-Ha ChIP-qPCR in metaphase I at the indicated sites. Mean enrichment values of Sgo1-6Ha from four experimental replicates is shown, with standard error bars (*p<0.05, **p<0.01). (E) Immunoblot of metaphase I-arrested cultures shows slight increase in whole-cell Sgo1 levels in *pCLB2-CDC5* cells. Pgk1 serves as loading control. (F) *SPO13* overexpression increases centromeric Sgo1 levels. Mean values from four experimental repeats are shown with standard error bars (**p<0.01). (G-H) Overexpression of *SPO13* causes Sgo1-dependent overprotection of pericentromeric cohesin. (G) The presence of pericentromeric Rec8-GFP was scored in 50 anaphase I cells of the indicated genotypes from movies. (H) The intensity of Rec8-GFP was determined. (I-J) Sgo1 does not return to the pericentromere after anaphase I onset in *spo13Δ cdc7-as3* cells. (I) Representative images are shown. (J) The presence of Sgo1 was scored as pericentromeric, diffuse or absent in 50 cells for 4 h after initial Cdc14 release. (K- L) Sgo1 re-associates with the pericentromere independently of cohesin in *spo13Δ hrr25-as1* cells. (K) Representative images are shown. (L) The presence of Sgo1 was scored as pericentromeric, diffuse or absent in 50 cells for 4 h after initial Cdc14 release.

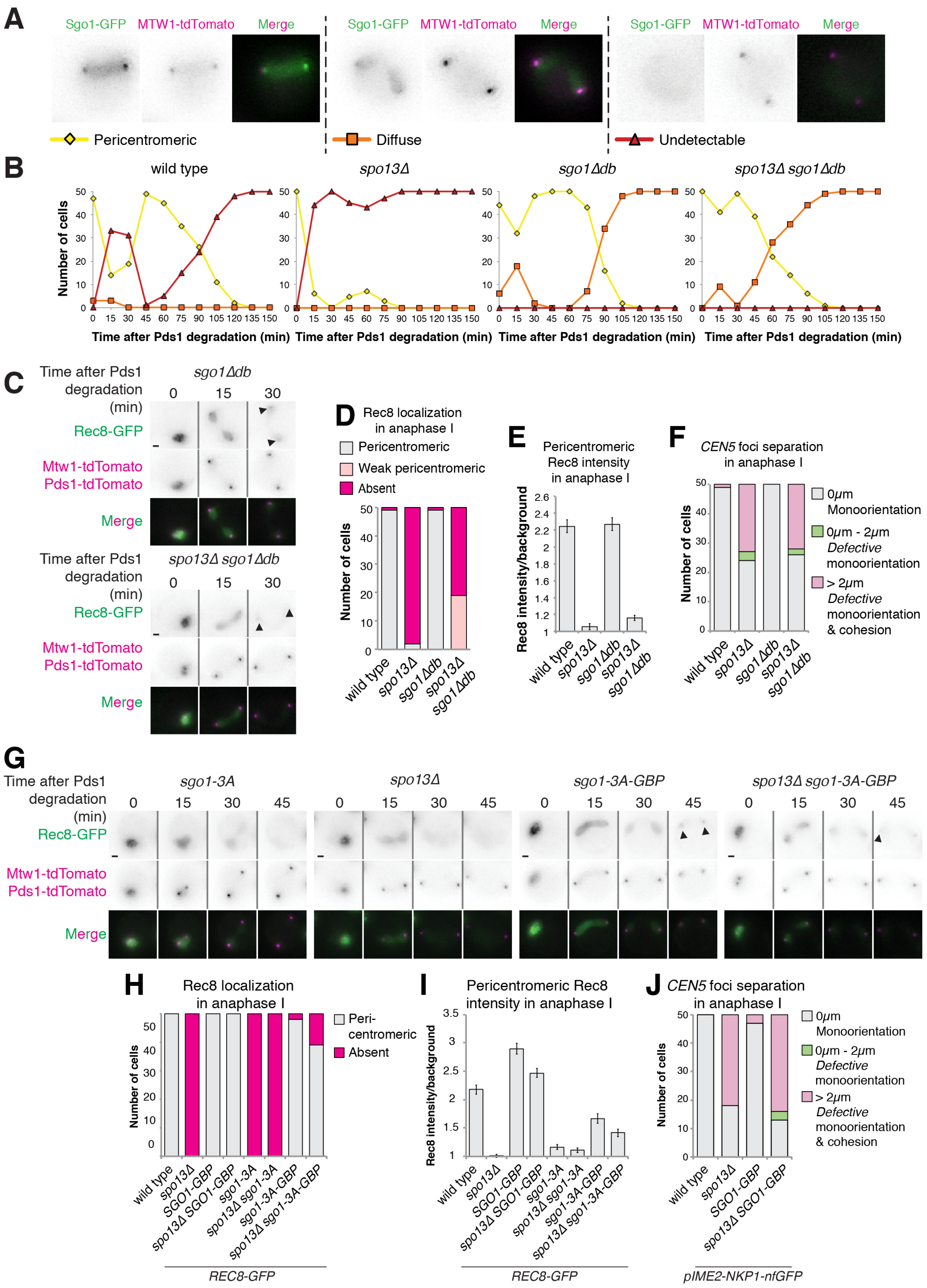

Figure S7. Chromosomal Sgo1 in *spo13Δ* anaphase I cells does not prevent sister chromatid segregation, related to Figure 7. (A-F) Preventing Sgo1 degradation does not restore sister chromatid cohesion in *spo13Δ* mutants. (A-B) Sgo1 degradation is prevented by deletion of its destruction box. (A)

Representative images of Sgo1 localization are shown in anaphase I cells. Images for pericentromeric, diffuse and undetectable localization patterns taken from *sgo1Δdb-GFP spo13Δ*, *sgo1Δdb-GFP* and *spo13Δ* cells, respectively. (B) Categories of Sgo1-GFP localization in 15 min intervals after anaphase I onset were determined from movies. (C-E) Deletion of the Sgo1 destruction box partially rescues pericentromeric cohesin in *spo13Δ* cells. (C) Representative images are shown. Scale bars represent 1µm. Arrows indicate pericentromeric cohesin. (D) The presence of pericentromeric Rec8-GFP in anaphase I was scored for 50 cells per strain. (E) Rec8-GFP intensity was measured. (F) Sister chromatid cohesion is defective in *spo13Δ sgo1Δdb* cells. *CEN5-GFP* distances were measured and scored as described in Figures 2D and 2E except that Pds1-tdTomato degradation was used as a marker for anaphase onset rather than Cdc14 release. (G-I) Tethering of Sgo1, but not Sgo1-3A to Rec8 efficiently restores pericentromeric cohesin in *spo13Δ* cells. (G) Representative live-cell images are shown. Scale bars represent 1µm. Arrows indicate pericentromeric cohesin. (H) The number of cells with pericentromeric cohesin in anaphase I is shown for the indicated genotypes after scoring 50 cells. (I) Rec8-GFP intensity was measured for 50 cells as shown in (G). Error bars represent standard error. Data for wild-type, *spo13Δ*, *SGO1-GBP* and *spo13Δ SGO1-GBP* strains shown in (H) and (I) was replicated from Figures 7D and 7E. (J) Sgo1 tethering to kinetochores does not rescue sister chromatid cohesion in *spo13Δ* cells. Cohesion functionality was determined by categorization of *CEN5-mEos* distances as described in Figures 2D and 2E.

Table S1. List of *Saccharomyces cerevisiae* strains used in this study, related to STAR methods.

Provided as an excel file.

Table S2. List of plasmids generated in this study, related to STAR methods.

Plasmid	Description	Purpose and notes
AMp1062	YIplac128-spo13-m2	*LEU2* integration plasmid carrying *spo13-m2*.
AMp1128	pFA6a-nfGFP-KanMX6	nfGFP tagging plasmid with *KanMX6* marker. nfGFP mutation is G67A. Gene also carries S65T mutation.
AMp1138	YIplac128-TetR-ymEos3.2	*LEU2* integration plasmid carrying *TetR-ymEos3.2*. Yeast codon-optimized mEos3.2 was derived from a synthetic gene construct (GeneArt).
AMp1139	pFA6a-HphMX6-pSPO21	Plasmid for promoter replacement with *pSPO21*.
AMp1147	pFA6a-GBP-His3MX6<br>*GBP* tagging plasmid with *His3MX6* marker.	GBP tagging plasmid with *His3MX6* marker.
AMp1312	pFA6a-HphMX6-pCLB2	Plasmid for promoter replacement with *pCLB2*.
AMp1351	pFA6a-mRuby3-URA3	mRuby3 tagging plasmid with *URA3* marker.
AMp1369	YIplac128-rec8-24A-GFP	*LEU2* integration plasmid carrying *rec8-24A-GFP*.
AMp1396	YIplac128-rec8-poloA-GFP	*LEU2* integration plasmid carrying *rec8-poloA-GFP*.<br>*rec8-poloA mutations are:* S136A/T173A/S179A/S197A/S199A/S215A/T249A/S285A/S386A/S387A/S410A/S421A/S465A/S466A.
AMp1398	YIplac128-rec8-poloA-3HA	*LEU2* integration plasmid carrying *rec8-poloA-3HA*.
AMp1400	pFA6a-3V5-NatMX6	3V5 tagging plasmid with *NatMX6* marker.
AMp1407	pRS303-GFP-REC8(110-531)-LacI	*HIS3* integration plasmid carrying separase biosensor corresponding to amino acids 110-531 of Rec8.
AMp1408	pRS303-GFP-rec8-24A(110-531)-LacI	*HIS3* integration plasmid carrying separase biosensor corresponding to amino acids 110-531 of rec8-24A.
AMp1409	pRS303-GFP-rec8-poloA(110-531)-LacI	*HIS3* integration plasmid carrying separase biosensor corresponding to amino acids 110-531 of rec8-poloA.
AMp1598	pFA6a-mNeonGreen-KanMX6	mNeonGreen tagging plasmid with *KanMX6* marker.
AMp1601	pFA6a-KlTRP1	Gene knockout plasmid with *Kluyveromyces lactis TRP1* marker.
AMp1603	pFA6a-mNeonGreen-KlTRP1	mNeonGreen tagging plasmid with *Kluyveromyces lactis TRP1* marker.
AMp1616	YIplac128-rec8-18D-3HA	*LEU2* integration plasmid carrying *rec8-18D-3HA*.

Plasmid	Description	Purpose and notes
AMp1620	pSPO21-HRR25-nfGFP	*URA3* integration plasmid carrying *HRR25* under control of the *SPO21* promoter and tagged with non-fluorescent GFP.

Table S3. qPCR primers used in this study, related to STAR methods.

For distances from centromeres, "-" indicates the location is upstream of the centromere, whereas "+" indicates the location is downstream of the centromere.

Chr.	Location	Distance from centromere	Primer pair	Sequence
I	Centromere	+0.02kb	8162	CGGAGCTTTCATTTCAAGCG
			8163	CTAATAAGGTCCACCGCCTA
III	Centromere	-0.05kb	8166	CATATAAACCGAACCCTTCCC
			8167	CCATATTGTTTGGCGCTGAT
III	Centromere	+0.25kb	1279	TGTTGATGGGTTTACAATTT
			1280	CTTTCAATGATTGCTCTAAATC
III	Arm	+103kb	1285	ATGGTACCTAGCTCGTGAAT
			1286	GGATTTGTCAACTTGGAACT
III	Peri-centromere	-12.4kb	4867	ATAGCAACGACGTAAGAGGT
			4868	GCCGAACTTTCTTTCCATA
III	Peri-centromere	+17.1kb	4875	TTAGCGCAGATTCAACAATA
			4876	TAGGCTTGTCATTTGTCCTA
IV	Arm	-95kb	782	AGATGAAACTCAGGCTACCA
			783	TGCAACATCGTTAGTTCTTG
IV	Centromere	+0.15kb	794	CCGAGGCTTTCATAGCTTA
			795	ACCGGAAGGAAGAATAAGAA
IV	Peri-centromere	-10.5kb	910	ACAAAGGATGATTTGTCAGG
			911	CTCTCTCCTTGGCTTGTTTA
IV	Peri-centromere	-6.5kb	1323	TAAGTCAGGACGTATTGGTG
			1324	GAGCCTTCCATAACTTCAAC
IV	Peri-centromere	-5.6kb	1327	AGATCCCTGTAGACTGGATG
			1328	ACGCGTTTGAAGAACATT
IV	Peri-centromere	-3kb	1331	TTTATCATCATCCGCATCTA
			1332	AACGAACAAGATAGGAAACC
IV	Peri-centromere	-2.2kb	1333	TGAAATCCTTCCTTTCTCAG
			1334	AATCGCATTATCAAACAGAA
IV	Peri-centromere	+10.7kb	1337	CGATAGTATTGATTGTGGGA
			1338	CCAGGAAATGCTTCTAACTT
IV	Peri-centromere	-9.4kb	4877	TACAGCAAATGTTGGTGATT
			4878	ACCTGCTTGTTCAACTCTCT
IV	Peri-centromere	+6.5kb	4885	AGAAACCACCCATAATTGAG
			4886	ACGATAGTCAAATTTCCGTT
V	Centromere	+0.05kb	8168	AAGTCTTGTTTAGTGCAAGCC
			8169	ACCGCATTTCCTTGATTTACTG
V	Centromere	+0.2kb	945	TGAAGGTGAGCTTAAGACAG
			946	CAACCATGTTCGTAGCTAAA
V	Arm	-84kb	949	CTACGGTAAATCTGGGTAGG
			950	TCCACTATCAAGTCACCAGA
